# FFAR2 activation induces IL-22-dependent intestinal immune responses to suppress CNS autoimmunity

**DOI:** 10.1038/s44321-026-00517-2

**Published:** 2026-09-03

**Authors:** Bojan Jevtić, Milica Lazarević, Goran Stegnjaić, Suzana Stanisavljević, Ivan Pilipović, Neda Nikolovski, Zorana Milosavljević, Miljana Momčilović, Ivan Koprivica, Natalija Jonić, Dragica Mićanović, Nataša Radulović, Maša Marković, Ksenija Mirković, Danijela Mišić, Uroš Gašić, Filip Nikolić, Nikola Veselinović, Henrike Körner, Lukas Grimm, Dmitri Lodygin, Alexander Flügel, Graeme Fraser, Tamara Saksida, Mirjana Dimitrijević, Ivana Stojanović, Đorđe Miljković

**Affiliations:** 1 Department of Immunology, Institute for Biological Research “Siniša Stanković” - National Institute of the Republic of Serbia, University of Belgrade, Belgrade, Serbia; 2 Department of Plant Physiology, Institute for Biological Research “Siniša Stanković” - National Institute of the Republic of Serbia, University of Belgrade, Belgrade, Serbia; 3 Neurology Clinic, Clinical Center of Serbia, Belgrade, Serbia; 4 Institute for Neuroimmunology and Multiple Sclerosis Research, University Medical Center Göttingen, Göttingen, Germany; 5 Epics Therapeutics S.A, Gosselies, Belgium

**Keywords:** Digestive System, Immunology, Neuroscience

## Abstract

Short-chain fatty acids (SCFA) protect against central nervous system (CNS) autoimmunity, but the receptor-mediated mechanisms underlying these effects remain poorly understood, limiting their therapeutic exploitation. Here, we show that selective oral activation of free fatty acid receptor 2 (FFAR2) with the synthetic agonist Cpd1 effectively ameliorates established experimental autoimmune encephalomyelitis (EAE), demonstrating therapeutic efficacy after the onset of the autoimmune response. Cpd1 treatment promoted regulatory T cells and type 3 innate lymphoid cells in the intestine while reducing inflammatory infiltration in the CNS, indicating that modulation of intestinal immunity is sufficient to restrain neuroinflammation. Transcriptomic analysis of intestinal CD4⁺ T cells revealed broad suppression of pathways controlling T-cell activation and proliferation. Mechanistically, the therapeutic effects of Cpd1 required interleukin-22 (IL-22), as antibody-mediated IL-22 neutralization abolished disease protection, identifying IL-22 as a key downstream effector of FFAR2 signaling. Together, these findings establish selective FFAR2 agonism as a tractable strategy to therapeutically modulate the gut-CNS immune axis and identify FFAR2 as a promising target for the treatment of multiple sclerosis and other autoimmune diseases.

## Synopsis

Free Fatty Acid Receptor 2 (FFAR2) is a receptor for short chain fatty acids, and is expressed on immune cells, including T cells and innate lymphoid cells (ILC). Specific agonism of FFAR2 by oral administration of a synthetic compound Cpd1 is effective in the treatment of experimental autoimmune encephalomyelitis (EAE), delineating a new target for the modulation of CNS autoimmunity.

- Ongoing EAE is suppressed by Cpd1 administration.
- Demyelination and neurodegeneration in the spinal cord are reduced.
- Encephalitogenic T helper (Th) cells are restrained both in the intestine and within the CNS.
- Intestinal Treg and ILC3 are identified as the main cellular targets for the suppressive effect of Cpd1 in EAE.
- The suppressive effect of Cpd1 is highly dependent on IL-22.

## Abstract (web summary)

Free Fatty Acid Receptor 2 (FFAR2) is a receptor for short chain fatty acids, and is expressed on immune cells, including T cells and innate lymphoid cells (ILC). Specific agonism of FFAR2 by oral administration of a synthetic compound Cpd1 is effective in the treatment of experimental autoimmune encephalomyelitis (EAE), delineating a new target for the modulation of CNS autoimmunity.

## Graphical abstract

**Figure:**
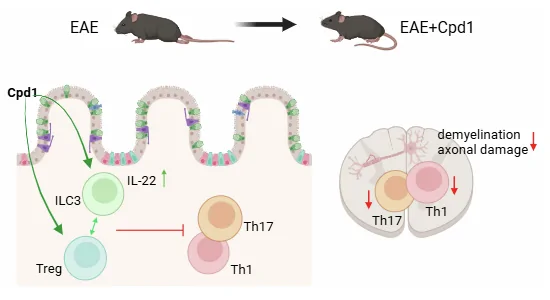

The paper explained **Problem**

Multiple sclerosis is an incurable autoimmune disease of the central nervous system. Short-chain fatty acids are products of gut bacteria. They act on immune cells, and they have been shown to be beneficial in experimental systems exploring therapeutic approaches in autoimmune diseases, including multiple sclerosis. However, more data is needed before translating the results to the therapy of multiple sclerosis by short-chain fatty acids or other molecules mimicking their effects.

**Results**

This paper shows that a synthetic compound designed to target a receptor for short-chain fatty acids efficiently ameliorates multiple sclerosis-like disease in experimental animals. Regulatory populations of gut immune cells are crucially involved in the observed effect, while interleukin-22 is identified as the main mediator of their immunomodulatory activity.

**Impact**

This study implies that specific targeting of a receptor for short-chain fatty acids could be a novel approach in the therapy of multiple sclerosis and other autoimmune diseases.

## Introduction

Intestinal immune cells and microbiota are increasingly considered as important players in the pathogenesis of autoimmune diseases, including multiple sclerosis (MS). It is firmly established that the activation of CD4^+^ T cells of T helper (Th)1 and/or Th17 phenotype is the pivotal step in the initiation of MS pathogenesis (Sospedra and Martin, 2016). Furthermore, there is evidence that the primary activation of Th1/Th17 cells occurs in the gut immune compartments where molecular mimicry and/or loss of gut barrier integrity play a major role in disease initiation (Planas et al, 2018; Stewart et al, 2018; Wekerle, 2017; Miyauchi et al, 2020). The small intestine may be a place of autoreactive T cell modulation, whereupon T cells originating from lymphoid organs migrate to the gut wherein their pathogenic properties are enhanced, or alternatively, suppressed (Miyauchi et al, 2020; Esplugues et al, 2011; Lee et al, 2011; Ivanov et al, 2008; Berer et al, 2014; Farkas et al, 2015). Notably, effector Th17 cells can be eliminated from the gut or reprogrammed within the small intestine to become regulatory cells (Esplugues et al, 2011).

Innate lymphoid cells type 3 (ILC3) are enriched in the gut and are critical regulators of gut homeostasis. These cells closely interact with the major regulatory population of T cells, comprised of CD4^+^FoxP3^+^ T cells (Treg), as well as Th1 and Th17 cells, thereby contributing to the regulation of the intestinal immune milieu (Kunkl et al, 2020; Lyu et al, 2022). Intestinal ILC3 are modulated by short-chain fatty acids (SCFA) derived from dietary fibers metabolized by gut microbiota. SCFA acting through the free fatty acid receptor 2 (FFAR2, also known as G-protein coupled receptor 43 - GPR43) stimulates ILC3 proliferation and interleukin (IL)-22 production in the small intestine (Di Luccia et al, 2019; Sepahi et al, 2021). Accordingly, ILC3 deficient for FFAR2 exhibit reduced homeostatic proliferation and production of IL-22 (Chun et al, 2019). Importantly, IL-22 was identified as a major player in gut immune homeostasis and intestinal barrier fitness (Keir et al, 2020). Treg activity is also increased in response to various stimuli originating from the gut lumen, including SCFA (Smith et al, 2013). Notably, ILC3 and Treg have the highest expression of FFAR2 among intestinal immune cells (Chun et al, 2019; Smith et al, 2013).

Cpd1 is a selective, potent FFAR2 agonist that has been shown to counteract intestinal inflammation in various in vivo models of colitis (Chun et al, 2019). Additionally, we have previously shown that Cpd1 ameliorates experimental autoimmune encephalomyelitis (EAE) when applied in the inductive phase of the disease (Lazarević et al, 2024). In this study, we extend the reported pharmacology of Cpd1 and explore its effect on established central nervous system (CNS) autoimmunity, i.e., the compound is applied in the effector phase of EAE. The relevant immune cell populations, including pathogenic (Th1 and Th17) and regulatory (Treg, ILC3) cells, were assessed both phenotypically and functionally in the spinal cord and small intestine lamina propria (SILP) of EAE mice treated with Cpd1. RNA sequencing of CD4^+^ T cells isolated from the SILP was performed. The effects of Cpd1 on gut microbiota composition and SCFA levels were also investigated.

## Results

### Cpd1 ameliorates EAE

**Figure EV1:**
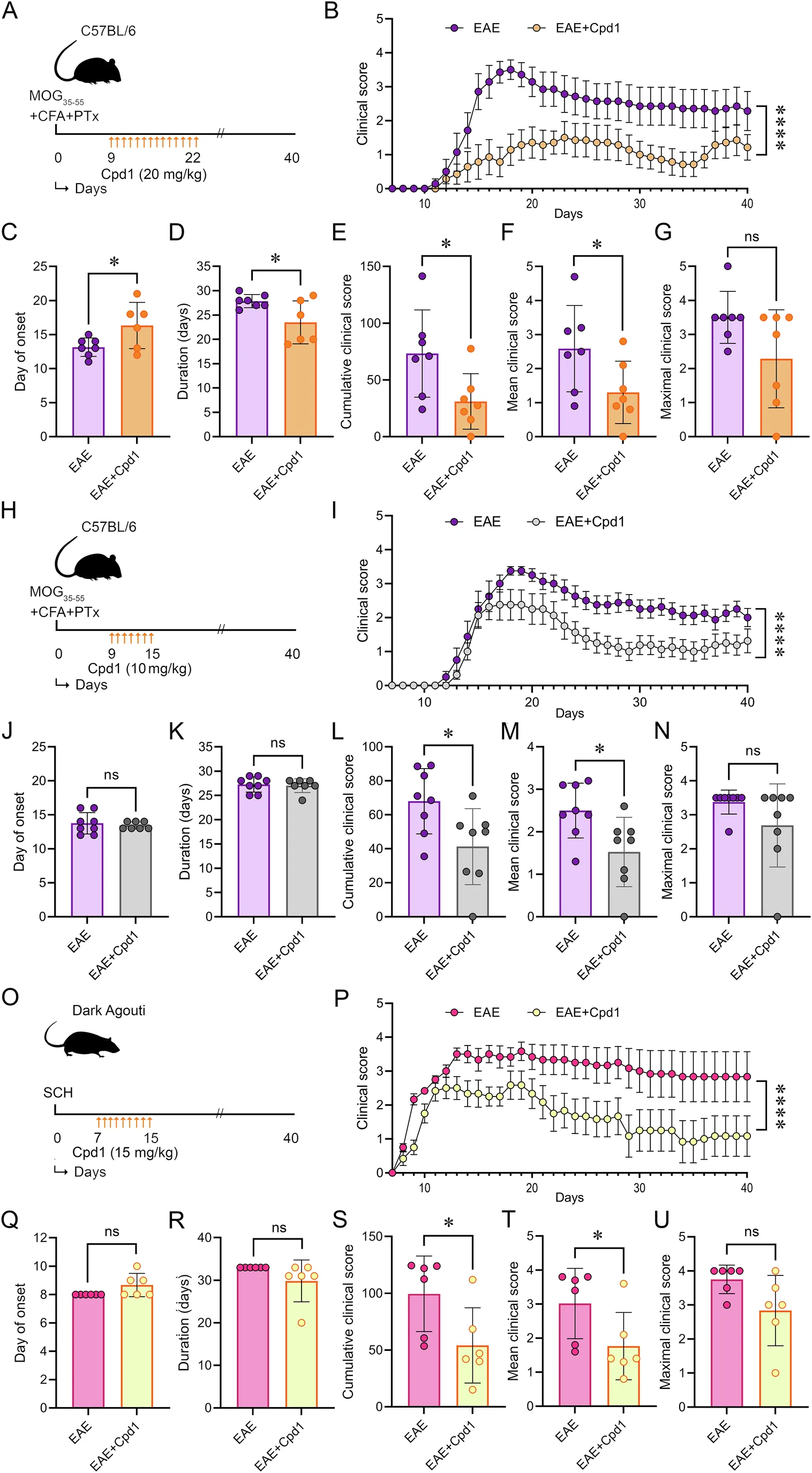
Effect of Cpd1 on EAE. EAE mice were treated orally with 20 mg/kg body weight Cpd1 (EAE+Cpd1, *n* = 7) or vehicle (EAE, *n* = 7) (days 9–22) (**A**). The severity of EAE was assessed by daily clinical scores up to day 40 (**B**), day of onset (**C**), duration of disease (**D**), cumulative clinical score (**E**), mean clinical score (**F**), and maximal clinical score (**G**). EAE mice were treated orally with 10 mg/kg body weight Cpd1 (EAE+Cpd1, *n* = 8) or vehicle (EAE, *n* = 8) (days 9 to 15) (**H**). The severity of EAE was assessed by daily clinical scores up to day 40 (**I**), day of onset (**J**), duration of disease (**K**), cumulative clinical score (**L**), mean clinical score (**M**), and maximal clinical score (**N**). EAE was induced in Dark Agouti rats by immunization with spinal cord homogenate, and rats were treated orally with 15 mg/kg body weight Cpd1 (EAE+Cpd1, *n* = 6) or vehicle (EAE, *n* = 6) (days 7–15) (**O**). The severity of EAE was assessed by daily clinical scores up to day 40 (**P**), day of onset (**Q**), duration of disease (**R**), cumulative clinical score (**S**), mean clinical score (**T**), and maximal clinical score (**U**). The data presented are biological replicates from one of two independent experiments. Data are presented as mean ± SEM (**B**, **I**, **P**), or mean ± SD (**C**–**G**, **J**–**N**, **Q**–**U**), and analyzed by two-way ANOVA with Bonferroni’s multiple comparison test (**B**, **I**, **P**), or Student’s *t* test (**C**–**G**, **L**, **M**, **S**, **T**), or Mann–Whitney test (**J**, **K**, **N**, **Q**, **R**, **U**). *P < 0.05, ****P < 0.0001, ns, not significant.

**Figure 1:**
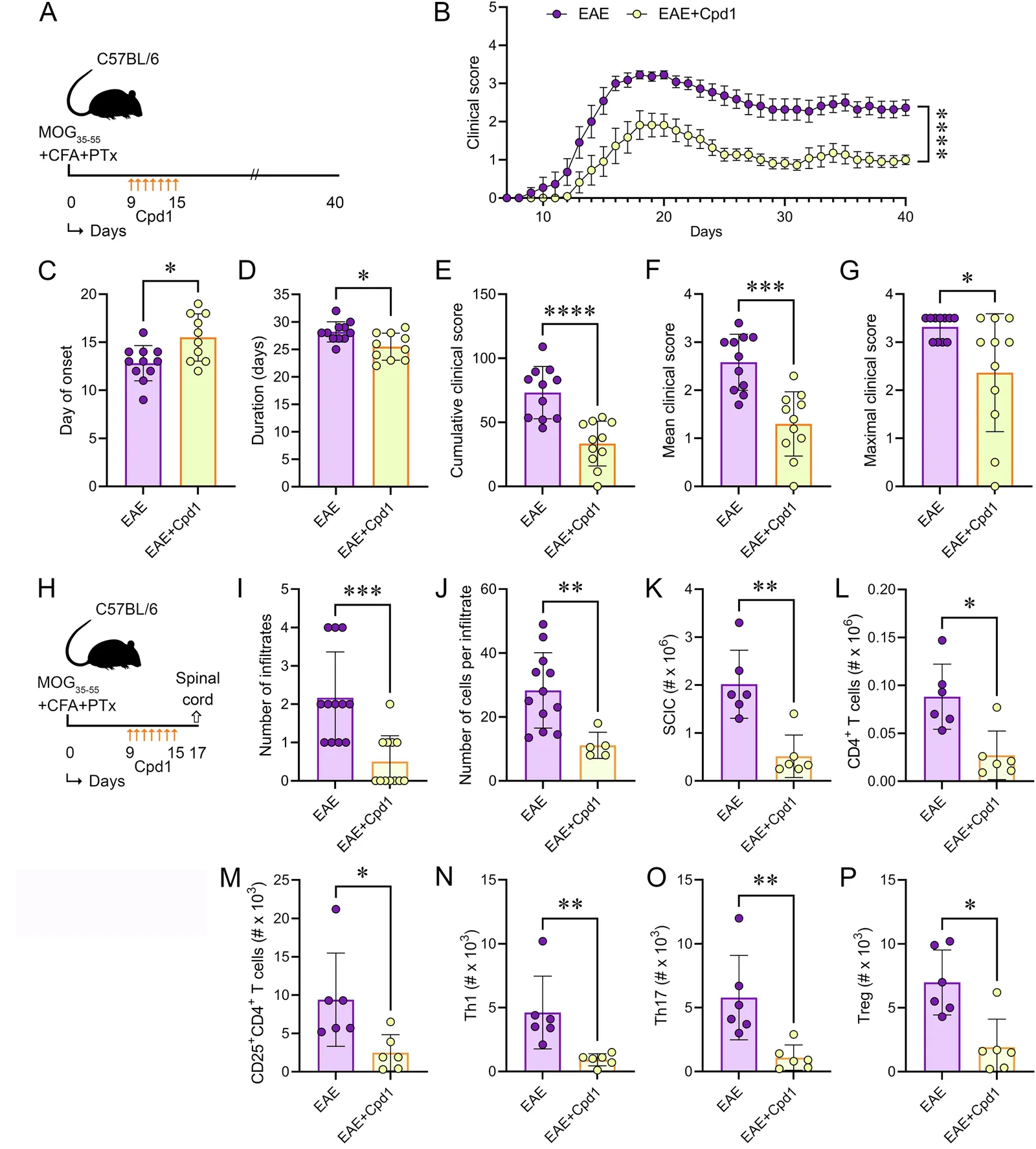
Effect of Cpd1 on EAE. EAE mice were treated orally with 20 mg/kg body weight Cpd1 (EAE+Cpd1, *n* = 11) or vehicle (EAE, *n* = 11) (days 9–15) (**A**). The severity of EAE was assessed by daily clinical scores up to day 40 (**B**), day of onset (**C**), duration of disease (**D**), cumulative clinical score (**E**), mean clinical score (**F**), and maximal clinical score (**G**). Mice were sacrificed on day 17 (**H**). The number of infiltrates per spinal cord section (**I**), and the number of cells per infiltrate (**J**) were determined in histological sections of spinal cords (*n* = 12 per group). Spinal cord immune cells (SCIC) were isolated on day 17 after the immunization (**K**–**P**). Numbers of SCIC (**K**), CD4^+^ T cells (**L**), CD4^+^CD25^+^ T cells (**M**), Th1 cells (CD4^+^IFN-γ^+^, **N**), Th17 cells (CD4^+^IL-17^+^, **O**), and Treg (CD4^+^CD25^+^FoxP3^+^, **P**) were determined (*n* = 6 per group). Data presented are biological replicates from one of three to six independent experiments. Data are presented as mean ± SEM (**B**), or mean ± SD (**C**–**G**, **I**–**P**) and analyzed by two-way ANOVA with Bonferroni’s multiple comparison test (**B**), or Student’s *t* test (**C**–**F**, **J**, **O**) or Mann–Whitney *U* test (**G**, **I**, **K**–**N**, **P**). *P < 0.05, **P < 0.01, ***P < 0.001, ****P < 0.0001. Source data are available online for this figure.

**Figure EV2:**
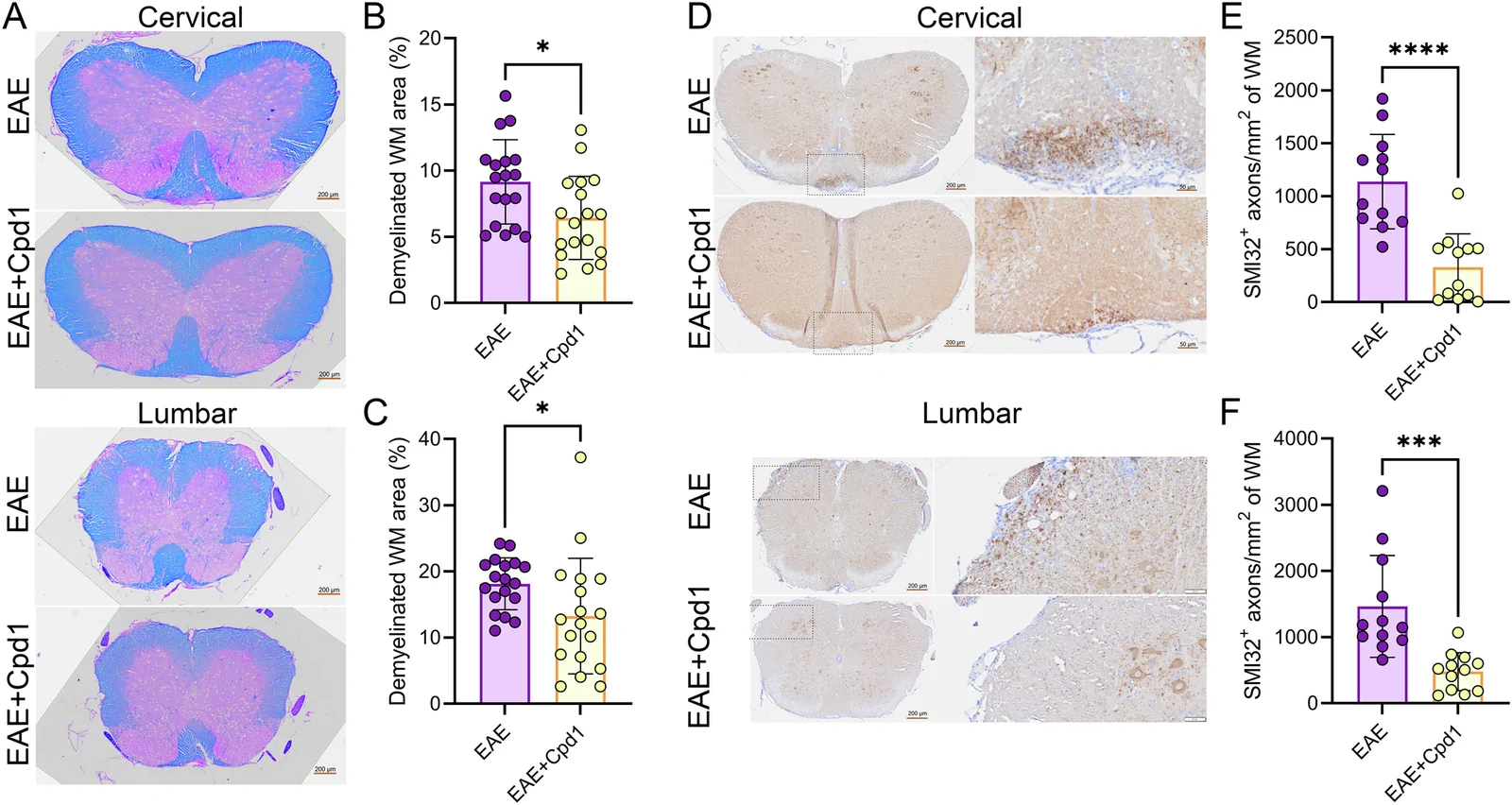
Effect of Cpd1 on demyelination and axonal damage in EAE. EAE mice were treated orally with 20 mg/kg body weight Cpd1 (EAE+Cpd1, *n* = 11) or vehicle (EAE, *n* = 11) (days 9–15). Mice were sacrificed on day 17. LFB representative images of spinal cord sections are presented (**A**). Quantification of LFB in cervical (**B**) and lumbar spinal cord sections (**C**) (3 mice, 6 sections/mouse, *n* = 18). SMI32 immunostaining representative images of spinal cord sections are presented (**D**). Quantification of SMI32 immunostaining in cervical (**E**) and lumbar spinal cord sections (**F**) (3 mice, 4 sections/mouse, *n* = 12). The data presented are biological replicates from one experiment. Data are presented as mean ± SD and analyzed by Student’s *t* test. *P < 0.05, ***P < 0.001, ****P < 0.0001.

**Figure EV3:**
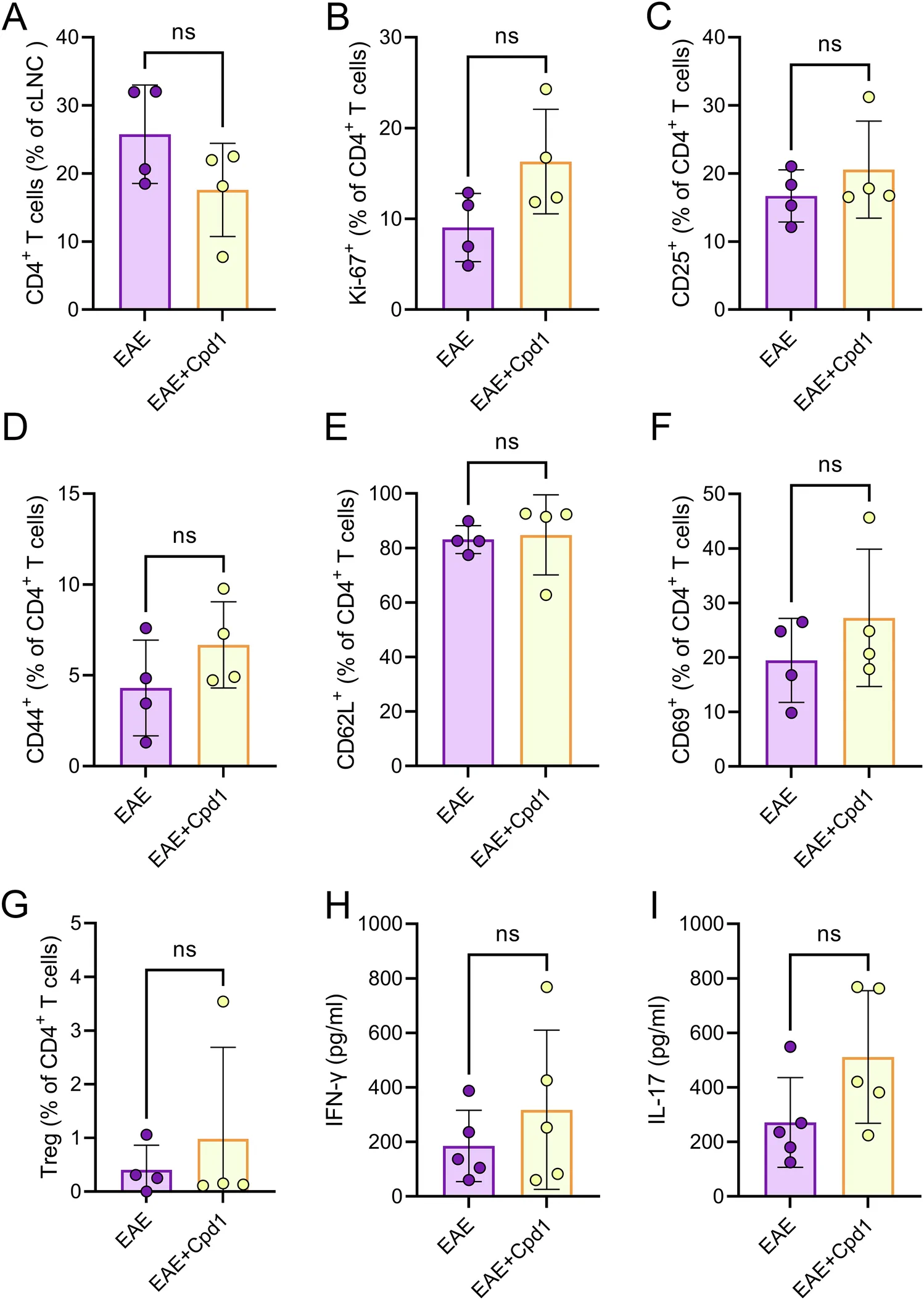
Effect of Cpd1 on lymph node cells. EAE mice were treated orally with 20 mg/kg body weight Cpd1 (EAE+Cpd1, *n* = 11) or vehicle (EAE, *n* = 11) (days 9–15). Cervical lymph node cells (cLNC) were isolated on day 17 after the immunization (**A**–**G**). Percentage of CD4^+^ T cells (**A**), Ki67^+^ cells (**B**), CD25^+^ cells (**C**), CD44^+^ cells (**D**), CD62L^+^ cells (**E**), CD69^+^ cells (**F**), and Treg (**G**). Inguinal lymph node cells were isolated on day 12 after the immunization (**H**, **I**) and stimulated with MOG_35-55_ for 24 h. Subsequently, ELISA was performed for IFN-γ (**H**) and IL-17 (**I**). The data presented are biological replicates from one of two independent experiments. Data are presented as mean ± SD (*n* = 4 in (**A**–**G**), *n* = 5 in (**H**, **I**)) and analyzed by Student’s *t* test. ns not significant.

To determine the efficiency of FFAR2 agonism in a model of CNS autoimmunity, MOG_35-55_-induced chronic EAE in C57BL/6 mice was used. Oral administration of Cpd1 (20 mg/kg), initiated one day prior to the expected onset of clinical signs and continued for 13 days, resulted in amelioration of EAE symptoms in mice (Fig. EV1A–G). Shorter treatment with the same dose, lasting for 7 days, was equally effective in ameliorating EAE (Fig. 1A–G), while a lower dose of Cpd1 (10 mg/kg) was also effective, albeit to a lesser extent (Fig. EV1H–N). The effect of Cpd1 on CNS autoimmunity was additionally confirmed in another EAE variant, i.e., in spinal cord tissue homogenate-induced EAE in Dark Agouti rats (Fig. EV1O–U). We focused on (MOG)_35-55_-induced chronic EAE in C57BL/6 mice treated with Cpd1 at a dose of 20 mg/kg for seven days in all further experiments. The effect of Cpd1 on EAE was coincident with a reduced infiltration of immune cells into the spinal cord (Fig. 1H–K). In-depth histopathological analysis showed that Cpd1 treatment reduced demyelination and axonal damage as assessed by LFB and SMI32 immunostaining, respectively (Fig. EV2A–F). Further, the number of CD4^+^, CD4^+^CD25^+^, Th1, Th17 cells, and Treg was lower in the spinal cord of Cpd1-treated EAE mice (Fig. 1L–P). Additional analysis was performed on immune cells from lymph nodes, but no effect was observed on the proportion of CD4^+^ T cells and Treg, percentage of activated or proliferating CD4^+^ T cells, or on the release of IFN-γ and IL-17 from lymph node cells under the influence of Cpd1 (Fig. EV3). Thus, Cpd1 was efficient in ameliorating EAE through downregulation of inflammation in the CNS.

### Cpd1 exerts immunoregulatory effects in EAE SILP

**Figure 2:**
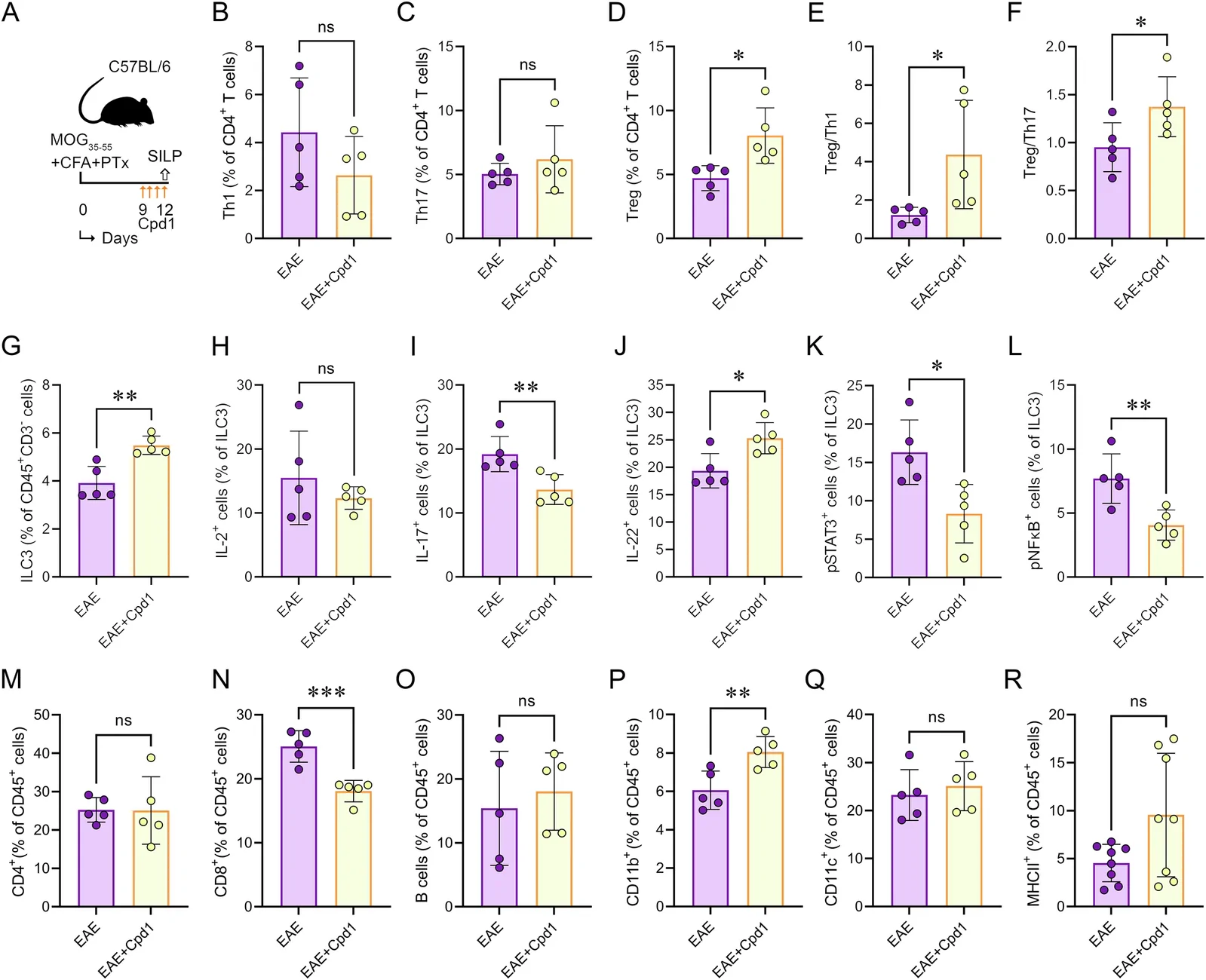
Effect of Cpd1 on SILP immune cells in EAE. EAE mice were treated orally with 20 mg/kg body weight Cpd1 (EAE+Cpd1) or vehicle (EAE) (days 9–15). SILP immune cells were isolated on day 12 after immunization (**A**). Percentage of Th1 cells (CD4^+^T-bet^+^, (**B**)), percentage of Th17 cells (CD4^+^RORγt^+^, (**C**)), percentage of Treg (CD4^+^CD25^+^FoxP3^+^, (**D**)), Treg/Th1 cell ratio (**E**), Treg/Th17 cell ratio (**F**), percentage of ILC3 (**G**), percentage of IL-2^+^ ILC3 (**H**), percentage of IL-17^+^ ILC3 (**I**), percentage of IL-22^+^ ILC3 (**J**), percentage of pSTAT3^+^ ILC3 (**K**), percentage of pNFκB^+^ ILC3 (**L**), percentage of CD4^+^ T cells (**M**), percentage of CD8^+^ T cells (**N**), percentage of B cells (**O**), percentage of CD11b^+^ cells (**P**), percentage of CD11c^+^ cells (**Q**), percentage of MHCII^+^ cells (**R**) were determined. The data presented are biological replicates from one of three independent experiments. Data are presented as mean ± SD (*n* = 5 in (**B**–**Q**), *n* = 7 in (**R**)) and analyzed by Student’s *t* test. *P < 0.05, **P < 0.01, ***P < 0.001, ns, not significant. Source data are available online for this figure.

**Figure EV4:**
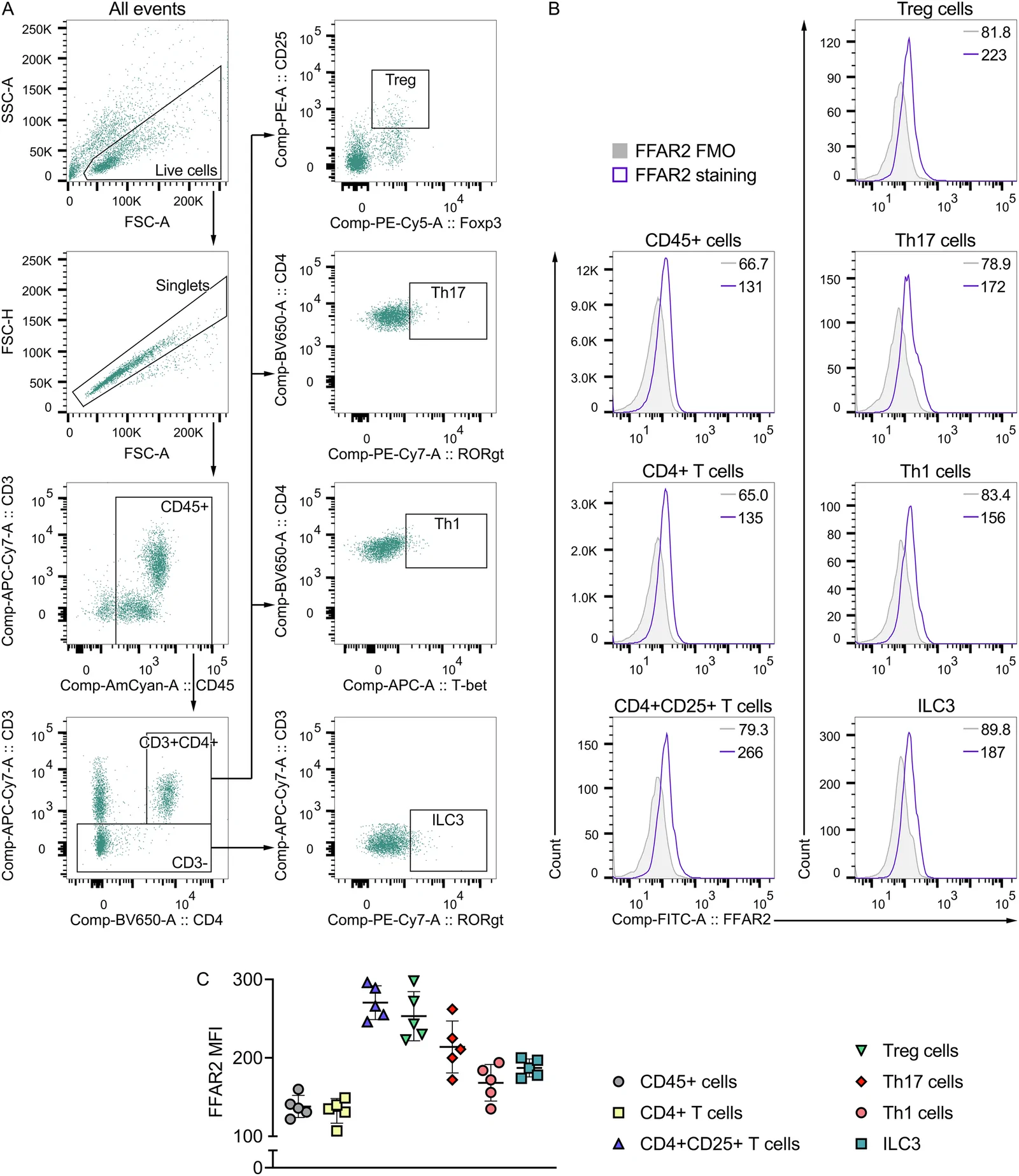
FFAR2 expression in SILP immune cells. Immune cells were isolated from the SILP of mice on day 12 after EAE immunization and analyzed by flow cytometry. Gating strategy used to analyze SILP immune cells (CD45^+^) for CD4^+^ T-lymphocyte (CD3^+^CD4^+^) subsets, i.e. activated (CD25^+^), Treg (CD25^+^Foxp3^+^), Th17 (RORγt^+^) and Th1 (T-bet^+^) cells, and ILC3 (CD3^−^RORγt^+^) (**A**). Gating boundary for activated CD25^+^ cells within CD4^+^ T lymphocytes was set as in the upper right dot plot. Representative flow cytometry histograms indicate FFAR2 staining (open purple histograms) and FFAR2 fluorescence minus one control (FMO, filled gray histogram), along with corresponding mean fluorescence intensity (MFI) values, for distinct SILP immune cell subpopulations (delineated as shown in (**A**)) (**B**). Scatter plot represents individual FFAR2 MFI values on designated SILP cell subsets, while horizontal bars indicate means ± SD (*n* = 5) (**C**). The data presented are biological replicates from one experiment.

**Figure EV5:**
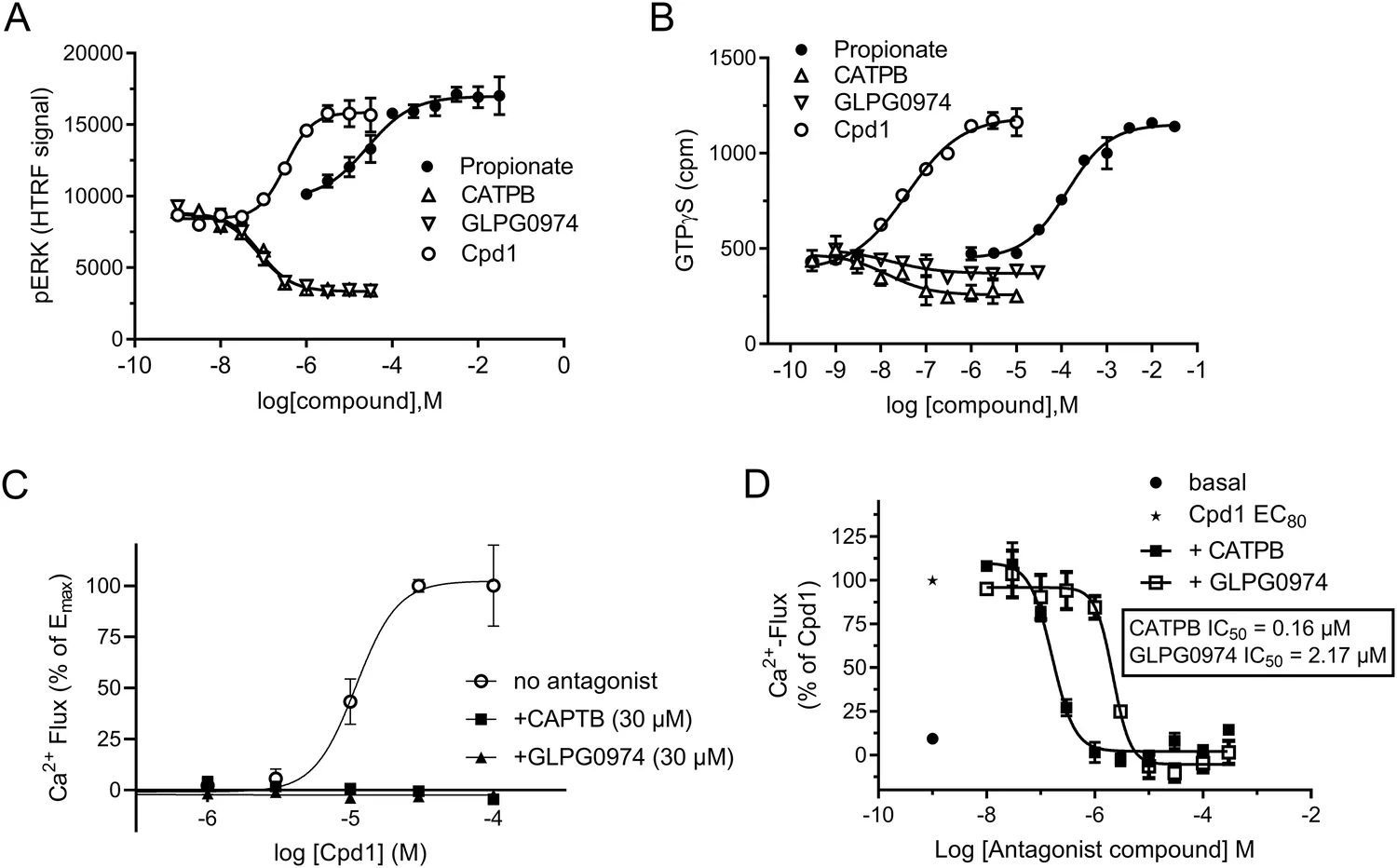
Cpd1 receptor activation and selectivity at FFAR2. Assays conducted on CHO cells stably expressing *h*FFAR2, pERK assay (**A**) and [^35^S]GTPγS assay (**B**) concentration-response curves for the agonists Cpd1 and propionate and the reference antagonists CAPTB and GLPG0974. Cpd1-induced Ca^2+^ flux concentration-response on human polymorphonuclear cells (**C**) is fully blocked by increasing concentrations of the FFAR2 reference antagonists (**D**).

To explore the immunomodulatory effects of Cpd1 in the EAE model, SILP immune cells were isolated from the MOG_35-55_-immunized mice on day 12 post immunization (day four of Cpd1 treatment) and analyzed by flow cytometry (Fig. 2A). Notably, expression of FFAR2 on CD4^+^CD25^+^, Treg, Th1, Th17 cells, and ILC3 was evidenced in the SILP of EAE mice (Fig. EV4A–C). Receptor pharmacology of Cpd1 on CHO cells expressing *h*FFAR2 demonstrated full, potent agonist activity (same E_max_ as propionate but with 100-1000 fold superior potency) in both ERK and [^35^S]GTPγS signaling assays (Fig. EV5A,B). The reference antagonist compounds, CAPTB and GLPG0974, did not demonstrate receptor activation in these assays nor effects on Ca^2+^ flux assays conducted on human polymorphonuclear cells (Fig. EV5C). Cpd1-induced Ca^2+^ flux was completely blocked by both reference FFAR2 antagonists, thereby demonstrating that the pharmacological response to Cpd1 is due to selective activation of FFAR2 (Fig. EV5D).

**Figure EV6:**
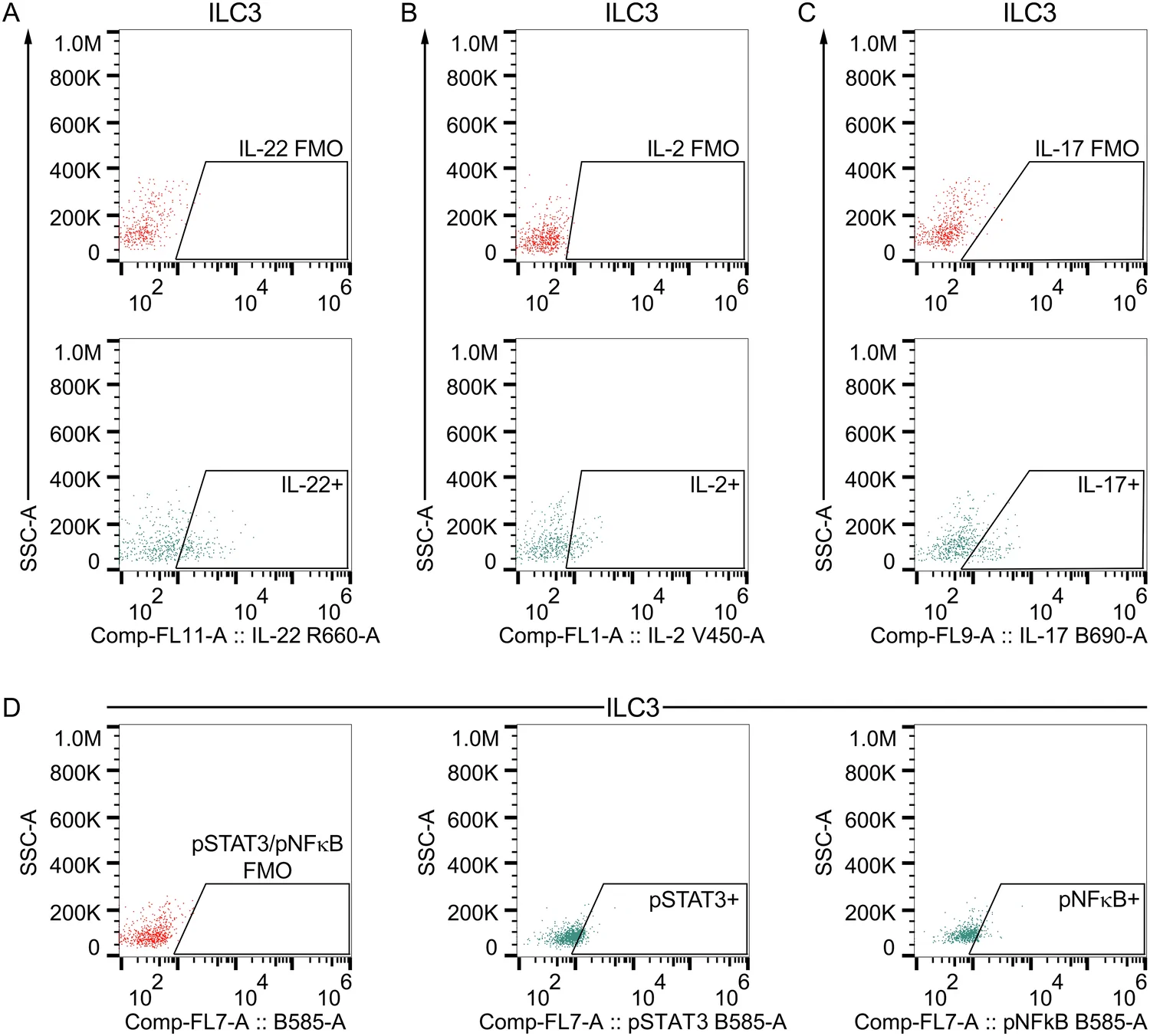
Flow cytometry gating strategies used to analyze SILP ILC3 subsets. Representative flow cytometry dot plots indicate immunostaining of IL-22 (**A**, lower), IL-2 (**B**, lower), IL-17 (**C**, lower), phosphorylated STAT3 (**D**, middle), and phosphorylated NFκB (**D**, right) in SILP ILC3. Gating boundaries for all indicated ILC3 were checked against corresponding fluorescence minus one (FMO) controls (**A**–**C**, upper; **D**, left). SILP ILC3 were gated as illustrated in Fig EV3.

There were more Treg within the SILP CD4^+^ T cells of Cpd1-treated mice, while there was no significant change in the proportions of Th1 and Th17 cells (Fig. 2B–D). As a result, a higher ratio of Treg to both Th1 and Th17 cells was observed in Cpd1-treated EAE mice (Fig. 2E,F). At the same time, the proportion of Treg was not significantly changed under the influence of Cpd1 treatment in cervical lymph nodes (Fig. EV3G), suggesting that the influence of the treatment was not systemic but restricted to the intestinal compartment. Further, there was no effect of Cpd1 on the proportion of IL-22-expressing SILP CD3^+^ cells (2.54 ± 1.18% vs. 2.37 ± 0.89% in EAE and EAE+Cpd1 mice, respectively). In addition, a higher proportion of ILC3, as well as of IL-22-producing ILC3, was observed in the SILP of Cpd1-treated EAE mice (Fig. 2G,J). Cpd1 treatment led to a reduction in the proportion of IL-17-expressing ILC3, while it had no effect on the proportion of IL-2-producing ILC3 (Fig. 2H, I). The proportion of pSTAT3- and pNFκB-expressing ILC3 was also reduced under the influence of Cpd1 (Fig. 2K,L). Gating strategies to examine ILC3 subpopulations are given in Fig. EV6. Furthermore, Cpd1 did not affect the proportion of CD4^+^ T cells, CD11c^+^ cells, B cells, and MHCII^+^ cells, but it reduced the proportion of CD8^+^ T cells, and increased the proportion of CD11b^+^ cells in SILP (Fig. 2M–R). These results indicate that Cpd1 treatment triggered activation of intestinal Treg and ILC3 to ameliorate EAE.

### Cpd1 modulates gut microbiota, but has no effect on intestinal SCFA levels

**Figure EV7:**
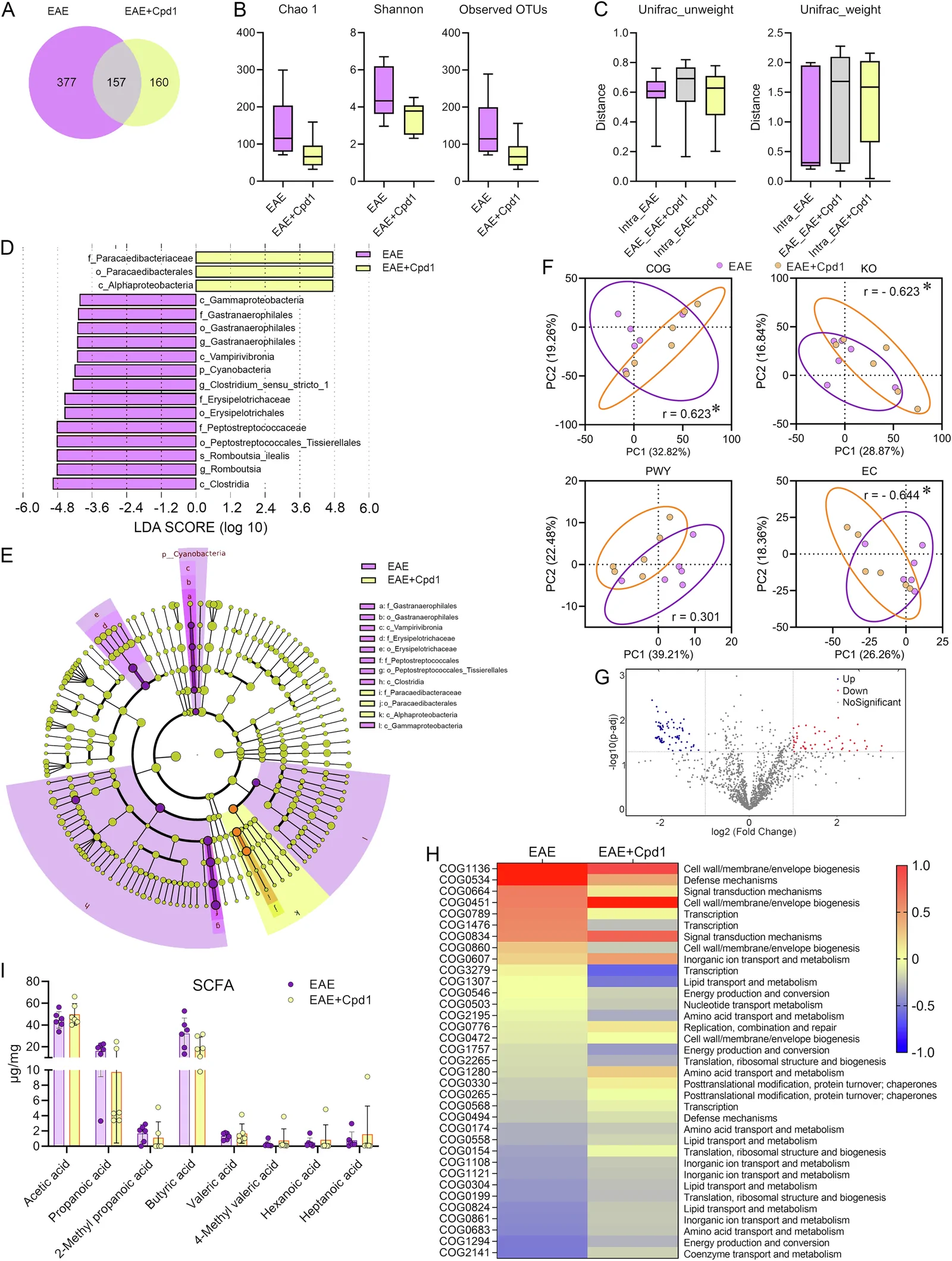
Effect of Cpd1 treatment on gut microbiota and SCFA. EAE mice were treated orally with 20 mg/kg body weight Cpd1 (EAE+Cpd1) or vehicle (EAE) from day 9. On day 12, samples were taken from the terminal ileum (*n* = 5 per group) for 16S rRNA sequencing (**A**–**H**) or from the cecal contents (*n* = 6 per group) for SCFA measurement (**I**). Venn diagram showing the number of exclusive and shared amplicon sequence variants (ASVs) (**A**). Alpha diversity indicators including Chao1 and Shannon indexes, and the number of operational taxonomic units (OTUs) (**B**). Box plots of sample-distance based on unweighted and weighted UniFrac analysis at the ASVs level, the Kruskal–Wallis test showed no statistically significant differences among groups (**C**). Taxonomic groups with LDA scores >4.0 with *P* < 0.05; p, phylum; c, class; o, order; f, family; g, genus; s, species (**D**). The cladogram shows the phylogenetic relationship of the significant ASVs associated with each group (**E**). Violet and yellow indicate taxa enriched in EAE versus EAE+Cpd1 groups, respectively. PC analyses, two-tailed Pearson’s correlation test, *P < 0.05 (**F**). Volcano plot showing upregulated and downregulated functions, │log2fold change│ ≥0 (**G**). COG analyses (**H**). SCFA contents determined by GC/MS (**I**). The data presented are biological replicates from one experiment. Data in (**B**, **C**) are presented as box and whiskers (min to max). Data in (**I**) are presented as mean ± SD (*n* = 6), the Mann–Whitney test showed no statistically significant difference between EAE and EAE+Cpd1 groups.

The response of the intestinal microbiota to oral administration of Cpd1 was also considered a contributing factor to the improvement of clinical signs in EAE. Thus, an exploration of gut microbiota diversity in the terminal ileum was performed on day 12 post immunization (day four of Cpd1 treatment) (Fig. EV7A–E). Results indicate that Cpd1 favored the presence of the *Paracaedibacteraceae* family, the *Paracaedibacterales* order, and the *Alphaproteobacteria* class of bacteria. Further, there were clusters of orthologous genes that were altered by Cpd1 treatment (Fig. EV7F–H). However, levels of SCFA (bacterial digestion products) in the cecal content of EAE mice were not changed under the influence of Cpd1 (Fig. EV7I).

### Cpd1 limits SILP CD4^+^ T-cell activation in EAE

**Figure 3:**
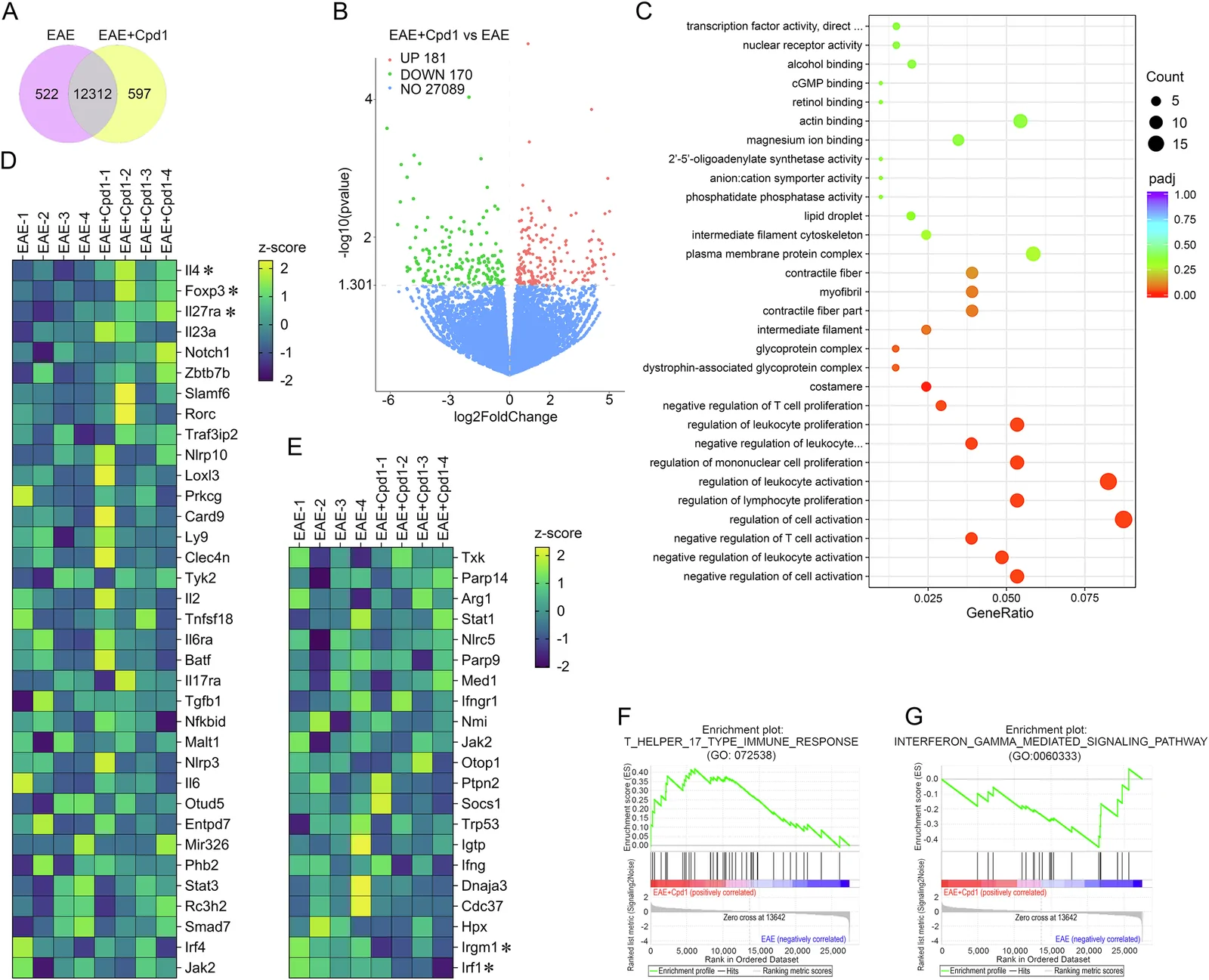
Effect of Cpd1 on SILP CD4^+^ T cells in EAE. EAE mice were treated orally with 20 mg/kg body weight Cpd1 (EAE+Cpd1) or vehicle (EAE) from day 9. On day 12, CD4^+^ T cells purified from SILP immune cells (*n* = 4 per group) were subjected to RNA-seq analysis. Venn diagram showing the number of common and differentially expressed genes (**A**). Volcano plot showing upregulated and downregulated genes, │log2fold change│ ≥0 (**B**). Enriched GO terms: dot plot showing the 30 GO processes with the largest gene ratios (**C**). The size of the dots represents the number of genes in the significantly differentially expressed gene list associated with the GO term, and the color of the dots represents the P-adjusted values. Heatmap (**D**, **E**) showing the expression levels of genes (rows; Z normalized per row) and enrichment plot (**F**, **G**) of Th17-type immune response (**D**, **F**) and IFN-γ-mediated signaling pathways (**E**, **G**). The data presented are biological replicates from one experiment. One-tailed Student’s *t* test. *P < 0.05. Source data are available online for this figure.

Having in mind the importance of CD4^+^ T cells for the pathogenesis of CNS autoimmunity, SILP CD4^+^ T cells were purified, and RNA-seq was performed. More than 12,000 genes overlapped between EAE and EAE+Cpd1 mice (Fig. 3A), with 351 genes reaching a significant difference in expression (Fig. 3B). Various pathways were negatively regulated by Cpd1 in CD4^+^ T cells, including those involved in their proliferation and activation (Fig. 3C). In addition, Cpd1 upregulated genes involved in positive (e.g., *il27ra*) and negative regulation (e.g., *il4* and *foxp3*) of the Th17 response (Fig. 3D,F) and downregulated genes involved in IFN-γ signaling (e.g., *irgm1* and *irf1*) (Fig. 3E,G). Thus, Cpd1 favored the anti-inflammatory profile of SILP CD4^+^ T cells in EAE.

### Cpd1 restrains inflammatory T cell functions in vitro

**Figure 4:**
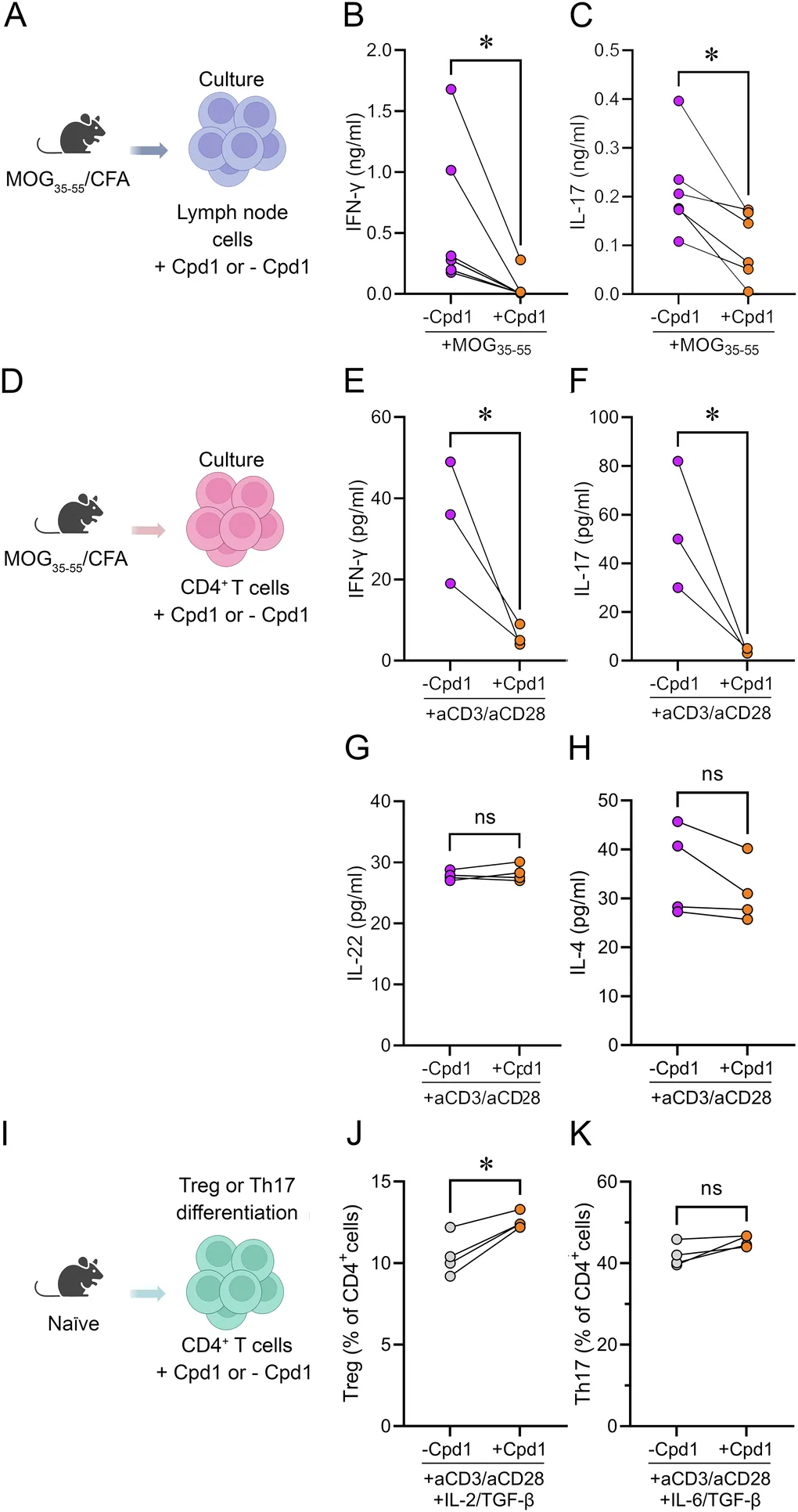
Effects of Cpd1 on mouse T cells in vitro. Inguinal lymph nodes were isolated from EAE mice (days 13 to 17) (**A**). The cells were treated with MOG_35-55_ (10 µg/ml) in the absence or presence of Cpd1 (25 µM), and IFN-γ and IL-17 levels in cell culture supernatants were determined after 24 h of cultivation using ELISA (**B**, **C**). CD4^+^ T cells were sorted from the spleen of EAE mice (days 13–17) (**D**). The cells were treated with Dynabeads Mouse T-Activator CD3/CD28 (8 × 10^5^ beads/ml), in the absence or presence of Cpd1 (25 µM) for 48 h, and the concentrations of IFN-γ, IL-17, IL-22, and IL-4 were determined in the culture supernatants by ELISA (**E**–**H**). Treg and Th17 cells were differentiated from CD4^+^ T cells sorted from mesenteric lymph nodes of non-immunized mice, and cultured in the presence or absence of Cpd1 (20 µM) for 5 days (**I**). The frequency of Treg (CD4^+^CD25^+^FoxP3^+^) and Th17 cells (CD4^+^IL-17^+^) was determined by flow cytometry (**J**, **K**). The data presented are biological replicates from one of three independent experiments. Data are presented as individual values (*n* = 6 in (**B**, **C**), *n* = 3 in (**E**, **F**), *n* = 4 in (**G**, **H**, **J**, **K**)) and analyzed by paired Student’s *t* test *P < 0.05, ns, not significant. Source data are available online for this figure.

**Figure 5:**
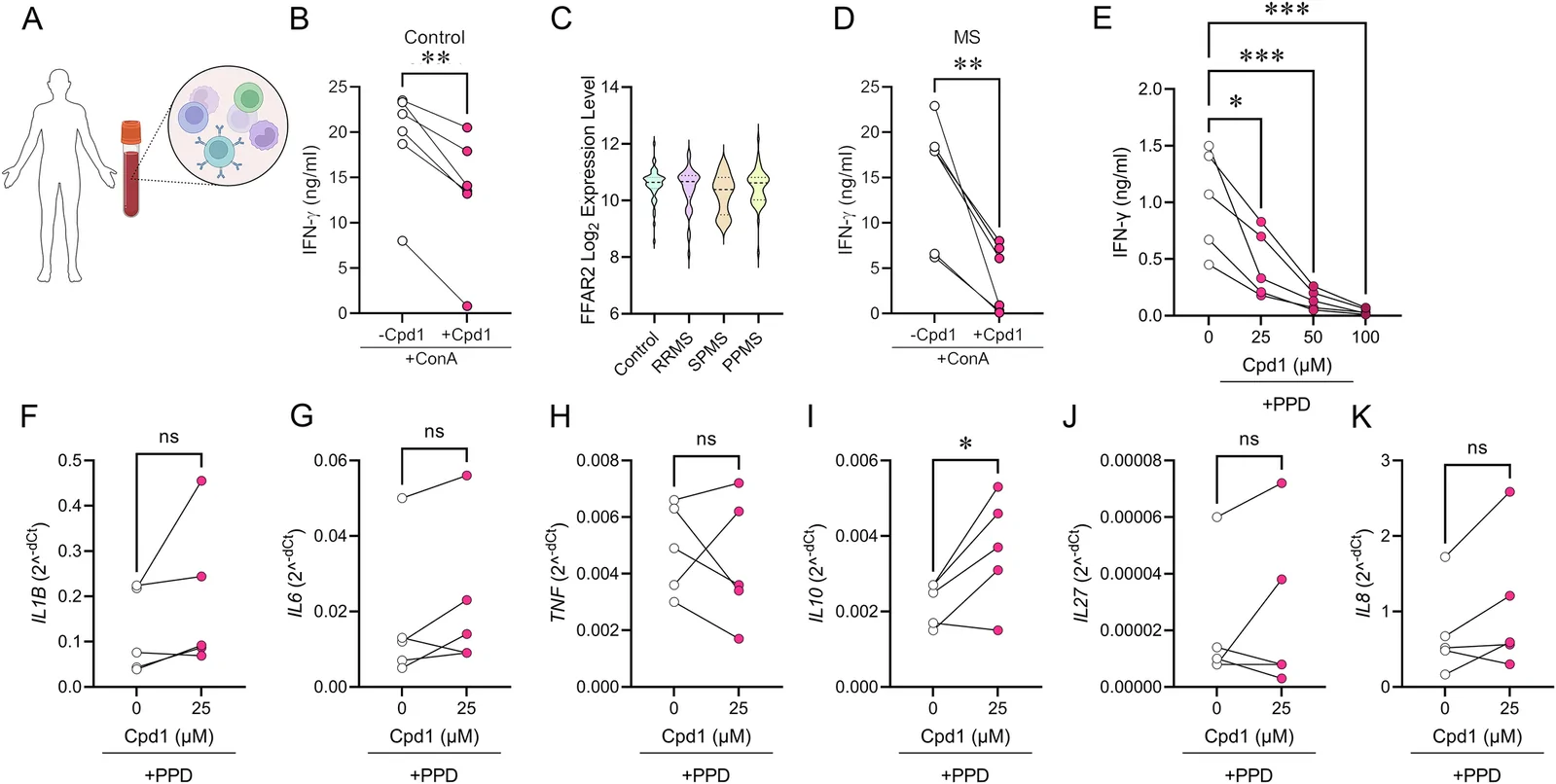
Effects of Cpd1 on human cells in vitro. Human peripheral blood mononuclear cells (PBMC) were obtained from healthy controls and patients with MS (**A**). PBMC from healthy individuals were stimulated for 24 h with ConA (1 µg/ml) in the absence or presence of Cpd1 (25 µM), and the concentration of IFN-γ was determined in the cell culture supernatants by ELISA (**B**). FFAR2 expression in the peripheral blood of healthy controls and MS patients (GSE17048) (**C**). PBMC from individuals with MS were stimulated for 24 h with ConA (1 µg/ml) in the absence or presence of Cpd1 (25 µM), and the concentration of IFN-γ was determined in the cell culture supernatants by ELISA (**D**). PBMC of healthy individuals were stimulated with PPD (10 μg/ml) for 24 h for IFN-γ measurement in the cell culture supernatants by ELISA (**E**) or for 4 h for real-time RT-PCR evaluation of *IL1B* (**F**), *IL6* (**G**), *TNF* (**H**), *IL10* (**I**), *IL27* (**J**), and *IL8* (**K**) expression. The data presented are biological replicates from one experiment. Data are presented as individual values and analyzed by Student’s *t* test (**B**, **D**, **F**–**K**, *n* = 5), or Kruskal–Wallis test (C, control—healthy individuals, *n* = 45; RRMS—relapsing remitting MS, *n* = 36; SPMS—secondary progressive MS, *n* = 20; PPMS—primary progressive MS, *n* = 43), or one-way ANOVA with Dunnett’s test (**E**, *n* = 5), *P < 0.05, **P < 0.01, ***P < 0.001, ns, not significant. Source data are available online for this figure.

CD4^+^ T cells were identified as a major cellular target of Cpd1 in vivo, and this was further explored in vitro. First, we examined the ability of Cpd1 to influence an antigen-specific response in the EAE system. Thus, cells obtained from lymph nodes draining the site of immunization with MOG_35-55_ were treated with the antigen in the presence or absence of Cpd1 (Fig. 4A). MOG_35-55_-stimulated secretion of IFN-γ and IL-17 was reduced in Cpd1-treated cells (Fig. 4B,C). In order to examine if Cpd1 is able to act on CD4^+^ T cells directly, CD4^+^ T cells purified from the spleen of EAE mice (Fig. 4D) were treated with Cpd1, which efficiently reduced the production of IFN-γ and IL-17 (Fig. 4E,F), but not of IL-22 and IL-4 (Fig. 4G,H). Treg differentiation was potentiated with Cpd1, while Cpd1 had no effect on Th17 cell differentiation (Fig. 4I–K). These results showed that CD4^+^ T cells can be directly targeted by Cpd1 in vitro, and further implied that direct effects of Cpd1 on CD4^+^ T cells contribute to the overall immunomodulatory response to Cpd1 observed in vivo. To explore if Cpd1 is able to influence human cells, PBMC were obtained from MS patients and healthy counterparts (Fig. 5A). ConA-induced IFN-γ production from PBMC of healthy subjects was reduced by Cpd1 (Fig. 5B). Analysis of FFAR2 expression in human peripheral blood showed that MS patients do not have impaired expression of this receptor on peripheral blood immune cells (Fig. 5C). Accordingly, Cpd1 reduced IFN-γ production in PBMC obtained from MS patients (Fig. 5D). To explore the effect of Cpd1 in antigen-activated T cells, PBMC of healthy subjects were challenged with PPD and Cpd1 was able to reduce PPD-induced release of IFN-γ (Fig. 5E). At the same time, Cpd1 did not have statistically significant effects on the expression of *IL1B*, *IL6*, *TNF*, *IL8* and *IL27*, while it increased *IL10* expression (Fig. 5F–K), indicating that Cpd1 had an immunomodulatory, rather than an immunosuppressive effect on human immune cells.

### IL-22 plays an important role in EAE amelioration by Cpd1

**Figure 6:**
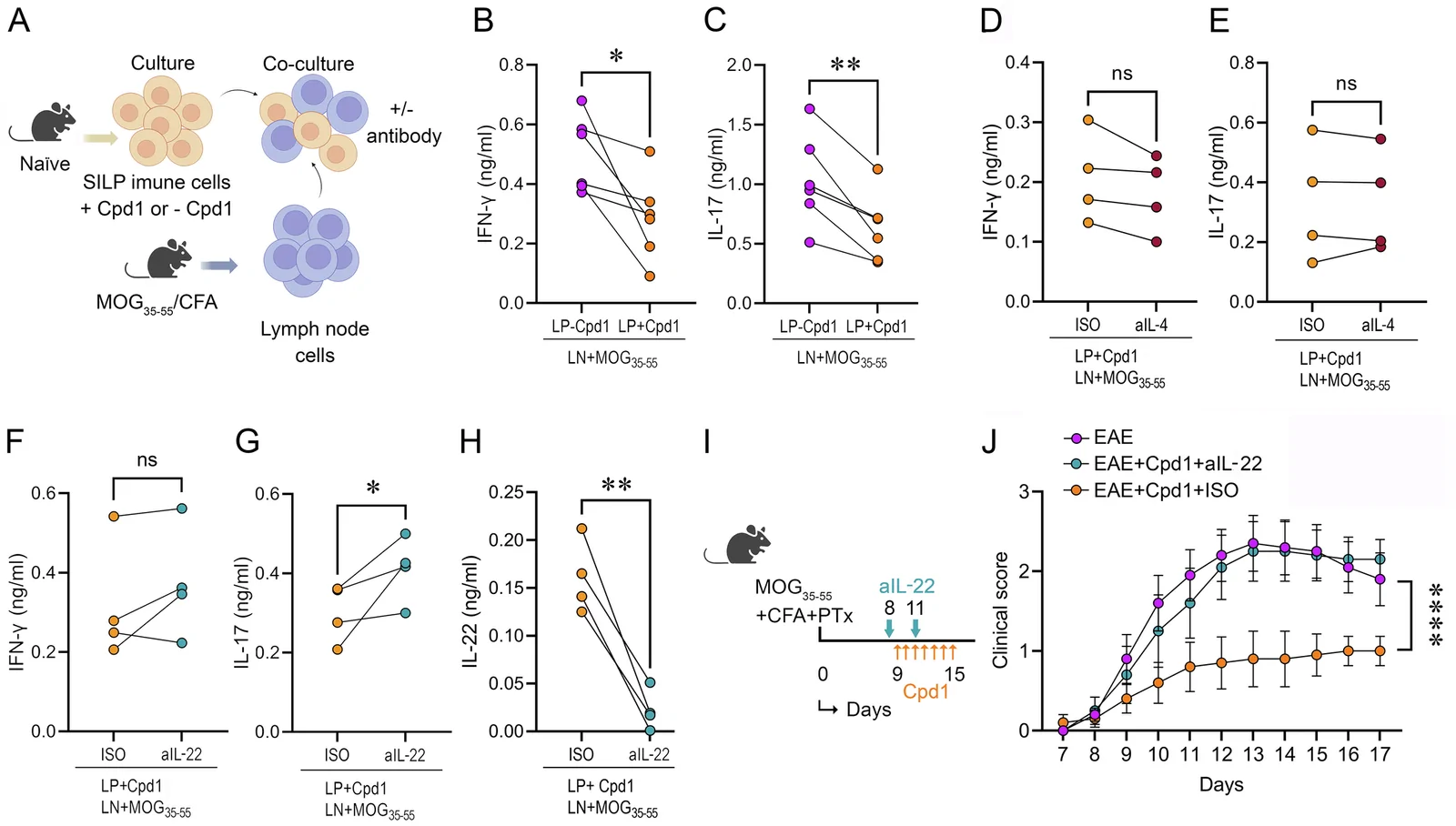
Effect of IL-22 neutralization on Cpd1 activity in vitro and in vivo. Inguinal lymph node cells from EAE mice (day 10) were cultured with SILP cells from non-immunized mice. SILP cells were previously untreated or treated with Cpd1 (25 µM) for 24 h. Co-cultures were performed in the absence or presence of anti-IL-22 antibody (4 µg/ml) or anti-IL-4 antibody (4 µg/ml) or corresponding isotype control (4 µg/ml) for 24 h (**A**). Co-cultured cells were treated with MOG_35-55_ (10 µg/ml) for 24 h, and the concentrations of IFN-γ, IL-17, and IL-22 in the culture supernatants were determined using ELISA (**B**–**H**). The data presented are biological replicates from one of three independent experiments. Data are presented as individual values (*n* = 6 in (**B**, **C**), *n* = 4 in (**D**–**H**)) and analyzed by one-tailed paired Student’s *t* test. EAE mice were treated orally with vehicle (EAE) or 20 mg/kg body weight Cpd1 (days 9–15) and intraperitoneally with anti-IL-22 antibodies (EAE+Cpd1+aIL-22) or isotype control (EAE+Cpd1+ISO) antibodies (both at 50 µg per mouse, days 8 and 11) (**I**). The severity of EAE was assessed by daily clinical scores up to day 17 (**J**). The data presented are biological replicates from one of two independent experiments. Data are presented as mean ± SEM (*n* = 10) and analyzed by two-way ANOVA with Bonferroni’s multiple comparison test. *P < 0.05, **P < 0.01, ****P < 0.0001, ns, not significant. Source data are available online for this figure.

Production of IFN-γ and IL-17 by MOG_35-55_-stimulated lymph node cells was lowered when co-cultured with SILP cells obtained from non-immunized mice and pre-treated in vitro with Cpd1 (Fig. 6A–C). While the addition of anti-IL-4 antibody to the co-culture had no effect on IL-17 or IFN-γ release (Fig. 6D,E), the addition of an anti-IL-22 antibody prevented the Cpd1-imposed limitation of IL-17, but not of IFN-γ release (Fig. 6F,G). The efficiency of IL-22 neutralization was clearly demonstrated by the ability of the anti-IL-22 antibody to annul IL-22 levels in the cell culture supernatants, unlike the isotype antibody, which had no effect on IL-22 levels in the co-cultivation (Fig. 6H). These results showed that SILP cells treated with Cpd1 are able to restrain an antigen-specific T-cell response in an IL-22-dependent way and corroborated our findings from the in vivo experiments that SILP immune cells increase their regulatory activity in response to Cpd1. To explore if IL-22 produced under the influence of Cpd1 contributes to EAE amelioration, neutralizing antibodies against IL-22 were administered in parallel with Cpd1 in EAE (Fig. 6I). The anti-IL-22 antibodies counteracted the beneficial effects of Cpd1 in EAE (Fig. 6J), demonstrating the importance of IL-22 for Cpd1-imposed reduction of EAE.

## Discussion

Oral administration of Cpd1, a specific agonist of FFAR2, efficiently ameliorates CNS autoimmunity in experimental animals. The FFAR2-mediated modulation of autoimmune activity is achieved by enhancing the regulatory functions of ILC3 and Treg within the immune environment of the small intestine and by inhibition of immune cell infiltration and activity in the CNS. IL-22 is identified as an important mediator for the amelioration of the CNS autoimmunity by Cpd1.

Our results imply that the previously reported response to SCFA in EAE mice (Chitrala et al, 2017; Haghikia et al, 2016; Mizuno et al, 2017) could have been specifically mediated by FFAR2 based on the robust response to the selective ligand, Cpd1 (Chun et al, 2019). This view is corroborated by a study in a mouse colitis model that directly compared the effects of various SCFA types and Cpd1, showing that the effect of Cpd1 on ILC3 proliferation and corresponding IL-22 production was more robust than that of SCFA (Chun et al, 2019), and furthermore, that the response to Cpd1 is absent in FFAR2-knockout mice, suggesting target selectivity.

There is an unmet need for novel therapeutics for autoimmune diseases, including MS. Cpd1 was previously shown to ameliorate EAE when applied in the inductive phase of the disease (Lazarević et al, 2024), and here we show that it is equally efficient in the effector phase of EAE. This is important for its potential translation to therapeutic use in humans. Indeed, the vast majority of MS patients are diagnosed only after the clinical signs of the disease are evident. Thus, potential drugs need to counteract already ongoing CNS autoimmunity. The ability of Cpd1 to act against the established inflammatory process in the CNS in EAE suggests that it could be efficient as an MS therapeutic.

Our interest in Cpd1 as a candidate for the treatment of autoimmune diseases stems from a previous report demonstrating that pharmacological activation of FFAR2 stimulates the proliferation and function of intestinal ILC3 and promotes their protective role in gut homeostasis and pathogen defense (Chun et al, 2019). These actions of ILC3 may also be relevant to CNS autoimmunity, as the loss of intestinal barrier integrity and the consequent immune imbalance are associated with the onset of EAE in mice (Nouri et al, 2014). Furthermore, it has been shown that the small intestine immune compartment is important for the initiation and regulation of the autoimmune response in distant organs, such as the spinal cord and brain in EAE (Miyauchi et al, 2020; Ivanov et al, 2008). Therefore, a pharmacological intervention targeting the intestinal immune cells may be a viable approach for the treatment of MS. So far, there have been no direct attempts to treat MS or any other autoimmune disease through the modulation of gut immune cells, although there are reports on the effects of established MS drugs on the gut immune system. For example, Fingolimod was reported to influence gut ILC3 in mice (Eken et al, 2019) while Teriflunomide was shown to induce gut Treg in EAE (Ochoa-Repáraz et al, 2016).

ILC3 efficiently control effector Th1 and Th17 cells and push T effector/Treg balance towards the regulatory axis (Hepworth et al, 2013; Klose and Artis, 2020; Zhou et al, 2019). The importance of IL-22 for the observed beneficial effects of Cpd1 in EAE is clearly demonstrated by the annulment of these effects by the administration of neutralizing anti-IL-22 antibodies. In response to Cpd1 treatment, an increased proportion of ILC3 producing IL-22 and a reduced proportion of IL-17-producing ILC3 were observed in EAE. Potentiation of ILC3 immunoregulatory properties by Cpd1 affected the Th cell distribution in the SILP, as treated animals demonstrated an increased ratio of Treg to both Th1 and Th17 cells. This observation is complemented by our in vitro findings, which demonstrate that Cpd1-treated SILP cells use IL-22 to restrict antigen-specific Th17 cells. In the same setting, IL-4 blockade did not restore antigen-stimulated IL-17 release, thus excluding IL-4 as a mediator of Cpd1-induced effects in our system. In addition to ILC3, intestinal T cells are also able to produce ample amounts of IL-22. Still, the absence of Cpd1 effect on IL-22 production/secretion in CD4^+^ T cells in vitro implies that IL-22 production by CD4^+^ T cells is not the primary mode of action of Cpd1 in EAE. Furthermore, it is known that immune cell populations besides ILC3 and CD4^+^ T cells produce IL-22 (Keir et al, 2020), and the effect of Cpd1 on IL-22 release from these cells in EAE cannot be excluded. A view that stimulation of IL-22 production by ILC3 might be the major effect of Cpd1 is supported by a report demonstrating that FFAR2-mediated potentiation of IL-22 production by intestinal ILC3 is crucial to ameliorate *Clostridium difficile* infection (Fachi et al, 2020).

In EAE mice, Cpd1 treatment is proposed to influence Treg through ILC3 in two conceivable ways: through the production of IL-2, a mandatory growth factor for Treg (Zhou et al, 2019), or through the presentation of antigens within MHC class II molecules to T cells, but without the required costimulatory signals (Lyu et al, 2022; Hepworth et al, 2013). Our current findings show that Cpd1 was ineffective in driving IL-2^+^ ILC3 in EAE mice, whereas we have previously shown that Cpd1 applied in the inductive phase of EAE efficiently lowers CD40 expression on MHCII-expressing SILP ILC3 in rats (Lazarević et al, 2024). These data support the hypothesis that the Cpd1-induced increase in the proportions of Treg is a consequence of the stimulatory effect of ILC3 on Treg in the SILP.

Cpd1 treatment also appears to directly influence Treg and Th17 cell populations. Expression of FFAR2 is evident on CD4^+^ T cells, including Treg and Th17 cells, in the SILP of EAE mice. Cpd1 restrained the T cell antigen-specific response and affected the capacity of purified CD4^+^ T cells to produce inflammatory cytokines. Although our RNA sequencing analysis showed increased expression of IL-4 in intestinal CD4^+^ T cells, Cpd1 did not increase IL-4 release from purified CD4^+^ T cells in vitro. Our findings are complemented by a report that the adoptive transfer of colonic intraepithelial T cells treated in vitro with another FFAR2 agonist (4-CMTB) ameliorated EAE (Prado et al, 2023). However, despite FFAR2 expression on Th17 cells, in vitro differentiation of Th17 cells was not influenced by Cpd1. On the contrary, Cpd1 promoted Treg differentiation from purified CD4^+^ T cells in vitro. Our ex vivo findings suggest that Cpd1 increased the proportion of Treg in the SILP, but did not alter the proportion of Th17 cells. These data are consistent with a previous report of the expression and role of FFAR2 on colonic Treg (Smith et al, 2013). Altogether, our study identifies both ILC3 and Treg as direct cellular targets of FFAR2 agonism. Collectively, these data suggest that the observed amelioration of CNS autoimmunity symptoms occurs due to the activation of the intestinal ILC3-Treg axis.

Alterations in gut microbiota composition paralleled the disease-ameliorating effects of Cpd1 in EAE mice in this study. We recently demonstrated the effects of Cpd1 on the gut microbiome in EAE rats (Jevtić et al, 2025). However, we could not identify a similar pattern of Cpd1-induced microbiota alterations between the two species. The observed changes in microbiota species in Cpd1-treated EAE mice (in particular, of the Paracaedibacteraceae family, the Paracaedibacterales order, and the Alphaproteobacteria class of bacteria) may be interpreted as a direct influence of Cpd1 on gut bacteria or as an indirect outcome mediated by changes in intestinal immune cell composition/activity in response to Cpd1. As there are no reports of FFAR2 expression by bacteria, the latter explanation seems reasonable. Cpd1 did not affect ileal SCFA content, indicating that the effects of Cpd1 on intestinal microbiota have a negligible contribution to its beneficial action in EAE.

Communication between the intestinal immune system and target organs in autoimmune disorders is evident. Concerning EAE, it has been shown that the cells with encephalitogenic potential are additionally activated towards a pathogenic phenotype, or modulated to convert into regulatory cells within the gut, followed by their migration into the CNS (Miyauchi et al, 2020; Esplugues et al, 2011; Lee et al, 2011; Berer et al, 2014). Our results imply that Cpd1 contributes to the immunoregulatory milieu in the small intestine and limits the encephalitogenic properties of CD4^+^ T cells. However, migration of immune cells from the SILP to target organs and lymphoid organs has not been studied here. Thus, migration studies are required to explore if T cells and/or ILC3 migrate from the small intestine into organs of interest, such as the spinal cord, and lymphoid organs.

A limitation of this study is that it focuses on the small intestine and does not consider possible effects of Cpd1 on colonic immune cells in EAE. The decision to limit our study to SILP cells stems from previous reports showing the importance of the small intestine in the pathogenesis of autoimmunity, particularly regarding autoreactive Th17 cells (*vide supra*). As Cpd1 was previously shown to stimulate the regulatory properties of ILC3 in the colon (Chun et al, 2019), the observed effects of Cpd1 in EAE could also be mediated by regulatory immune cells in the colon.

**Figure 7:**
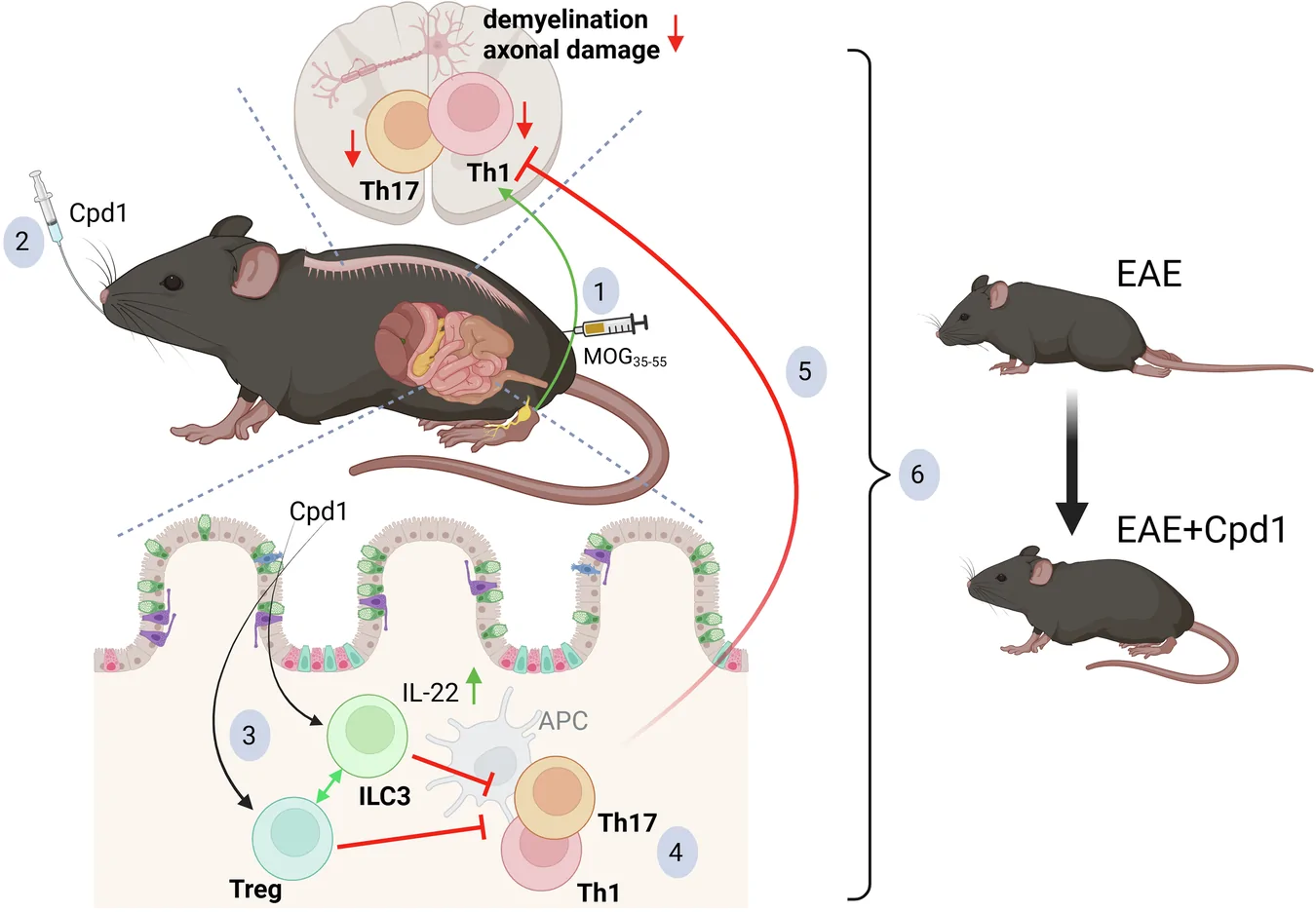
Cpd1 fends against CNS autoimmunity. Encephalitogenic T cells emerge from lymph nodes draining the site of immunization (1). Oral administration of Cpd1 (2) leads to expansion of ILC3 and Treg in the intestinal lamina propria, and increased expression of IL-22 by ILC3 (3). Downregulation of Th1 and Th17 cells also occurs in the intestine (4). Overall activity of Cpd1 leads to inhibition of CNS inflammation and degeneration (5) and EAE (6).

In conclusion, we demonstrated that Cpd1 administered orally in the effector phase of EAE efficiently ameliorates the disease (Fig. 7) to extend our previous findings about Cpd1 administered in the inductive phase of EAE (Lazarević et al, 2024). In addition, it is demonstrated that human immune cells originating from peripheral blood are also responsive to Cpd1. Importantly, peripheral blood cells of MS patients do not have impaired expression of FFAR2 in comparison to healthy individuals. Accordingly, Cpd1 reduces the capacity of peripheral blood cells collected from MS patients to produce a key inflammatory cytokine IFN-γ as a potential translational marker that could be used in further clinical investigation.

Autoimmune diseases are an intriguing consequence of the inadequate functioning of the immune system and evidence of its imperfection at the level of the individual. While the initiation of most autoimmune diseases remains elusive, the ever-growing knowledge of the pathogenic mechanisms involved in the execution of the autoimmune response and the regulation of this process has drawn attention to the possible pathogenesis of disease in the gut and the associated involvement of the microbiota. Our study demonstrated a regulatory role for SILP immune cells, particularly Treg and ILC3, in CNS-directed autoimmunity. Furthermore, we showed that FFAR2 agonism is a viable pathway for the treatment of a CNS autoimmune disease. Accordingly, further studies on the use of specific FFAR2 agonists in the therapy of MS are warranted.

## Methods

**Table:** Reagents and tools table

Reagent/resource	Reference or source	Identifier or catalog number
**Experimental models**		
C57BL/6 mice	Charles Rivers	C57BL/6NCrl
Dark Agouti rats	Badovinac et al, J Neuroimmunol. 1998 May 1;85(1):87–95.	/
CHO-K1	ATCC	CCL-61
**Recombinant DNA**		
**Antibodies**		
CD25	Thermo Fisher Scientific	417-0251-82, 12-0251-82, 35-0251-82
CD3	Biolegend	100222
CD4	Thermo Fisher Scientific	69-0042-82, 416-0042-82
CD4	Thermo Fisher Scientific	48-0041-80, 11-0041-85, 25-0041-82
CD45	Thermo Fisher Scientific	69-0451-82, 17-0451-82, 25-0451-82
FFAR2	Thermo Fisher Scientific	FFAR2-1-FITC
FoxP3	Thermo Fisher Scientific	12-5773-82, 17-5773-82, 45-5773-82
Horse-anti-mouse IgG	Vectastain	PK-4002
IFN-γ	Thermo Fisher Scientific	48-7311-82, 45-7311-82, 17-7311-82
IL-10	Thermo Fisher Scientific	12-7101-82, 45-7101-82
IL-17	Thermo Fisher Scientific	11-7177-81, 12-7177-81, 45-7177-81
IL-2	Thermo Fisher Scientific	48-7021-82, 25-7021-80
IL-22	Thermo Fisher Scientific	12-7221-82
IL-22	Thermo Fisher Scientific	17-7222-82, 16-7222-82
IL-4	Thermo Fisher Scientific	14-7041-81
Neurofilament H/SMI32	Biolegend	801707
pNFκB p65	Thermo Fisher Scientific	MA537165
pSTAT3	Thermo Fisher Scientific	12-9033-42
Rat IgG2a kappa Isotype Control	Thermo Fisher Scientific	16-4321-82
RORγt	Thermo Fisher Scientific	12-6988-82, 46-6988-80, 17-6988-82
T-bet	Thermo Fisher Scientific	50-5825-82, 45-5825-80
**Oligonucleotides and other sequence-based reagents**		
*ACTB*	Metabion	CAT GTA CGT TGC TAT CCA GGC
*ACTB*	Metabion	CTC CTT AAT GTC ACG CAC GAT
*IL1B*	Metabion	ACA GAT GAA GTG CTC CTT CCA
*IL1B*	Metabion	GTC GGA GAT TCG TAG CTG GAT
*IL6*	Metabion	AGT CCT GAT CCA GTT CCT GC
*IL6*	Metabion	CTA CAT TTG CCG AAG AGC CC
*IL27*	Metabion	CAA GCT GGT GTC TGG GGA TT
*IL27*	Metabion	GGA AAC ATC AGG GAG CTG CT
*IL8*	Metabion	CAG TTT TGC CAA GGA GTG CT
*IL8*	Metabion	ACT TCT CCA CAA CCC TCT GC
*IL10*	Metabion	AGA CAT CAA GGC GCA TGT GA
*IL10*	Metabion	AGG CAT TCT TCA CCT GCT CC
*TNF*	Metabion	AGG ACC AGC TAA GAG GGA GA C
*TNF*	Metabion	CCC GGA TCA TGC TTT CAG TG
**Chemicals, enzymes, and other reagents**		
[^35^S]GTPγS	Perkin Elmer	NEG030X
2-mercaptoethanol	Sigma-Aldrich	M3148
Concanavilin A	Pharmacia	17-0450-01
Cpd1	Epics Therapeutics S.A.	/
DuoSet ELISA Mouse IFN-γ,	R&D Systems	DY485
DuoSet ELISA Mouse IL-17	R&D Systems	DY421
DuoSet ELISA Mouse IL-22	R&D Systems	DY582
Dynabeads Mouse T-Activator CD3/CD28	Thermo Fisher Scientific	11452D
eBioscience™ Cell Stimulation Cocktail	Thermo Fisher Scientific	00-4970-93
ExtrAvidin®-Peroxidase	Sigma-Aldrich	E2886
FBS	Capricorn Scientific	FBS-12A
Ficoll-Hypaque	Amersham Biosciences	17-1440-02
Fluo-4-AM	Molecular probes	14201
HBSS	BioWhitaker	BE10-527F
HTRF pERK™ kit	Cisbio	64ERKPEG
Human IFN-γ ELISA Kit	Thermo Fisher Scientific	KHC4021
Incomplete Freund’s adjuvant	BD Bioscience	263910
LFB solution	abcr Gmbh & Co	/
LPS O55:B5	Sigma-Aldrich	L2880
Lymphocyte separation medium	Capricorn Scientific	LSN-A
*M. tuberculosis H37Ra*	BD Bioscience	231141
MOG_35-55_	SB-PEPTIDE	SB023
Mouse recombinant IL-2	Thermo Fisher Scientific	212-12
Mouse recombinant IL-6	Thermo Fisher Scientific	216-16
Mouse recombinant TGF-β	Thermo Fisher Scientific	14-8342
Mouse Regulatory T cell Staining Kit	Thermo Fisher Scientific	88-8111-40
Nutrient Ham’s F-12 medium	Thermo Fisher Scientific	11765054
Pertussis toxin	Biotechne	3097
PFA	Thermo Fisher Scientific	169650010
Pierce BCA Protein Assays Kits	Thermo Fisher Scientific	23225
pluronic acid	Molecular Probes	6867
PPD	Torlak	/
PVT-WGA beads	Perkin Elmer	RPNQ0001
Quick-RNA MicroPrep	Zymo Research	R1050
RPMI 1640	Capricorn Scientific	RPMI-HA
Sodium pyruvate	Capricorn Scientific	NPY-B
streptavidin-APC-Cy7	Biolegend	405208
TRIzol	Thermo Fisher Scientific	15596026
ZymoBIOMICS DNA Miniprep Kit	Zymo Research	D4300
**Software**		
ClusterProfiler (Version 3.8.1)		Guangchuang et al, OMICS: A Journal of Integrative Biology, 2012, 16(5):284–287.
CytExpert software v2.4	Beckman Coulter	
DESeq2 program (Version 1.20.0)		Love et al, Genome Biology, 15:550.
FlowJo V10.8.1 Software	BD Bioscience	
GraphPad Prism 9 software	GraphPad Software	
gsea software (Version 4.3.2)		Subramanian et al, Proceedings of the National Academy of Sciences, 102(43), 15545–15550.
HISAT2 (Version 2.0.5)		Kim D et al, Nat Biotechnol. 2019 Aug;37(8):907–915. https://doi.org/10.1038/s41587-019-0201-4. E-pub 2019 Aug 2. PMID: 31375807; PMCID: PMC7605509.
QuantStudioTM Design&Analysis Software v1.5.2	Applied Biosystems	
QuPath software		Bankhead, P et al QuPath: Open source software for digital pathology image analysis. Scientific Reports (2017). https://doi.org/10.1038/s41598-017-17204-5
R version 4.4.2		R Core Team (2021). R: A language and environment for statistical computing. R Foundation for Statistical Computing, Vienna, Austria. URL https://www.R-project.org/.
Sample size calculator	University of Vienna	https://homepage.univie.ac.at/robin.ristl/samplesize.php
**Other**		
384-well plates	Greiner	784080
96-well Optiplate	Perkin Elmer	6005299
EDTA-coated vacutainer tubes	Neomedica	/
Heparin tubes	BD Bioscience	368480
Superfrost Plus microscope slides	Thermo Fisher Scientific	22037246
U-bottom 96-well plate	Sarstedt	83.3925

### Experimental animals

C57BL/6 mice and Dark Agouti rats were bred and maintained in the animal facility of the Institute for Biological Research “Siniša Stanković”. Animals were held under a conventional holding environment, with free access to food and water. The number of animals per cage was as follows: 3–5 rats, 5–8 mice. Animals were age and sex matched and randomly assigned to the experimental groups. Blinded outcome assessment was not performed. Permissions for the experiments were granted by the Veterinary Administration of the Ministry of Agriculture, Forestry and Water Management, Republic of Serbia (No 323-07-05815/2020-05/1, 323-07-10741/2022-05, 323-07-04423/2023-05).

### EAE in mice

Female C57BL/6 mice (10-12 weeks old) were immunized with myelin oligodendrocyte glycoprotein (MOG)_35-55_ (SB-PEPTIDE, Saint-Egrève, France), as previously described (Bjedov et al, 2024). Complete Freund’s adjuvant (CFA) was obtained by supplementing incomplete Freund’s adjuvant with *M. tuberculosis* (both from BD Bioscience, Franklin Lakes, NJ). Pertussis toxin (PT) was from Biotechne, Minneapolis, MN. Cpd1, compound 1 in patent no. WO 2011/073376 A1, provided by Epics Therapeutics S.A. under Material Transfer Agreement permitting use of the compound (Forbes et al, 2015; Hoveyda et al, 2011), was administered orally through a cannula (20 mg/kg body weight in 100 µl, daily), from day 9 to day 15 post-immunization. Control mice received an equal volume of vehicle (water). Mice were observed daily for clinical scoring: 0—no motor dysfunctions, 0.5—limp tip of tail, 1—limp tail, 1.5—limp tail and slightly wobbly walking, 2—wobbly walking, hind legs are not spread apart when picked up by the base of the tail, 2.5—dragging of both hind legs, 3—complete paralysis of one hind leg, or hind leg paddling, 3.5—complete paralysis of both hind legs, 4—complete paralysis of hind legs and partial front leg paralysis, 4.5—as 4, no movement around the cage, 5—moribund state or death.

### EAE in rats

Six-month-old female Dark Agouti rats were immunized with spinal cord homogenate without CFA, as previously described (Lazarević et al, 2024), and treated orally through a cannula with Cpd1 (15 mg/kg in 200 µl, once per day), 7–15 days post-immunization. Control rats received an equal volume of water. Rats were examined daily for clinical scoring, as described previously (Lazarević et al, 2021).

### Anti-IL-22 treatment

EAE mice were treated orally with vehicle or 20 mg/kg body weight Cpd1 from day 9 to day 15 post-immunization and intraperitoneally with neutralizing anti-IL-22 antibodies (rat monoclonal IL22JOP), or isotype control antibodies (both from Thermo Fisher Scientific, Waltham, MA), both at 50 µg per mouse, on days 8 and 11 post-immunization.

### Human samples

Peripheral blood samples were obtained from MS patients and matched healthy individuals (Table 1). All procedures related to the use of human blood are in conformity with the recommendations provided in the Code of Ethics of the World Medical Association (Declaration of Helsinki) and the principles set out in the Department of Health and Human Services Belmont Report. Informed consent was obtained from all human subjects. Publicly available gene expression dataset GSE17048 was retrieved from the Gene Expression Omnibus (GEO) database (National Center for Biotechnology Information. Gene Expression Omnibus: GSE17048. National Library of Medicine, Bethesda, MD, USA. Available from: https://www.ncbi.nlm.nih.gov/geo/query/acc.cgi?acc=GSE17048). GSE17048 comprises whole blood transcriptomic profiles from 99 multiple sclerosis patients and 45 healthy controls, generated using the Illumina HumanHT-12 V3.0 microarray platform. Raw expression data were processed using R version 4.4.2. Differential expression analysis was performed using the limma package, employing linear modeling with empirical Bayes moderation. Probe-to-gene symbol mapping was performed using the platform-specific package, IlluminaHumanv3.db (version 1.26.0). For probes mapping to FFAR2, log2 fold changes were extracted.

**Table 1:** Age and sex of MS patients and healthy controls.

MS patients		Healthy controls	
Age	Sex	Age	Sex
64	M	62	M
53	F	52	F
51	F	51	F
45	F	46	F
45	F	44	F
43	F	43	F
39	F	40	F
34	M	36	M
31	M	29	M
27	M	27	M

*F* female, *M* male.

### Cell isolation and culture

Inguinal and mesenteric lymph nodes, spleen, SILP, and spinal cord-infiltrating cells were obtained as described previously (Lazarević et al, 2024; Bjedov et al, 2024; Stanisavljević et al, 2017; Stegnjaić et al, 2024). CD4^+^ T cells were purified from SILP, splenic, or mesenteric lymph node cell suspensions by sorting on BD FACSAria™ III Cell Sorter (BD Biosciences), as described previously (Bjedov et al, 2024). All cultivations were performed in RPMI 1640 medium (Capricorn Scientific, Ebsdorfergrund, Germany) supplemented with various concentrations of fetal bovine serum (FBS, Capricorn Scientific) or autologous mouse serum, at 37 °C in a humidified incubator in a 5% CO_2_ atmosphere, as previously described (Bjedov et al, 2024; Stegnjaić et al, 2024).

SILP immune cells obtained from non-immunized mice were stimulated with lipopolysaccharide (LPS, Sigma-Aldrich, St. Louis, MI, 10 ng/ml). Spleen CD4^+^ T cells were obtained from MOG_35-55_-immunized mice on day 9 after immunization and treated with Dynabeads Mouse T-Activator CD3/CD28 (8×10^5^ beads/ml, Thermo Fisher Scientific), in the absence or presence of Cpd1 (25 µM) for 48 h.

Human peripheral blood mononuclear cells (PBMC) were isolated from fresh blood samples obtained from MS patients and healthy individuals. The blood samples were collected in EDTA-coated vacutainer tubes (Neomedica, Niš, Serbia), and 4–6 h later the samples were diluted two times in phosphate buffer saline (PBS), overlaid on lymphocyte separation medium (Capricorn Scientific), and centrifuged at 720×* g* for 20 min. Afterward, PBMC were collected from the interlayer ring and stimulated with 1 µg/ml of concanavalin A (ConA, Pharmacia, Uppsala, Sweden) or 10 µg/ml purified protein derivative (PPD, Torlak, Belgrade, Serbia) in the absence or presence of Cpd1.

### Treg and Th17 differentiation

CD4^+^ T cells sorted from mesenteric lymph nodes of non-immunized mice were seeded in a U-bottom 96-well plate (Sarstedt, Numbrecht, Germany), 5 × 10^5^ cells/well in 200 µl of RPMI 1640 supplemented with 10% FBS, 5 µM 2-mercaptoethanol (Sigma-Aldrich), and 10 nM sodium pyruvate (Capricorn Scientific). CD4^+^ T cells were cultivated in the presence of Dynabeads Mouse T-Activator CD3/CD28 (8 × 10^5^ beads/ml). Mouse recombinant transforming growth factor (TGF)-β (2 ng/ml) and mouse recombinant IL-2 (10 ng/ml) (both from Thermo Fisher Scientific) were used for Treg differentiation. Mouse recombinant TGF-β (10 ng/ml) and mouse recombinant IL-6 (10 ng/ml, Thermo Fisher Scientific) were used for Th17 differentiation. Cultivation lasted for 120 h in the absence or presence of Cpd1 (20 µM).

### Co-cultivation of lymph node cells and SILP cells

SILP cells were obtained from non-immunized C57BL/6 mice and cultivated in the absence or presence of Cpd1 (25 µM) for 24 h. Subsequently, the cells were washed, and 1.2 × 10^5^ SILP cells were co-cultivated with inguinal lymph node cells (6 × 10^5^), which were obtained from EAE-immunized C57BL/6 mice on day 10 in the presence of 4 µg/ml of neutralizing anti-IL-22 or neutralizing anti-IL-4 (rat monoclonal 11B11, Thermo Fisher Scientific), or corresponding isotype control. Co-cultivation lasted for 24 h and took place in U-bottom 96-well plates (Sarstedt).

### Histology

Mice were euthanized through CO_2_ inhalation and transcardially perfused with ice-cold PBS and fixed in 4% paraformaldehyde (PFA, Thermo Fisher Scientific). After dissection of the spinal cords, the tissue was incubated in 4% PFA overnight at 4 °C. Tissue dehydration was performed in graded alcohols. Coronal lumbar and cervical sections of the spinal cord were dissected and embedded in paraffin.

Hematoxylin and eosin staining was used to detect inflammatory infiltrates in spinal cord tissue sections from 3 EAE mice (day 17 post immunization) per group (Lazarević et al, 2021). Five-micrometer transversal lumbosacral sections (L1–L5) were cut, and four successive slices were placed on the same glass slide. Infiltrates were defined as clusters of mononuclear cells localized in the perivascular space and the subpial region of the spinal cord. The number of infiltrates and cells per infiltrate was counted from four spinal cord tissue sections per animal.

For Luxol fast blue/Periodic acid-Schiff stain (LFB/PAS) and immunostaining, tissue sections (3 μm thick) were cut on a microtome and transferred to Superfrost Plus microscope slides (Thermo Fisher Scientific). Deparaffination was followed by hydration through graded alcohols, and LFB/PAS or immunohistochemistry stainings were performed.

For LFB, the samples were rehydrated incompletely (stopped after 96% EtOH) and incubated overnight at 56 °C in 0.1% LFB solution (abcr Gmbh & Co, Karlsruhe, Germany, diluted with 96% EtOH/0.05% acetic acid). Then, they were washed in 96% EtOH and incubated in 0.1% lithium carbonate (Li_2_CO_3_) for 30 s. The samples were washed very briefly in 70% EtOH and distilled water before incubation for 10 min in 0.8% periodic acid (H_5_IO_6_) to wash off excess Li_2_CO_3_. After washing with distilled water, the samples were incubated in Schiff reagent for 20 min. Excess reagent was washed off with sulfite washing solution (0.1% of concentrated (37%) HCl, 4% potassium metabisulfite in H_2_O). Prior to the final dehydration and embedding, the samples were washed extensively in non-distilled water for 10 min.

For immunostaining, paraffin-embedded sections were completely rehydrated in boiling 0.1 M citric acid (pH 6.0) for 20 min in a microwave oven. After cooling down to room temperature, the samples were washed in distilled water and PBS, followed by incubation in 3% hydrogen peroxide (H_2_O_2_) in PBS for 10 min. The sections were then blocked by incubation in 10% FCS/0.3% Tween in PBS for 30 min. Primary nonphosphorylated anti-neurofilament H/SMI32 antibody (clone SMI32, Biolegend, San Diego, CA) was applied overnight at 4 °C. After washing with PBS, secondary biotinylated antibody (Vectastain, Vector Laboratories Inc, Newark, CA) was applied for 60 min at room temperature. After additional washing, streptavidin-peroxidase complex (ExtrAvidin®-Peroxidase, Sigma-Aldrich) was applied to the specimens (incubation at room temperature, 60 min). Diaminobenzidine (DAB, Dako, Carpinteria, CA) was used as substrate for oxidation (10 min incubation, room temperature). The samples were counter-stained with Mayer’s hemalum solution to visualize cell nuclei.

All sections were imaged on a VS200 slide scanner (Olympus, Hachioji, Tokyo, Japan) using a ×20 air objective (UPLXAPO20X). The scanned sections from each animal were quantitatively analyzed using QuPath software (Bankhead et al, 2017). To quantify demyelination in spinal cord white matter sections, the pixel classifier module was used. Neurofilament H staining was analyzed with the cell detection module in the total white matter of lumbar sections and the dorsal funiculus for cervical sections.

### ELISA

Sandwich ELISA was used to measure cytokine levels in cell-free culture supernatants in MaxiSorp plates (Nunc, Roskilde, Denmark). Mouse interferon (IFN)-γ, IL-17 and IL-22 were detected using corresponding DuoSet ELISAs (R&D Systems, Minneapolis, MN), Human IFN-γ ELISA Kit (Thermo System Scientific) was used for detection of human IFN-γ, following the manufacturers’ instructions. A multi-plate reader Synergy H1 (Agilent Technology, Santa Clara, CA) was used for reading the absorbance at 450 nm and a correction at 670 nm. Samples were analyzed in duplicates. A standard curve based on known concentrations of the recombinant cytokines was used for the calculation of cytokine concentration. For all ELISA tests, the lower limit of detection was 30 pg/ml, while the upper limit of detection was 2 ng/ml.

### Reverse transcription−real-time polymerase chain reaction (RT-PCR)

Total RNA was isolated from cells using TRIzol (Thermo Fisher Scientific). Reverse transcription was performed with the use of random hexamer primers and MMLV (Moloney Murine Leukemia Virus), according to the manufacturer’s instructions (Thermo Fisher Scientific). Prepared cDNAs were amplified by using Maxima SYBR Green/ROX qPCR Master Mix (Thermo Fisher Scientific) according to the recommendations of the manufacturer in the QuantStudio 5 (Applied Biosystems, Foster City, CA). Thermocycler conditions comprised an initial step at 50 °C for 5 min, followed by a step at 95 °C for 10 min, and a subsequent two-step PCR program at 95 °C for 15 s and 60 °C for 60 s for 40 cycles. The PCR primers (Metabion, Martinsried, Germany) were as follows: *ACTB*: 5′-CAT GTA CGT TGC TAT CCA GGC-3′; 5′-CTC CTT AAT GTC ACG CAC GAT-3′; *IL1B*: 5’-ACA GAT GAA GTG CTC CTT CCA-3′; 5’-GTC GGA GAT TCG TAG CTG GAT-3’; *IL6*: 5’-AGT CCT GAT CCA GTT CCT GC-3’; 5’-CTA CAT TTG CCG AAG AGC CC-3’; *IL27*: 5’-CAA GCT GGT GTC TGG GGA TT-3’; 5’-GGA AAC ATC AGG GAG CTG CT-3’; *IL8* 5’-CAG TTT TGC CAA GGA GTG CT-3’; 5’-ACT TCT CCA CAA CCC TCT GC-3’; *IL10*: 5’-AGA CAT CAA GGC GCA TGT GA-3’; 5’-AGG CAT TCT TCA CCT GCT CC-3’; *TNF*: 5’-AGG ACC AGC TAA GAG GGA GA C-3’; 5’-CCC GGA TCA TGC TTT CAG TG-3’. PCR product accumulation was detected in real-time and for the analysis of the results QuantStudioTM Design&Analysis Software v1.5.2 (Applied Biosystems, Foster City, CA, USA) was used. Relative mRNA expression is presented as 2^−dCt^, where dCt is the difference between Ct values of a gene of interest and the endogenous control (*ACTB* - β-actin).

### Flow cytometry

The antibodies used for cell staining are listed in Table 2. The incubation for surface and intracellular staining lasted 30 or 40 min at 4 °C, respectively. Mouse Regulatory T cell Staining Kit (Thermo Fisher Scientific) was used for intracellular staining. For the detection of cytokines, cells were stimulated with eBioscience™ Cell Stimulation Cocktail (Thermo Fisher Scientific) for 4 h at 37 °C. All dilutions of antibodies were as recommended by the manufacturer and prepared in PBS supplemented with 1% BSA for surface staining, and in permeabilization buffer for intracellular staining. Cell samples were acquired on CytoFlex Flow cytometer (Beckman Coulter, Brea, CA) or BD FACSAria™ III Cell Sorter and analyzed using either CytExpert software (Beckman Coulter) or FlowJo V10.8.1 Software (BD Biosciences). Fluorescence minus one controls were used where necessary to set the gates for cell marker positivity. Results of cytofluorometry are presented as the percentage of cells within the designated cell population or the number of cells calculated according to the obtained percentage.

**Table 2:** Flow cytometry antibodies/reagents.

Target	Host	Clone	Label/dilution	Producer
Biotin	/	/	streptavidin-APC-Cy7 (1:167)	Biolegend
CD25	Rat	PC61.5	BV786 (1:40), PE (1:167), PE-Cy5.5 (1:83)	Thermo Fisher Scientific
CD3	Rat	17A2	APC-Cy7 (1:80)	Biolegend
CD4	Rat	RM4-5	eFluor 506 (1:80), BV650 (1:80)	Thermo Fisher Scientific
CD4	Rat	GK1.5	eFluor 450 (1:160), FITC (1:200), PE-Cy7 (1:40)	Thermo Fisher Scientific
CD45	Rat	30-F11	eFluor 506 (1:40), APC (1:40), PE-Cy7 (1:20)	Thermo Fisher Scientific
Fbxo24	Rabbit	Polyclonal IgG	None (1:50)	Thermo Fisher Scientific
FFAR2	Rabbit	Polyclonal	FITC (1:200)	Thermo Fisher Scientific
FoxP3	Rat	FJK-16s	PE (1:20), APC (1:20), PerCP-Cy5.5 (1:80)	Thermo Fisher Scientific
IFN-γ	Rat	XMG1.2	eFluor 450 (1:40), PerCP-Cy5.5 (1:80), APC (1:160)	Thermo Fisher Scientific
IL-10	Rat	JES5-16E3	PE (1:80), PerCP-Cy5.5 (1:80)	Thermo Fisher Scientific
IL-17	Rat	eBio17B7	FITC (1:200), PE (1:160), PerCP-Cy5.5 (1:333)	Thermo Fisher Scientific
IL-2	Rat	JES6-5H4	eFluor 450 (1:80), PE-Cy7 (1:160)	Thermo Fisher Scientific
IL-22	Rat	1H8PWSR	PE (1:160)	Thermo Fisher Scientific
IL-22	Rat	IL22JOP	APC (1:40)	Thermo Fisher Scientific
pNFκB p65	Rabbit	NFκBp65S529-H3	PE (1:20)	Thermo Fisher Scientific
pSTAT3	Mouse	LUVNKLA	PE (1:20)	Thermo Fisher Scientific
Rabbit IgG	Donkey	Polyclonal IgG	Alexa Fluor 555 (1:50)	Thermo Fisher Scientific
RORγt	Rat	AFKJS-9	PE (1:80), PerCP-eFluor 710 (1:167), APC (1:20)	Thermo Fisher Scientific
Sheep IgG	Rabbit	Polyclonal IgG	FITC (1:200)	Thermo Fisher Scientific
T-bet	Mouse	eBio4B10	eFluor 660 (1:80), PerCP-Cy5.5 (1:80)	Thermo Fisher Scientific

### FFAR2 receptor pharmacology

Sodium propionate was from Sigma-Aldrich. GLPG0974 was synthesized internally according to published methods (Pizzonero et al, 2014). All proprietary FFAR2 ligands (CAPTB, Cpd1) were synthesized internally according to published methods (Hoveyda et al, 2011; Brantis et al, 2011). Prior to use, the identity and purity of each compound were confirmed by both NMR spectroscopy and UV detection at 215 and 254 nm.

The recombinant pEFIN5-GPR43 plasmid was transfected into CHO-K1 cells for stable expression of the human FFAR2 receptor. Cells were cultured at 37 °C under 5% CO_2_ in Nutrient Ham’s F-12 medium (Thermo Fisher Scientific) supplemented with 400 µg/ml of G418, 100 U/mL penicillin, 100 µg/ml streptomycin, and 10% FBS.

The phospho-ERK1/2 (Thr202/Tyr204) was assayed using the Cisbio HTRF pERK™ kit (Cisbio, Codolet, France). CHO-*h*FFAR2 were seeded at 12500/well in Nutrient Ham’s F-12 medium containing 0.1% FBS in 384-well plates (Greiner, Kremsmünster, Austria) and kept in culture at 37 °C for 24 h. The medium was removed and cells then incubated with 2 µl of increasing concentration of test compound diluted in DMEM supplemented with 0.1% Fatty Acid Free Bovine Serum Albumin for 25 min at 37 °C. Cells were then lysed and pERK levels measured as described in the manufacturer’s protocol. The signal was read with the EnVision (Perkin Elmer, Shelton, CA).

[^35^S]GTPγS assays were performed on CHO-*h*FFAR2 cell membranes using the Scintillation Proximity Assay method. CHO-*h*FFAR2 cells in the growing phase were collected from the culture plates in Ca^2+^ and Mg^2+^-free PBS. The cells were centrifuged for 10 min at 4800 rpm at 4 °C. Pellets were then resuspended in buffer A (15 mM Tris- HCl pH 7.5; 2 mM MgCl_2_; 0.3 mM EDTA; 1 mM EGTA) and homogenized in a glass homogenizer. The crude membrane fraction was collected by two consecutive centrifugation steps at 35,000 rpm at 4 °C for 30 min, separated by a washing step in buffer A. The final pellet was resuspended in buffer B (75 mM Tris-HCl pH 7.5; 12.5 mM MgCl_2_; 0.3 mM EDTA; 1 mM EGTA; 250 mM sucrose) and flash frozen in liquid nitrogen. Protein content was determined by the BCA method (Thermo Fisher Scientific). The assay was performed in the 96-well Optiplate (Perkin Elmer) by successive addition of 60 µl of the tested compounds diluted in the assay buffer (20 mM HEPES pH 7.4, 100 mM NaCl, 10 µg/ml saponine, 30 mM MgCl_2_), 20 µl of membranes (5 µg/well) and GDP (10 µM final), pre-incubated for 15 min on ice, and then addition of 20 µl of pre-pooled [^35^S]GTPγS (0.1 nM final) and PVT-WGA beads (0.25 mg/well) (both from Perkin Elmer). The plate is then covered by a Topseal, shaken for 2 min and incubated for 2.5 h at room temperature and counted using a TopCountTM reader over a period of 30 s/well.

Human polymorphonuclear cells were isolated from fresh blood collected in heparin tubes (BD Biosciences) using the Dextran/Ficoll-Hypaque method. Briefly, blood was incubated with an equal volume of a saline dextran solution (3% Dextran T-500 in 0.9% NaCl) for 30 min at room temperature. After sedimentation, the supernatant was collected, centrifuged for 10 min at 250× *g*, and the pellet was washed in saline buffer (0.9% NaCl). Neutrophils were purified by centrifugation on Ficoll-Hypaque (Amersham Biosciences, Buckinghamshire, UK) for 40 min at 400× *g*. The remaining erythrocytes were lysed using 0.2% NaCl, then neutralized by adding 1.6% NaCl. After two washes in cold Hanks’ Balanced Salt solution (HBSS, Lonza, Walkersville, MD), neutrophils were resuspended in assay buffer and immediately used in binding or Ca^2+^ flux assays. Experiments were performed directly after cell isolation using 400,000 cells per well. Cells were loaded for 1 h with 5 µM of Fluo-4-AM with 1 mg/ml of pluronic acid (both from Thermo Fisher Scientific) in buffer (135 mM NaCl; 5 mM KCl; 1.8 mM CaCl_2_; 1 mM MgCl_2_; 10 mM HEPES; 5.6 mM Glucose; 0.1% BSA: 0.05% Gelatine; 2.5 mM probenecid). Cells were washed and incubated for 20 min at room temperature in the buffer prior to the addition of the tested antagonist and agonist compounds in a final volume of 100 µl. The fluorescent signal was directly read with an Infinate 200 pro reader (TECAN).

### RNA sequencing and analysis

Total RNA was isolated from SILP-sorted CD4^+^ T cells using Quick-RNA MicroPrep (Zymo Research, Irvine, CA) following the manufacturer’s instructions. Sample quality check, library preparation (polyA enrichment), Illumina NovaseqX Plus PE150 sequencing (≥ 6 Gb, 20 M reads), and bioinformatics analysis were performed by Novogene Co. The reads were aligned to the Ensembl mouse reference genome (GRCm39/mm39) using HISAT2 (Version 2.0.5) with the default settings. The DESeq2 program (Version 1.20.0) was used for differential expression analysis, with a minimal twofold change and a false discovery rate (FDR) of *P* < 0.05. ClusterProfiler (Version 3.8.1) software was used for GO enrichment analysis. GSEA analysis was performed by GSEA software (Version 4.3.2).

### Microbial DNA extraction and 16S rRNA sequencing

Mice were sacrificed on day 12 after immunization, and the terminal ileum was isolated by excising the distal end of the small intestine, approximately 2-3 cm long. Ileal contents were collected, and genomic DNA (gDNA) was extracted using the ZymoBIOMICS DNA Miniprep Kit (Zymo Research) following the manufacturer’s instructions. The samples were prepared and analyzed as previously described (Jevtić et al, 2025).

### Quantification of SCFA

Cecal content samples were obtained on day 12 after immunization for SCFA determination. Preparation of samples and analysis was performed according to a modified version of a published protocol (Zhang et al, 2019), as previously described (Jevtić et al, 2025).

### Statistical analysis

GraphPad Prism 9 software (GraphPad Software, San Diego, CA) was used for statistical analysis. Sample size estimations were made in accordance with our previous studies, with value for alpha set at 0.05 and power at 0.80. The calculations were performed using the Sample Size Calculator at the University of Vienna. Data were presented in histograms (mean + SD), line graphs (mean ± SEM), and box plots or violin plots (median, minimum to maximum). For data following normal distribution, a two-way analysis of variance (ANOVA) with Bonferroni’s multiple comparison test or two-tailed paired or unpaired Student’s *t* test was used, where appropriate. Mann–Whitney *U* test or Kruskal–Wallis test were used for data not following a normal distribution. *P* < 0.05 was considered statistically significant. Exact *n* values and *P* values for main figures are provided in the Appendix Table S1.

### Graphics

Figure 7 and Synopsis graphic were created with BioRender.com.

## Supplementary information

Peer Review File

Appendix

Source data Fig. 1

Source data Fig. 2

Source data Fig. 3

Source data Fig. 4

Source data Fig. 5

Source data Fig. 6

Expanded View Figures

## Data Availability

This study includes no data deposited in external repositories. The source data of this paper are collected in the following database record: biostudies:S-SCDT-10_1038-S44321-026-00517-2.
