# Supplementary material for: FFAR2 activation induces IL-22-dependent intestinal immune responses to suppress CNS autoimmunity
Source: EMBO Mol Med. 2026 Sep 3;18(10):4090–116. doi: 10.1038/s44321-026-00517-2 (PMC13658244; doi:10.1038/s44321-026-00517-2)
Supplement: Supplementary file 1 [file 44321_2026_517_MOESM1_ESM.pdf]

EMBO Molecular Medicine

FFAR2 activation induces IL-22-dependent intestinal immune responses to suppress CNS autoimmunity

Bojan Jevtić, Milica Lazarevic, Goran Stegnjaić, Suzana Stanisavljević, Ivan Pilipović, Neda Nikolovski, Zorana Milosavljević, Miljana Momcilovic, Ivan Koprivica, Natalija Jonić, Dragica Mićanović, Nataša Radulović, Maša Marković, Ksenija Mirković, Danijela Mišić, Uroš Gašić, Filip Nikolić, Nikola Veselinović, Henrike Körner, Lukas Grimm, Dimitry Lodygin, Alexander Flügel, Graeme Fraser, Tamara Saksida, Mirjana Dimitrijević, Ivana Stojanović, and Djordje Miljkovic

Corresponding author: Djordje Miljkovic (georgije_zw@yahoo.com)

Review Timeline:

Submission Date:	20th Jan 26
Editorial Decision:	23rd Feb 26
Revision Received:	17th Jul 26
Editorial Decision:	13th Aug 26
Revision Received:	19th Aug 26
Accepted:	20th Aug 26

Editor: Zeljko Durdevic

Transaction Report:

(Note: With the exception of the correction of typographical or spelling errors that could be a source of ambiguity, letters and reports are not edited. Depending on transfer agreements, referee reports obtained elsewhere may or may not be included in this compilation. Referee reports are anonymous unless the Referee chooses to sign their reports.)

23rd Feb 2026

Dear Dr. Miljkovic,

Thank you for the submission of your manuscript to EMBO Molecular Medicine. We have now received feedback from the three reviewers who agreed to evaluate your manuscript. All three referees recognize interest of the study but also raise serious concerns that should be addressed in a major revision. Focus of the revision should be on providing experimental evidence for the specificity of FFAR2 activation and subsequent IL-22 production in ameliorating EAE (referee #1), analyzing effects of Cpd1 on IL-4 expression (referee #2) and conducting a more in-depth analysis of human samples (referee #3). Considering that the revision will require extensive experimentation we think six months rather than three months would be more appropriate to provide the complete revision. If you would like to discuss further the points raised by the referees, I am available to do so via email or video. Let me know if you are interested in this option.

Further consideration of a revision that addresses reviewers' concerns in full will entail a second round of review. EMBO Molecular Medicine encourages a single round of revision only and therefore, acceptance or rejection of the manuscript will depend on the completeness of your responses included in the next, final version of the manuscript. For this reason, and to save you from any frustrations in the end, I would strongly advise against returning an incomplete revision.

We would welcome the submission of a revised version within six months for further consideration. Please let us know if you require longer to complete the revision.

I look forward to receiving your revised manuscript.

Yours sincerely,

Zeljko Durdevic

Zeljko Durdevic
Senior Editor
EMBO Molecular Medicine

*****

When preparing your revised manuscript, please refer to our guidelines: https://link.springer.com/journal/44321/submission-guidelines#cms-Revised-submissions. We perform an initial quality control of all revised manuscripts before re-review; failure to include requested items will delay the evaluation of your revision.

We require:

1) A .docx formatted version of the manuscript text (including legends for main figures, EV figures and tables). Please make sure that the changes are highlighted to be clearly visible.

2) Individual production quality figure files as .eps, .tif, .jpg (one file per figure). For guidance, download the 'Figure Guide PDF': https://media.springernature.com/original/springer-cms/rest/v1/content/27825798/data/v1.

3) A .docx formatted letter INCLUDING the reviewers' reports and your detailed point-by-point responses to their comments. As part of the EMBO Press transparent editorial process, the point-by-point response is part of the Review Process File (RPF), which will be published alongside your paper.

4) A complete author checklist, which you can download from our author guidelines. Please insert information in the checklist that is also reflected in the manuscript. The completed author checklist will also be part of the RPF.

5) Please note that all corresponding authors are required to supply an ORCID ID for their name upon submission of a revised manuscript.

6) It is mandatory to include a 'Data Availability' section after the Materials and Methods. Before submitting your revision, primary datasets produced in this study need to be deposited in an appropriate public database, and the accession numbers and database listed under 'Data Availability'. Please remember to provide a reviewer password if the datasets are not yet public.

In case you have no data that requires deposition in a public database, please state so in this section. Note that the Data Availability Section is restricted to new primary data that are part of this study.

7) For data quantification: please specify the name of the statistical test used to generate error bars and P values, the number (n) of independent experiments (specify technical or biological replicates) underlying each data point and the test used to calculate p-values in each figure legend. The figure legends should contain a basic description of n, P and the test applied. Graphs must include a description of the bars and the error bars (s.d., s.e.m.).

8) At EMBO Press we ask authors to provide source data for the main manuscript figures. You will receive a separate email with instructions for providing source data with your revised manuscript, including how to upload and organize the files.

9) Our journal encourages inclusion of *data citations in the reference list* to directly cite datasets that were re-used and obtained from public databases. Data citations in the article text are distinct from normal bibliographical citations and should directly link to the database records from which the data can be accessed. In the main text, data citations are formatted as follows: "Data ref: Smith et al, 2001" or "Data ref: NCBI Sequence Read Archive PRJNA342805, 2017". In the Reference list, data citations must be labeled with "[DATASET]". A data reference must provide the database name, accession number/identifiers and a resolvable link to the landing page from which the data can be accessed at the end of the reference.

10) We replaced Supplementary Information with Expanded View (EV) Figures and Tables that are collapsible/expandable online. A maximum of 5 EV Figures can be typeset. EV Figures should be cited as 'Figure EV1, Figure EV2" etc... in the text and their respective legends should be included in the main text after the legends of regular figures.

- For the figures that you do NOT wish to display as Expanded View figures, they should be bundled together with their legends in a single PDF file called *Appendix*, which should start with a short Table of Content. Appendix figures should be referred to in the main text as: "Appendix Figure S1, Appendix Figure S2" etc.

- Additional Tables/Datasets should be labeled and referred to as Table EV1, Dataset EV1, etc. Legends have to be provided in a separate tab in case of .xls files. Alternatively, the legend can be supplied as a separate text file (README) and zipped together with the Table/Dataset file.

11) The paper explained: EMBO Molecular Medicine articles are accompanied by a summary of the articles to emphasize the major findings in the paper and their medical implications for the non-specialist reader. Please provide a draft summary of your article highlighting

- the medical issue you are addressing,

- the results obtained and

- their clinical impact.

This may be edited to ensure that readers understand the significance and context of the research. Please refer to any of our published articles for an example.

12) Author contributions: You will be asked to provide CRediT (Contributor Role Taxonomy) terms in the submission system. These replace a narrative author contribution section in the manuscript.

13) A Conflict of Interest statement should be provided in the main text.

14) Every published paper includes a 'Synopsis' to further enhance discoverability. Synopses are displayed on the journal webpage and are freely accessible to all readers. They include a short stand first (maximum of 300 characters, including space) as well as 2-5 one-sentences bullet points that summarizes the paper. Please write the bullet points to summarize the key NEW findings. They should be designed to be complementary to the abstract - i.e. not repeat the same text. We encourage inclusion of key acronyms and quantitative information (maximum of 30 words / bullet point). Please use the passive voice. Please attach these in a separate file or send them by email, we will incorporate them accordingly.

Please also suggest a striking image or visual abstract to illustrate your article as a PNG file 550 px wide x 300-600 px high.

15) Include a Reagents and Tools Table as part of the Methods section, which can be downloaded from our author guidelines.

EMBO Molecular Medicine has a "scooping protection" policy, whereby similar findings that are published by others during review or revision are not a criterion for rejection. Should you decide to submit a revised version, I do ask that you get in touch after three months if you have not completed it, to update us on the status.

***** Reviewer's comments *****

Referee #1 (Comments on Novelty/Model System for Author):

The study relies heavily on pharmacological tools (Cpd1 and anti-IL-22 antibodies) without sufficient controls to rule out off-target effects.

Referee #1 (Remarks for Author):

Jevtić et al. investigate the potential of Cpd1, a FFAR2 agonist, to suppress autoimmune inflammation in the EAE model via an interleukin-22 (IL-22)-dependent mechanism. The authors report that Cpd1 treatment ameliorates EAE disease severity and is associated with the stimulation of regulatory T cells (Tregs) and Group 3 Innate Lymphoid Cells (ILC3s) within the intestine. Furthermore, they utilize anti-IL-22 neutralizing antibodies to suggest that the protective effect of Cpd1 is dependent on IL-22 signaling.

While the topic of targeting FFAR2 to modulate neuroinflammation is timely and potentially interesting, the current dataset is premature and does not fully support the central conclusions. Specifically, the study relies heavily on pharmacological tools (Cpd1 and anti-IL-22 antibodies) without sufficient controls to rule out off-target effects. Consequently, the claim that the observed amelioration of EAE is driven strictly by specific FFAR2 activation and subsequent IL-22 production remains unproven. Additionally, the manuscript lacks a detailed characterization of the immunological landscape and neuropathology required to substantiate the mechanism of action.

Main concerns:

1. Almost all small molecule agonists, including Cpd1, have off-target effects that can confound interpretation. The authors have not provided convincing evidence that Cpd1 ameliorates EAE severity specifically through FFAR2 activation.
2. The conclusion that the mechanism is IL-22-dependent rests on the use of anti-IL-22 antibodies. Antibody neutralization experiments can be prone to non-specific binding or incomplete neutralization, and the authors have not provided evidence that this antibody selectively and exclusively blocks IL-22 in this model.
3. The authors do not perform detailed analyses to characterize the immune response in the peripheral immune system and the CNS of EAE mice.
4. The authors do not perform detailed analyses to determine tissue damage in the CNS of EAE mice, such as demyelination, oligodendrocyte death, and axon degeneration.

Referee #2 (Remarks for Author):

The revision opinion

This research demonstrates that the FFAR2-specific agonist Cpd1, administered orally, effectively ameliorates central nervous system autoimmune symptoms during the effector phase of the EAE model. Its mechanism of action relies on IL-22-mediated signaling, Specifically, it stimulates the activation of intestinal Tregs and ILC3s, inhibits pathways associated with the proliferation and activation of intestinal CD4+ T cells, and reduces immune cell infiltration and inflammatory responses in the central nervous system. Furthermore, it inhibits inflammatory cytokine secretion in PBMCs from human MS patients, suggesting that FFAR2 agonists hold promise as potential therapeutic agents for autoimmune diseases such as multiple sclerosis. However, the article still has some issues that need to be addressed to improve its quality：

1． In the study, RNA sequencing results revealed that Cpd1 upregulates IL-4 expression in CD4+ T cells within the small intestinal lamina propria while simultaneously suppressing proinflammatory signaling pathways such as IFN-γ. As a core cytokine of the Th2 immune response, IL-4 directly inhibits the differentiation of naive CD4+ T cells into Th1/Th17 cells. The authors should supplement this information.

2． To investigate whether Cpd1 can affect human cells, peripheral blood mononuclear cells (PBMCs) were obtained from MS patients and healthy controls. The cells were activated using ConA. Why not activate the TCR using specific antigens?

Referee #3 (Comments on Novelty/Model System for Author):

The story is interesting and the quality of the mouse study is sufficiently high. However, for the paper to reach the higher level (suitable to EMOB MM), the part of human immunology should be strengthened. At this stage, they should show more data to make a good translational immunology paper.

Referee #3 (Remarks for Author):

Short chain fatty acid (SCFA) has immunoregulatory potentials and reduced intake of SCFA may have roles in human health and diseases. Cpd1 is a small molecule ligand which may mimic the effects of SCFA as it binds to free fatty acid receptor 2 (FFAR2), receptor for SCFA. The authors have examined the effects of Cpd1 in mouse EAE model and human MS, to clarify the immunomodulatory effects of Cpd1. The significant advance made was that they have revealed the role of IL-22 producing innate lymphoid cells type 3 (ILC3) and Treg cells in the effects of Cpd1. The therapeutic effects of Cpd1 is significant in the mouse EAE model, and this effect was abrogated by injection of anti-IL-22 antibody. Another important finding was that the main target of Cpd1are located within the gut and the therapeutic effects in EAE could be explained by Cpd1 effects within gut. Although only small intestine derived cells CD4+ T cells were analyzed, the mouse study is well designed and their message appears to be based on good experimental results. In contrast, analysis of human samples is not satisfactory. They showed simply that Cpd1 would suppress the Con A induced activation of human peripheral blood T cells, which is not supportive for their discussion on the role of ILC3 and Treg in Cpd1 effects.

Comments
1. Is Cpd1 not absorbed from the gut? If PK/PD data of Cpd1 is available, the authors may refer to it.
2. Cpd1 appears to modulate gut microbiota in mouse. Is there any reasonable consistency compared to their study in rats?
3. Study for the in vitro effects of Cpd1 on human T cells should be further sophisticated, if Cpd1 is not simply a immunosuppressive drug.

All three referees recognize interest of the study but also raise serious concerns that should be addressed in a major revision. Focus of the revision should be on providing experimental evidence for the specificity of FFAR2 activation and subsequent IL-22 production in ameliorating EAE (referee #1), analyzing effects of Cpd1 on IL-4 expression (referee #2) and conducting a more in-depth analysis of human samples (referee #3).

We have addressed all the comments raised by the referees and paid particular attention to the issues emphasized by the Editor. Specifically, novel data showing specificity of FFAR2 activation by Cpd1, data on the effect of Cpd1 on IL-4 expression in our experimental system, as well as deeper *in vitro* analysis of the effect of Cpd1 on human PBMC are incorporated in the revised version. Detailed point-by-point response to the reviewer's comments follows.

Referee #1 (Comments on Novelty/Model System for Author):

The study relies heavily on pharmacological tools (Cpd1 and anti-IL-22 antibodies) without sufficient controls to rule out off-target effects.

Referee #1 (Remarks for Author):

Jevtić et al. investigate the potential of Cpd1, a FFAR2 agonist, to suppress autoimmune inflammation in the EAE model via an interleukin-22 (IL-22)-dependent mechanism. The authors report that Cpd1 treatment ameliorates EAE disease severity and is associated with the stimulation of regulatory T cells (Tregs) and Group 3 Innate Lymphoid Cells (ILC3s) within the intestine. Furthermore, they utilize anti-IL-22 neutralizing antibodies to suggest that the protective effect of Cpd1 is dependent on IL-22 signaling.
While the topic of targeting FFAR2 to modulate neuroinflammation is timely and potentially interesting, the current dataset is premature and does not fully support the central conclusions. Specifically, the study relies heavily on pharmacological tools (Cpd1 and anti-IL-22 antibodies) without sufficient controls to rule out off-target effects. Consequently, the claim that the observed amelioration of EAE is driven strictly by specific FFAR2 activation and subsequent IL-22 production remains unproven. Additionally, the manuscript lacks a detailed characterization of the immunological landscape and neuropathology required to substantiate the mechanism of action.
Main concerns:
1. Almost all small molecule agonists, including Cpd1, have off-target effects that can confound interpretation. The authors have not provided convincing evidence that Cpd1 ameliorates EAE severity specifically through FFAR2 activation.

The receptor pharmacology of Cpd1 is presented in the revised manuscript (Fig S5 and corresponding text: Page 22, line 9). Cpd1 has the same maximal activity (%E_{max}) with 100-1000 fold superior potency to propionate in GTPγS and ERK signaling assays performed on CHO cells expressing the human FFAR2 receptor; CAPTB and GLPG0974 were also tested to demonstrate the absence of receptor activation for these FFAR2 reference antagonists in these assays. Receptor selectivity is demonstrated using assays conducted on human polymorphonuclear cells collected from healthy donors; the Ca^{2+} flux response to Cpd1 is completely blocked by the selective antagonists CAPTB and GLPG0974, respectively. Thus, we are confident that Cpd1 acts through FFAR2 in our study.

2. The conclusion that the mechanism is IL-22-dependent rests on the use of anti-IL-22 antibodies. Antibody neutralization experiments can be prone to non-specific binding or incomplete neutralization, and the authors have not provided evidence that this antibody selectively and exclusively blocks IL-22 in this model.

To respond to the reviewer's comment regarding the specificity of the anti-IL-22 neutralizing antibody (clone IL22JOP), we agree that selective neutralization is essential for interpreting pathway-dependent effects. Importantly, as we detail below, both established literature and

data already contained within our manuscript (including newly generated data) demonstrate unequivocally that IL22JOP selectively and exclusively blocks IL-22 in our experimental setting. Together, these sources provide a level of validation sufficient to avoid further ad-hoc experiments that would only replicate what is already firmly documented.

i. Validation and specificity of the anti-IL-22 antibody (IL22JOP)

The neutralizing anti-IL-22 antibody used in our study (rat monoclonal clone IL22JOP, Thermo Fisher Scientific) has been extensively validated in the literature as a selective IL-22–blocking antibody. As documented in the manufacturer's datasheet and multiple peer-reviewed articles (see below), IL22JOP reliably inhibits IL-22 bioactivity in both mouse and human systems. These data include functional neutralization assays demonstrating that IL22JOP blocks IL-22–dependent IL-10 induction (ND50 10–40 ng/mL). This antibody has been used repeatedly and successfully in published studies to define IL-22 function in immune and epithelial systems. Collectively, these independent validations establish the antibody's specificity and neutralizing activity. The following is the list of papers in which IL22JOP was demonstrated to neutralize IL-22.

Yeaman et al., Mechanisms of NDV-3 vaccine efficacy in MRSA skin versus invasive infection. Proc Natl Acad Sci U S A. 2014 Dec 23;111(51):E5555-63. doi: 10.1073/pnas.1415610111.

Peng et al., Interleukin-22 promotes T helper 1 (Th1)/Th17 immunity in chlamydial lung infection. Mol Med. 2014 Mar 20;20(1):109-19. doi: 10.2119/molmed.2013.00115.

Pinto et al., Joint production of IL-22 participates in the initial phase of antigen-induced arthritis through IL-1β production. Arthritis Res Ther. 2015 Sep 2;17(1):235. doi: 10.1186/s13075-015-0759-2.

Busbee et al., Indole-3-carbinol prevents colitis and associated microbial dysbiosis in an IL-22-dependent manner. JCI Insight. 2020 Jan 16;5(1):e127551. doi: 10.1172/jci.insight.127551.

Wu et al., Interleukin-1β secretion induced by mucosa-associated gut commensal bacteria promotes intestinal barrier repair. Gut Microbes. 2022 Jan-Dec;14(1):2014772. doi: 10.1080/19490976.2021.2014772.

Holloman et al., Aryl Hydrocarbon Receptor Activation Ameliorates Acute Respiratory Distress Syndrome through Regulation of Th17 and Th22 Cells in the Lungs. mBio. 2023 Apr 25;14(2):e0313722. doi: 10.1128/mbio.03137-22.

ii. Use of appropriate controls: isotype control adequately excludes non-specific effects

In accordance with accepted immunological practice, we included a matched rat IgG2a isotype control in all experiments involving IL22JOP. This control is the correct method to exclude non-specific Fc-mediated interactions, injection-related effects, or antibody-associated immune activation. Importantly, the isotype control did not affect any Cpd1-mediated phenotypes, whereas IL22JOP selectively reversed the effects attributed to IL-22. Thus, the specificity of the observed effects is supported by internal experimental controls, in line with standard practices for antibody-neutralization studies.

iii. "Irrelevant binding antibody" as negative control is not appropriate or necessary

Irrelevant antibody that binds an unrelated antigen but does not interfere with the IL-22 pathway might not be considered methodologically appropriate, as binding antibodies inherently introduce unpredictable biological effects by engaging their respective antigens and potentially triggering receptor signaling, crosslinking, or activation pathways unrelated to IL-22. This would confound interpretation and reduce—not increase—specificity of the experimental design.

In contrast, the isotype control used in our study is intentionally non-binding, thereby representing the correct and widely accepted control to rule out non-specific effects. Thus, the additional control suggested by the reviewer would not strengthen the conclusions and is not used in standard antibody-neutralization studies.

iv. Functional alignment with IL-22 biology supports pathway specificity (Fig 6)

Importantly, the observed effects after IL-22 neutralization closely match established IL-22 biology. In co-culture, IL22JOP selectively reversed the suppression of IL-17 (but not IFN-γ), and *in vivo*, anti-IL-22 completely abrogated the therapeutic benefit of Cpd1, whereas the isotype had no effect. This selective reversal is entirely consistent with the mechanistic role of IL-22 and cannot be explained by non-specific antibody activity. These observations constitute

explicit, internal demonstration of IL-22-exclusive neutralization, something that no external experiment could improve upon.
v. **To further support validity of IL22JOP as the neutralizing antibody, levels of IL-22 were measured in supernatants of cell cultures of SILP cells exposed to the antibody. As the result IL-22 levels in the cell cultures supernatants were annulled in the presence of IL22JOP. These data are incorporated in the revised version of the manuscript as Fig 6H.**

Together, the use of a literature-validated neutralizing antibody, the inclusion of a proper isotype control, and the IL-22–consistent functional outcomes robustly support our conclusion that IL-22 is a key mediator of Cpd1 activity. For these reasons, we respectfully maintain that IL22JOP's specificity is both scientifically established and experimentally demonstrated within our dataset and the conclusions drawn in our manuscript remain fully supported. We hope this detailed explanation satisfactorily addresses the reviewer's concern and confirms the appropriateness of our experimental design.

3. The authors do not perform detailed analyses to characterize the immune response in the peripheral immune system and the CNS of EAE mice.

Following the reviewer's comment additional data obtained with SILP cells (Fig 2 M-R) and lymph node cells (Fig S3). As for immune cells within the CNS, our aim was to explore if Cpd1 restricts inflammation within the CNS, and we think that we have provided sufficient evidence to show that Cpd1 is indeed efficient in limiting infiltration into the CNS. Also, effects of Cpd1 treatment on $CD4^+$ T cells as the major pathogenic drivers of EAE are presented in the manuscript. Thus we think that additional analysis of immune cells within the CNS will not add to the value of conclusions of the study. We have to emphasize that due to the profound limiting effect of Cpd1 on immune cell numbers within the CNS, relatively high number of mice is required to obtain samples that can be efficiently analysed by flow cytometry. This raises ethical issues about necessity of using additional mice for this purpose. Alternatively, we have performed additional analyses to determine tissue damage in the CNS of EAE mice as suggested by the reviewer (see the next point).

4. The authors do not perform detailed analyses to determine tissue damage in the CNS of EAE mice, such as demyelination, oligodendrocyte death, and axon degeneration.

Luxol fast blue staining of spinal cord sections for demyelination, as well as SMI32 staining for axonal damage were performed. Data are presented in Fig S2 of the revised manuscript and commented accordingly in the revised text (Page 16, line 1).

Referee #2 (Remarks for Author):

The revision opinion
This research demonstrates that the FFAR2-specific agonist Cpd1, administered orally, effectively ameliorates central nervous system autoimmune symptoms during the effector phase of the EAE model. Its mechanism of action relies on IL-22-mediated signaling, Specifically, it stimulates the activation of intestinal Tregs and ILC3s, inhibits pathways associated with the proliferation and activation of intestinal CD4+ T cells, and reduces immune cell infiltration and inflammatory responses in the central nervous system. Furthermore, it inhibits inflammatory cytokine secretion in PBMCs from human MS patients, suggesting that FFAR2 agonists hold promise as potential therapeutic agents for autoimmune diseases such as multiple sclerosis. However, the article still has some issues that need to be addressed to improve its quality :

1. In the study, RNA sequencing results revealed that Cpd1 upregulates IL-4 expression in CD4+ T cells within the small intestinal lamina propria while simultaneously suppressing proinflammatory signaling pathways such as IFN-γ. As a core cytokine of the Th2 immune response, IL-4 directly inhibits the differentiation of naive CD4+ T cells into Th1/Th17 cells. The authors should supplement this information.

Following the reviewer's suggestion we have analysed the effects of Cpd1 on purified CD4+ T cells. Cpd1 did not upregulate release of IL-4 from CD4+ T cells (Fig 4H). Also, neutralizing anti-IL-4 antibody was applied to co-cultivation of lamina propria cells and MOG_{35-55}-stimulated lymph node cells, yet without effect on IFN-gamma or IL-17 levels (Fig 6 D, E). These data suggest that IL-4 is not a mediator of Cpd1 effects in our system and are discussed in the revised version of the manuscript (Discussion, Page 38, line 23).

2. To investigate whether Cpd1 can affect human cells, peripheral blood mononuclear cells (PBMCs) were obtained from MS patients and healthy controls. The cells were activated using ConA. Why not activate the TCR using specific antigens?

PBMC obtained from healthy donors were challenged with PPD. Cpd1 efficiently reduced IFN-gamma release from PPD-stimulated human PBMC (Fig 5E).

Referee #3 (Comments on Novelty/Model System for Author):

The story is interesting and the quality of the mouse study is sufficiently high. However, for the paper to reach the higher level (suitable to EMOB MM), the part of human immunology should be strengthened. At this stage, they should show more data to make a good translational immunology paper.

Referee #3 (Remarks for Author):

Short chain fatty acid (SCFA) has immunoregulatory potentials and reduced intake of SCFA may have roles in human health and diseases. Cpd1 is a small molecule ligand which may mimic the effects of SCFA as it binds to free fatty acid receptor 2 (FFAR2), receptor for SCFA. The authors have examined the effects of Cpd1 in mouse EAE model and human MS, to clarify the immunomodulatory effects of Cpd1. The significant advance made was that they have revealed the role of IL-22 producing innate lymphoid cells type 3 (ILC3) and Treg cells in the effects of Cpd1. The therapeutic effects of Cpd1 is significant in the mouse EAE model, and this effect was abrogated by injection of anti-IL-22 antibody. Another important finding was that the main target of Cpd1are located within the gut and the therapeutic effects in EAE could be explained by Cpd1 effects within gut. Although only small intestine derived cells CD4+ T cells were analyzed, the mouse study is well designed and their message appears to be based on good experimental results. In contrast, analysis of human samples is not satisfactory. They showed simply that Cpd1 would suppress the Con A induced activation of human peripheral blood T cells, which is not supportive for their discussion on the role of ILC3 and Treg in Cpd1 effects.

Comments
1. Is Cpd1 not absorbed from the gut? If PK/PD data of Cpd1 is available, the authors may refer to it.

Cpd1 is orally bioavailable. For example, the pharmacokinetic curve following oral dosing to non-fasted, female mice is presented below.

Figure for reviewers removed

Our composite dataset indicates that the gut is the primary target organ consistent with the findings presented in the manuscript that SILP ILC3 and Treg, as well as the microbiota, are altered in response to Cpd1 treatment. The oral bioavailability of Cpd1 leaves open the possibility that its gut pharmacology could be triggered by the presence of the compound in the lumen and/or circulating in the body.

2. Cpd1 appears to modulate gut microbiota in mouse. Is there any reasonable consistency compared to their study in rats?

Cpd1 has effects on microbiota in both mice and rats, however different bacterial strains were affected across species. We have compared data in mice from this study with data on rats from our previous study and we have provided additional comment on the comparison between species in the revised manuscript (Discussion, Page 40, line 7).

3. Study for the in vitro effects of Cpd1 on human T cells should be further sophisticated, if Cpd1 is not simply a immunosuppressive drug.

Effects of Cpd1 on human PBMC *in vitro* were further explored. PBMC were challenged with a specific antigen (PPD) in the absence or presence of Cpd1. As the results Cpd1 downregulated IFN-gamma release from PBMC in a dose-dependent fashion (Fig 5E). Further, RT-PCR was performed for multiple cytokines and it was shown that Cpd1 did not affect expression of IL-1beta, IL-6, TNF, IL-8, and IL-27, while it increased expression of IL-10 (Fig 5 F-K). Thus, with these additional data we can be confident that the effect of Cpd1 on human PBMC is not immunosuppressive, but rather immunomodulatory. The novel data are commented in the revised version of the manuscript (Page 32, line 23).

13th Aug 2026

Dear Dr. Miljkovic,

Thank you for the submission of your manuscript to EMBO Molecular Medicine. We have now received feedback from the two reviewers who agreed to evaluate your manuscript. As you will see from their reports pasted below both referees support publication of your manuscript. Therefore, I am pleased to inform you that we will be able to accept your manuscript pending the following final amendments:

1) Authors: Email address for natasa.radulovic@ibiss.bg.ac.rs no longer valid, please update.
2) Figures:
- Remove all figures from the manuscript file and move their legends to the end of the file.
- Please rename Figures S1 - S7 to Figures EV1 - EV7 and update their callout in the main manuscript text. EV figure legends should be placed in the manuscript file after main figure legends under the heading "Expanded View Figure Legends".
3) Source Data: Please upload source data for the main figures as one zipped file per figure. Fig 4I/J/K are mislabeled, please correct.
4) Title: Please consider revising the title to better emphasize the main findings, e.g. "FFAR2 activation induces IL-22-dependent intestinal immune responses to suppress CNS autoimmunity"
5) Abstract: I have gone through your text and revised it (see below). Please review it for accuracy and amend as you see fit:

Short-chain fatty acids (SCFAs) protect against central nervous system (CNS) autoimmunity, but the receptor-mediated mechanisms underlying these effects remain poorly understood, limiting their therapeutic exploitation. Here, we show that selective oral activation of free fatty acid receptor 2 (FFAR2) with the synthetic agonist Cpd1 effectively ameliorates established experimental autoimmune encephalomyelitis (EAE), demonstrating therapeutic efficacy after the onset of the autoimmune response. Cpd1 treatment promoted regulatory T cells and type 3 innate lymphoid cells in the intestine while reducing inflammatory infiltration in the CNS, indicating that modulation of intestinal immunity is sufficient to restrain neuroinflammation. Transcriptomic analysis of intestinal $CD4^+$ T cells revealed broad suppression of pathways controlling T-cell activation and proliferation. Mechanistically, the therapeutic effects of Cpd1 required interleukin-22 (IL-22), as antibody-mediated IL-22 neutralization abolished disease protection, identifying IL-22 as a key downstream effector of FFAR2 signaling. Together, these findings establish selective FFAR2 agonism as a tractable strategy to therapeutically modulate the gut-CNS immune axis and identify FFAR2 as a promising target for the treatment of multiple sclerosis and other autoimmune diseases.

6) In the main manuscript file, please do the following:
- Please address all comments suggested by our data editors listed below:
o Figure legends:
1. Please note that the legends for figure 5 are not provided in a sequential manner (legend for figure 5D is provided before legend of figure 5C). This needs to be rectified.
2. Please note that the exact p values are not provided in the legends of figures 1B-G,I-P; 2D-G,I-L,N,P; 4B,C,E,F,J; 5B,E,I; 6B,C,G,H,J.
- Remove "data not shown" (p. 38).
- Please rename "Background" to "Introduction".
- Please update the order and the headings of the manuscript sections to: Abstract / The Paper Explained / Introduction / Results / Discussion / Methods / Data Availability / Acknowledgements / Disclosure and Competing Interests Statement / References / Figure Legends / Tables / Expanded View Figure Legends
- Rename Suppl Tables 1 and 2 to Table 1 and Table 2.
- In Methods, provide the antibody dilutions that were used for each antibody.
- In Methods, provide the statement that informed consent was obtained from all human subjects and that in addition to the WMA Declaration of Helsinki the experiments also conformed to the principles set out in the Department of Health and Human Services Belmont Report.
- Indicate in legends exact n and exact p values, not a range, along with the statistical test used. To keep the figures "clear" some authors found providing an Appendix table Sx with all exact p-values preferable. You are welcome to do this if you want to.
- Remove BioRender reference from figure legends and add to Methods in a dedicated section using this format:
Graphics:
(some of the... OR Figure #... OR synopsis) Graphics were created with BioRender.com.
- Rename "Competing interest" to "Disclosure Statement & Competing Interests". We updated our journal's competing interests policy in January 2022 and request authors to consider both actual and perceived competing interests. Please review the policy https://www.embopress.org/competing-interests and update your competing interests if necessary.
- Author contributions: Please remove it from the manuscript and specify author contributions in our submission system. CRediT has replaced the traditional author contributions section because it offers a systematic machine-readable author contributions format that allows for more effective research assessment. You are encouraged to use the free text boxes beneath each contributing author's name to add specific details on the author's contribution. More information is available in our guide to authors:

https://www.embopress.org/page/journal/17574684/authorguide#authorshipguidelines
- Correct the reference citation in the text and reference list. In the text a reference should be cited by author and year of publication. Include a space between a word and the opening parenthesis of the reference that follows. In the reference list, citations should be listed in alphabetical order. Where there are more than 10 authors on a paper, 10 will be listed, followed by "et al.". Also, please remove DOIs. Please check "Author Guidelines" for more information.
https://link.springer.com/journal/44321/submission-guidelines#cms-Reference-guidelines
7) Funding: Merge it with "Acknowledgments" and ensure that all funders are listed in our submission system.
8) The Paper Explained: Please provide "The Paper Explained" and add it to the main manuscript text. Please check any of recently published EMBO Mol Med research articles for reference.
9) Synopsis: Every published paper now includes a 'Synopsis' to further enhance discoverability. Synopses are displayed on the journal webpage and are freely accessible to all readers. They include separate synopsis image and synopsis text.
- Synopsis image: Please provide a visual abstract as a high-resolution .jpeg file exactly 550 px-wide x 300 pixels high to illustrate your article.
- Synopsis text: Please provide a short standfirst (maximum of 300 characters, including space) as well as 2-5 one sentence bullet points that summarize the paper as a .doc file. Please write the bullet points to summarize the key NEW findings. They should be designed to be complementary to the abstract - i.e. not repeat the same text. We encourage inclusion of key acronyms and quantitative information (maximum of 30 words / bullet point). Please use the passive voice.
- Please check your synopsis text and image before submission with your revised manuscript. Please be aware that in the proof stage minor corrections only are allowed (e.g., typos).
10) As part of the EMBO Publications transparent editorial process (see our Editorial at http://embomolmed.embopress.org/content/2/9/329), EMBO Molecular Medicine will publish online a Review Process File (RPF) to accompany accepted manuscripts. This file will be published in conjunction with your paper and will include the anonymous referee reports, your point-by-point response and all pertinent correspondence relating to the manuscript. Let us know if you want to remove or not any figures from it prior to publication. Please note that the Authors checklist will be published at the end of the RPF.
11) Please provide a point-by-point letter INCLUDING my comments as well as the reviewer's reports and your detailed responses (as Word file).

I look forward to reading a new revised version of your manuscript as soon as possible.

Yours sincerely,

Zeljko Durdevic

Zeljko Durdevic
Senior Editor
EMBO Molecular Medicine

*** Instructions to submit your revised manuscript ***

*** PLEASE NOTE *** As part of the EMBO Publications transparent editorial process initiative (see our Editorial at https://www.embopress.org/doi/pdf/10.1002/emmm.201000094), EMBO Molecular Medicine will publish online a Review Process File to accompany accepted manuscripts.

In the event of acceptance, this file will be published in conjunction with your paper and will include the anonymous referee reports, your point-by-point response and all pertinent correspondence relating to the manuscript. If you do NOT want this file to be published, please inform the editorial office at contact@embomolmed.org.

*****

When preparing your revised manuscript, please refer to our guidelines: https://link.springer.com/journal/44321/submission-guidelines#cms-Revised-submissions. We perform an initial quality control of all revised manuscripts before re-review; failure to include requested items will delay the evaluation of your revision.

We require:

1) A .docx formatted version of the manuscript text (including legends for main figures, EV figures and tables). Please make sure that the changes are highlighted to be clearly visible.

2) Individual production quality figure files as .eps, .tif, .jpg (one file per figure). For guidance, download the 'Figure Guide PDF': https://media.springernature.com/original/springer-cms/rest/v1/content/27825798/data/v1.

3) A .docx formatted letter INCLUDING the reviewers' reports and your detailed point-by-point responses to their comments. As part of the EMBO Press transparent editorial process, the point-by-point response is part of the Review Process File (RPF), which will be published alongside your paper.

4) A complete author checklist, which you can download from our author guidelines. Please insert information in the checklist that is also reflected in the manuscript. The completed author checklist will also be part of the RPF.

5) Please note that all corresponding authors are required to supply an ORCID ID for their name upon submission of a revised manuscript.

6) It is mandatory to include a 'Data Availability' section after the Materials and Methods. Before submitting your revision, primary datasets produced in this study need to be deposited in an appropriate public database, and the accession numbers and database listed under 'Data Availability'. Please remember to provide a reviewer password if the datasets are not yet public.

In case you have no data that requires deposition in a public database, please state so in this section. Note that the Data Availability Section is restricted to new primary data that are part of this study.

7) For data quantification: please specify the name of the statistical test used to generate error bars and P values, the number (n) of independent experiments (specify technical or biological replicates) underlying each data point and the test used to calculate p-values in each figure legend. The figure legends should contain a basic description of n, P and the test applied. Graphs must include a description of the bars and the error bars (s.d., s.e.m.).

8) At EMBO Press we ask authors to provide source data for the main manuscript figures. You will receive a separate email with instructions for providing source data with your revised manuscript, including how to upload and organize the files.

9) Our journal encourages inclusion of *data citations in the reference list* to directly cite datasets that were re-used and obtained from public databases. Data citations in the article text are distinct from normal bibliographical citations and should directly link to the database records from which the data can be accessed. In the main text, data citations are formatted as follows: "Data ref: Smith et al, 2001" or "Data ref: NCBI Sequence Read Archive PRJNA342805, 2017". In the Reference list, data citations must be labeled with "[DATASET]". A data reference must provide the database name, accession number/identifiers and a resolvable link to the landing page from which the data can be accessed at the end of the reference.

10) We replaced Supplementary Information with Expanded View (EV) Figures and Tables that are collapsible/expandable online. A maximum of 5 EV Figures can be typeset. EV Figures should be cited as 'Figure EV1, Figure EV2" etc... in the text and their respective legends should be included in the main text after the legends of regular figures.

- For the figures that you do NOT wish to display as Expanded View figures, they should be bundled together with their legends in a single PDF file called *Appendix*, which should start with a short Table of Content. Appendix figures should be referred to in the main text as: "Appendix Figure S1, Appendix Figure S2" etc.

- Additional Tables/Datasets should be labeled and referred to as Table EV1, Dataset EV1, etc. Legends have to be provided in a separate tab in case of .xls files. Alternatively, the legend can be supplied as a separate text file (README) and zipped together with the Table/Dataset file.

11) The paper explained: EMBO Molecular Medicine articles are accompanied by a summary of the articles to emphasize the major findings in the paper and their medical implications for the non-specialist reader. Please provide a draft summary of your article highlighting

- the medical issue you are addressing,

- the results obtained and

- their clinical impact.

This may be edited to ensure that readers understand the significance and context of the research. Please refer to any of our

published articles for an example.

12) Author contributions: You will be asked to provide CRediT (Contributor Role Taxonomy) terms in the submission system. These replace a narrative author contribution section in the manuscript.

13) A Conflict of Interest statement should be provided in the main text.

14) Every published paper includes a 'Synopsis' to further enhance discoverability. Synopses are displayed on the journal webpage and are freely accessible to all readers. They include a short stand first (maximum of 300 characters, including space) as well as 2-5 one-sentences bullet points that summarizes the paper. Please write the bullet points to summarize the key NEW findings. They should be designed to be complementary to the abstract - i.e. not repeat the same text. We encourage inclusion of key acronyms and quantitative information (maximum of 30 words / bullet point). Please use the passive voice. Please attach these in a separate file or send them by email, we will incorporate them accordingly.

Please also suggest a striking image or visual abstract to illustrate your article as a PNG file 550 px wide x 300 px high.

15) Include a Reagents and Tools Table as part of the Methods section, which can be downloaded from our author guidelines.

EMBO Molecular Medicine has a "scooping protection" policy, whereby similar findings that are published by others during review or revision are not a criterion for rejection. Should you decide to submit a revised version, I do ask that you get in touch after three months if you have not completed it, to update us on the status.

*Additional important information regarding Figures

Each figure should be given in a separate file and should have the following resolution:
Graphs 800-1,200 DPI
Photos 400-800 DPI
Colour (only CMYK) 300-400 DPI"

Figures are not edited by the production team. All lettering should be the same size and style; figure panels should be indicated by capital letters (A, B, C etc). Gridlines are not allowed except for log plots. Figures should be numbered in the order of their appearance in the text with Arabic numerals. Each Figure must have a separate legend and a caption is needed for each panel.

*Additional important information regarding figures and illustrations can be found at
https://media.springernature.com/original/springer-cms/rest/v1/content/27825798/data/v1

***** Reviewer's comments *****

Referee #1 (Remarks for Author):

No further comments.

Referee #2 (Comments on Novelty/Model System for Author):

The mechanism by which FFAR2 agonists alleviate neuroinflammation via intestinal IL-22 is innovative; the authors have provided a comprehensive response to the questions raised.

The authors addressed the remaining editorial issues.

20th Aug 2026

Dear Dr. Miljkovic,

We are pleased to inform you that your manuscript is accepted for publication and is now being sent to our publisher to be included in the next available issue of EMBO Molecular Medicine.

Your manuscript will be processed for publication by EMBO Press. It will be copy edited and you will receive page proofs prior to publication. Please note that you will be contacted by Springer Nature Author Services to complete licensing and payment information.

You may qualify for financial assistance for your publication charges - either via a Springer Nature fully open access agreement or an EMBO initiative. Check your eligibility: https://link.springer.com/journal/44321/how-to-publish-with-us

Should you be planning a Press Release on your article, please notify the production team via this form in order to coordinate publication and release dates: https://forms.cloud.microsoft/e/eH4A8JVP6g

If you have any questions, please do not hesitate to contact the Editorial Office. Thank you for your contribution to EMBO Molecular Medicine.

Yours sincerely,
Zeljko Durdevic

Zeljko Durdevic
Senior Editor
EMBO Molecular Medicine

>>> Please note that it is EMBO Molecular Medicine policy for the transcript of the editorial process (containing referee reports and your response letter) to be published as an online supplement to each paper. If you do NOT want this, you will need to inform the Editorial Office via email immediately. More information is available here: https://link.springer.com/partners/embo-press/editorial-policies#Peer%20review

EMBO Press Author Checklist

Corresponding Author Name: Djordje Miljkovic	
Journal Submitted to: EMBO Molecular Medicine	
Manuscript Number: EMM-2026-23351	

USEFUL LINKS FOR COMPLETING THIS FORM
The EMBO Journal - Author Guidelines
EMBO Reports - Author Guidelines
Molecular Systems Biology - Author Guidelines
EMBO Molecular Medicine - Author Guidelines

Reporting Checklist for Life Science Articles (updated January
This checklist is adapted from Materials Design Analysis Reporting (MDAR) Checklist for Authors. MDAR establishes a minimum set of requirements in transparent reporting in the life sciences (see Statement of Task: 10.31222/osf.io/9sm4x). Please follow the journal's guidelines in preparing your
Please note that a copy of this checklist will be published alongside your article.

Abridged guidelines for figures
1. Data
The data shown in figures should satisfy the following conditions:
- the data were obtained and processed according to the field's best practice and are presented to reflect the results of the experiments in an accurate and unbiased manner.
- ideally, figure panels should include only measurements that are directly comparable to each other and obtained with the same assay.
- plots include clearly labeled error bars for independent experiments and sample sizes. Unless justified, error bars should not be shown for technical
- if n<5, the individual data points from each experiment should be plotted. Any statistical test employed should be justified.
- Source Data should be included to report the data underlying figures according to the guidelines set out in the authorship guidelines on Data

2. Captions
Each figure caption should contain the following information, for each panel where they are relevant:
- a specification of the experimental system investigated (eg cell line, species name).
- the assay(s) and method(s) used to carry out the reported observations and measurements.
- an explicit mention of the biological and chemical entity(ies) that are being measured.
- an explicit mention of the biological and chemical entity(ies) that are altered/varied/perturbed in a controlled manner.
- the exact sample size (n) for each experimental group/condition, given as a number, not a range;
- a description of the sample collection allowing the reader to understand whether the samples represent technical or biological replicates (including how many animals, litters, cultures, etc.).
- a statement of how many times the experiment shown was independently replicated in the laboratory.
- definitions of statistical methods and measures:
  - common tests, such as t-test (please specify whether paired vs. unpaired), simple χ2 tests, Wilcoxon and Mann-Whitney tests, can be unambiguously identified by name only, but more complex techniques should be described in the methods section;
  - are tests one-sided or two-sided?
  - are there adjustments for multiple comparisons?
  - exact statistical test results, e.g., P values = x but not P values < x;
  - definition of 'center values' as median or average;
  - definition of error bars as s.d. or s.e.m.

Please complete ALL of the questions below.
Select "Not Applicable" only when the requested information is not relevant for your study.

Materials

Newly Created Materials	Information included in the manuscript?	In which section is the information available? (Reagents and Tools Table, Materials and Methods, Figures, Data Availability Section)
New materials and reagents need to be available; do any restrictions apply?	Yes	Material and Methods, EAE in mice, lines 5-7

Antibodies	Information included in the manuscript?	In which section is the information available? (Reagents and Tools Table, Materials and Methods, Figures, Data Availability Section)
For **antibodies** provide the following information:<br>- Commercial antibodies: RRID (if possible) or supplier name, catalogue number and or/clone number<br>- Non-commercial: RRID or citation	Yes	Material and Methods, Suppl. Table 2, Structured methods

DNA and RNA sequences	Information included in the manuscript?	In which section is the information available? (Reagents and Tools Table, Materials and Methods, Figures, Data Availability Section)
**Short novel DNA or RNA including primers, probes**: provide the sequences.	Yes	Material and Methods, Reverse Transcription−Real-Time Polymerase Chain Reaction (RT-PCR), Structured methods

Cell materials	Information included in the manuscript?	In which section is the information available? (Reagents and Tools Table, Materials and Methods, Figures, Data Availability Section)
**Cell lines:** Provide species information, strain. Provide accession number in repository **OR** supplier name, catalog number, clone number, and/**OR** RRID.	Yes	Material and Methods, FFAR2 receptor pharmacology, Structured methods
**Primary cultures:** Provide species, strain, sex of origin, genetic modification status.	Yes	Material and Methods, Cell isolation and culture
Report if the cell lines were recently **authenticated** (e.g., by STR profiling) and tested for mycoplasma contamination.	Not Applicable	NA

Experimental animals	Information included in the manuscript?	In which section is the information available? (Reagents and Tools Table, Materials and Methods, Figures, Data Availability Section)
**Laboratory animals or Model organisms:** Provide species, strain, sex, age, genetic modification status. Provide accession number in repository **OR** supplier name, catalog number, clone number, **OR** RRID.	Yes	Material and Methods, Structured methods
**Animal observed in or captured from the field:** Provide species, sex, and age where possible.	Not Applicable	NA
Please detail **housing and husbandry conditions**.	Yes	Material and Methods, Experimental animals

Plants and microbes	Information included in the manuscript?	In which section is the information available? (Reagents and Tools Table, Materials and Methods, Figures, Data Availability Section)
**Plants:** provide species and strain, ecotype and cultivar where relevant, unique accession number if available, and source (including location for collected wild specimens).	Not Applicable	NA
**Microbes:** provide species and strain, unique accession number if available, and source.	Not Applicable	NA

Human research participants	Information included in the manuscript?	In which section is the information available? (Reagents and Tools Table, Materials and Methods, Figures, Data Availability Section)
If collected and within the bounds of privacy constraints report on age, sex and gender or ethnicity for all study participants.	Yes	Material and Methods, Human samples

Core facilities	Information included in the manuscript?	In which section is the information available? (Reagents and Tools Table, Materials and Methods, Figures, Data Availability Section)
If your work benefited from core facilities, was their service mentioned in the acknowledgments section?	Not Applicable	NA

Design

Study protocol	Information included in the manuscript?	In which section is the information available? (Reagents and Tools Table, Materials and Methods, Figures, Data Availability Section)
If study protocol has been **pre-registered, provide DOI in the manuscript**. For clinical trials, provide the trial registration number **OR** cite DOI.	Not Applicable	NA
Report the **clinical trial registration number** (at ClinicalTrials.gov or equivalent), where applicable.	Not Applicable	NA

Laboratory protocol	Information included in the manuscript?	In which section is the information available? (Reagents and Tools Table, Materials and Methods, Figures, Data Availability Section)
Provide DOI OR other citation details if **external detailed step-by-step protocols** are available.	Not Applicable	NA

Experimental study design and statistics	Information included in the manuscript?	In which section is the information available? (Reagents and Tools Table, Materials and Methods, Figures, Data Availability Section)
Include a statement about **sample size** estimate even if no statistical methods were used.	Yes	Material and Methods, Statistical analysis
Were any steps taken to minimize the effects of subjective bias when allocating animals/samples to treatment (e.g. **randomization procedure**)? If yes, have they been described?	Yes	Material and Methods, Experimental animals
Include a statement about **blinding** even if no blinding was done.	Yes	Material and Methods, Experimental animals
Describe **inclusion/exclusion criteria** if samples or animals were excluded from the analysis. Were the criteria pre-established?<br><br>If sample or data points were omitted from analysis, report if this was due to attrition or intentional exclusion and provide justification.	Not Applicable	NA
For every figure, are **statistical tests** justified as appropriate? Do the data meet the assumptions of the tests (e.g., normal distribution)? Describe any methods used to assess it. Is there an estimate of variation within each group of data? Is the variance similar between the groups that are being statistically compared?	Yes	Figure captions.

Sample definition and in-laboratory replication	Information included in the manuscript?	In which section is the information available? (Reagents and Tools Table, Materials and Methods, Figures, Data Availability Section)
In the figure legends: state number of times the experiment was **replicated** in laboratory.	Yes	Figure legends.
In the figure legends: define whether data describe **technical or biological replicates**.	Yes	Figure legends.

Ethics

Ethics	Information included in the manuscript?	In which section is the information available? (Reagents and Tools Table, Materials and Methods, Figures, Data Availability Section)
Studies involving **human participants**: State details of **authority granting ethics approval** (IRB or equivalent committee(s), provide reference number for approval.	Not Applicable	NA
Studies involving **human participants**: Include a statement confirming that **informed consent** was obtained from all subjects and that the experiments conformed to the principles set out in the WMA Declaration of Helsinki and the Department of Health and Human Services Belmont Report.	Yes	Material and Methods, Human samples
Studies involving **human participants:** For publication of **patient photos**, include a statement confirming that consent to publish was obtained.	Not Applicable	NA
Studies involving experimental **animals**: State details of **authority granting ethics approva**l (IRB or equivalent committee(s), provide reference number for approval. Include a statement of compliance with ethical regulations.	Yes	Material and Methods, Experimental animals
Studies involving **specimen and field samples:** State if relevant **permits** obtained, provide details of authority approving study; if none were required, explain why.	Not Applicable	NA

Dual Use Research of Concern (DURC)	Information included in the manuscript?	In which section is the information available? (Reagents and Tools Table, Materials and Methods, Figures, Data Availability Section)
Could your study fall under dual use research restrictions? Please check biosecurity documents and list of **select agents and toxins** (CDC): https://www.selectagents.gov/sat/list.htm	Not Applicable	
If you used a select agent, is the security level of the lab appropriate and reported in the manuscript?	Not Applicable	
If a study is subject to dual use research of concern regulations, is the name of the **authority granting approval and reference number** for the regulatory approval provided in the manuscript?	Not Applicable	

Reporting

The MDAR framework recommends adoption of discipline-specific guidelines, established and endorsed through community initiatives. Journals have their own policy about requiring specific guidelines and recommendations to complement MDAR.

Adherence to community standards	Information included in the manuscript?	In which section is the information available? (Reagents and Tools Table, Materials and Methods, Figures, Data Availability Section)
State if relevant guidelines or checklists (e.g., **ICMJE, MIBBI, ARRIVE, PRISMA**) have been followed or provided.	Not Applicable	
For **tumor marker prognostic studies,** we recommend that you follow the **REMARK** reporting guidelines (see link list at top right). See author guidelines, under 'Reporting Guidelines'. Please confirm you have followed these guidelines.	Not Applicable	
For **phase II and III randomized controlled trials**, please refer to the **CONSORT** flow diagram (see link list at top right) and submit the CONSORT checklist (see link list at top right) with your submission. See author guidelines, under 'Reporting Guidelines'. Please confirm you have submitted this list.	Not Applicable	

Data Availability

Data availability	Information included in the manuscript?	In which section is the information available? (Reagents and Tools Table, Materials and Methods, Figures, Data Availability Section)
Have **primary datasets** been deposited according to the journal's guidelines (see 'Data Deposition' section) and the respective accession numbers provided in the Data Availability Section?	Yes	Source data
Were **human clinical and genomic datasets** deposited in a public access-controlled repository in accordance to ethical obligations to the patients and to the applicable consent agreement?	Not Applicable	
Are **computational models** that are central and integral to a study available without restrictions in a machine-readable form? Were the relevant accession numbers or links provided?	Not Applicable	
If publicly available data were reused, provide the respective **data citations in the reference list**.	Yes	Reference list
