# Supplementary material for: FFAR2 activation induces IL-22-dependent intestinal immune responses to suppress CNS autoimmunity
Source: EMBO Mol Med. 2026 Sep 3;18(10):4090–116. doi: 10.1038/s44321-026-00517-2 (PMC13658244; doi:10.1038/s44321-026-00517-2)
Supplement: Supplementary file 2 [file 44321_2026_517_MOESM2_ESM.pdf]

Appendix Table S1

Information on the statistical test applied, sample size and exact p value used in analyses in the main figures.

Table of contents: data for Figure 1, Figure 2, Figure 3, Figure 4, Figure 5, Figure 6

**Figure 1**	Groups	Statistical test	Sample size (n)	Exact *p* value
B	EAE vs. EAE+Cpd1	ANOVA (two-way) with Bonferroni	n = 11 per group	< 0.0001
C	EAE vs. EAE+Cpd1	two-tailed Student's t test	n = 10 per group	0.0102
D	EAE vs. EAE+Cpd1	two-tailed Student's t test	n = 10 per group	0.0102
E	EAE vs. EAE+Cpd1	two-tailed Student's t test	n = 11 per group	< 0.0001
F	EAE vs. EAE+Cpd1	two-tailed Student's t test	n = 11 per group	0.0001
G	EAE vs. EAE+Cpd1	Mann-Whitney U test	n = 11 per group	0.0317
I	EAE vs. EAE+Cpd1	Mann-Whitney U test	n = 12 per group	0.0004
J	EAE vs. EAE+Cpd1	two-tailed Student's t test	n1 = 12, n2 = 5	0.0068
K	EAE vs. EAE+Cpd1	Mann-Whitney U test	n = 6 per group	0.0043
L	EAE vs. EAE+Cpd1	Mann-Whitney U test	n = 6 per group	0.0152
M	EAE vs. EAE+Cpd1	Mann-Whitney U test	n = 6 per group	0.013
N	EAE vs. EAE+Cpd1	Mann-Whitney U test	n = 6 per group	0.0022
O	EAE vs. EAE+Cpd1	two-tailed Student's t test	n = 6 per group	0.0076
P	EAE vs. EAE+Cpd1	Mann-Whitney U test	n = 6 per group	0.0152
**Figure 2**	Groups	Statistical test	Sample size (n)	Exact *p* value
B	EAE vs. EAE+Cpd1	two-tailed Student's t test	n = 5 per group	0.1869
C	EAE vs. EAE+Cpd1	two-tailed Student's t test	n = 5 per group	0.3785
D	EAE vs. EAE+Cpd1	two-tailed Student's t test	n = 5 per group	0.014
E	EAE vs. EAE+Cpd1	two-tailed Student's t test	n = 5 per group	0.0386
F	EAE vs. EAE+Cpd1	two-tailed Student's t test	n = 5 per group	0.0472
G	EAE vs. EAE+Cpd1	two-tailed Student's t test	n = 5 per group	0.0022
H	EAE vs. EAE+Cpd1	two-tailed Student's t test	n = 5 per group	0.3752
I	EAE vs. EAE+Cpd1	two-tailed Student's t test	n = 5 per group	0.0087
J	EAE vs. EAE+Cpd1	two-tailed Student's t test	n = 5 per group	0.0138
K	EAE vs. EAE+Cpd1	two-tailed Student's t test	n = 5 per group	0.0133
L	EAE vs. EAE+Cpd1	two-tailed Student's t test	n = 5 per group	0.007
M	EAE vs. EAE+Cpd1	two-tailed Student's t test	n = 5 per group	0.9708
N	EAE vs. EAE+Cpd1	two-tailed Student's t test	n = 5 per group	0.0008
O	EAE vs. EAE+Cpd1	two-tailed Student's t test	n = 5 per group	0.6027
P	EAE vs. EAE+Cpd1	two-tailed Student's t test	n = 5 per group	0.0086
Q	EAE vs. EAE+Cpd1	two-tailed Student's t test	n = 5 per group	0.5855
R	EAE vs. EAE+Cpd1	two-tailed Student's t test	n = 7 per group	0.054

**Figure 3**	Groups	Statistical test	Sample size (n)	Exact *p* value	Gene
D	EAE vs. EAE+Cpd1	one-tailed Student's t test	n = 4 per group	0.0086	Il4
	EAE vs. EAE+Cpd1	one-tailed Student's t test	n = 4 per group	0.0400	Foxp3
	EAE vs. EAE+Cpd1	one-tailed Student's t test	n = 4 per group	0.0088	Il27ra
	EAE vs. EAE+Cpd1	one-tailed Student's t test	n = 4 per group	0.0989	Il23a
	EAE vs. EAE+Cpd1	one-tailed Student's t test	n = 4 per group	0.1136	Notch1
	EAE vs. EAE+Cpd1	one-tailed Student's t test	n = 4 per group	0.0864	Zbtb7b
	EAE vs. EAE+Cpd1	one-tailed Student's t test	n = 4 per group	0.1126	Slamf6
	EAE vs. EAE+Cpd1	one-tailed Student's t test	n = 4 per group	0.1800	Rorc
	EAE vs. EAE+Cpd1	one-tailed Student's t test	n = 4 per group	0.1887	Traf3ip2
	EAE vs. EAE+Cpd1	one-tailed Student's t test	n = 4 per group	0.2154	Nlrp10
	EAE vs. EAE+Cpd1	one-tailed Student's t test	n = 4 per group	0.2392	Loxl3
	EAE vs. EAE+Cpd1	one-tailed Student's t test	n = 4 per group	0.4906	Prkcg
	EAE vs. EAE+Cpd1	one-tailed Student's t test	n = 4 per group	0.2767	Card9
	EAE vs. EAE+Cpd1	one-tailed Student's t test	n = 4 per group	0.3468	Ly9
	EAE vs. EAE+Cpd1	one-tailed Student's t test	n = 4 per group	0.3422	Clec4n
	EAE vs. EAE+Cpd1	one-tailed Student's t test	n = 4 per group	0.3487	Tyk2
	EAE vs. EAE+Cpd1	one-tailed Student's t test	n = 4 per group	0.3624	Il2
	EAE vs. EAE+Cpd1	one-tailed Student's t test	n = 4 per group	0.3627	Tnfsf18
	EAE vs. EAE+Cpd1	one-tailed Student's t test	n = 4 per group	0.3948	Il6ra
	EAE vs. EAE+Cpd1	one-tailed Student's t test	n = 4 per group	0.4061	Batf
	EAE vs. EAE+Cpd1	one-tailed Student's t test	n = 4 per group	0.3464	Il17ra
	EAE vs. EAE+Cpd1	one-tailed Student's t test	n = 4 per group	0.3932	Tgfb1
	EAE vs. EAE+Cpd1	one-tailed Student's t test	n = 4 per group	0.4266	Nfkbid
	EAE vs. EAE+Cpd1	one-tailed Student's t test	n = 4 per group	0.4068	Malt1
	EAE vs. EAE+Cpd1	one-tailed Student's t test	n = 4 per group	0.4447	Nlrp3
	EAE vs. EAE+Cpd1	one-tailed Student's t test	n = 4 per group	0.4718	Il6
	EAE vs. EAE+Cpd1	one-tailed Student's t test	n = 4 per group	0.4883	Otud5
	EAE vs. EAE+Cpd1	one-tailed Student's t test	n = 4 per group	0.4641	Entpd7
	EAE vs. EAE+Cpd1	one-tailed Student's t test	n = 4 per group	0.4911	Mir326
	EAE vs. EAE+Cpd1	one-tailed Student's t test	n = 4 per group	0.3180	Phb2
	EAE vs. EAE+Cpd1	one-tailed Student's t test	n = 4 per group	0.4652	Stat3
	EAE vs. EAE+Cpd1	one-tailed Student's t test	n = 4 per group	0.4688	Rc3h2
	EAE vs. EAE+Cpd1	one-tailed Student's t test	n = 4 per group	0.4715	Smad7
	EAE vs. EAE+Cpd1	one-tailed Student's t test	n = 4 per group	0.4611	Irf4
	EAE vs. EAE+Cpd1	one-tailed Student's t test	n = 4 per group	0.4280	Jak2
E	EAE vs. EAE+Cpd1	one-tailed Student's t test	n = 4 per group	0.1851	Txk
	EAE vs. EAE+Cpd1	one-tailed Student's t test	n = 4 per group	0.2319	Parp14
	EAE vs. EAE+Cpd1	one-tailed Student's t test	n = 4 per group	0.2956	Arg1
	EAE vs. EAE+Cpd1	one-tailed Student's t test	n = 4 per group	0.4286	Stat1
	EAE vs. EAE+Cpd1	one-tailed Student's t test	n = 4 per group	0.4372	Nlrc5
	EAE vs. EAE+Cpd1	one-tailed Student's t test	n = 4 per group	0.4632	Parp9
	EAE vs. EAE+Cpd1	one-tailed Student's t test	n = 4 per group	0.4802	Med1
	EAE vs. EAE+Cpd1	one-tailed Student's t test	n = 4 per group	0.4894	Ifngr1
	EAE vs. EAE+Cpd1	one-tailed Student's t test	n = 4 per group	0.4503	Nmi
	EAE vs. EAE+Cpd1	one-tailed Student's t test	n = 4 per group	0.4280	Jak2
	EAE vs. EAE+Cpd1	one-tailed Student's t test	n = 4 per group	0.4632	Otop1

	EAE vs. EAE+Cpd1	one-tailed Student's t test	n = 4 per group	0.4380	Ptpn2
	EAE vs. EAE+Cpd1	one-tailed Student's t test	n = 4 per group	0.4332	Socs1
	EAE vs. EAE+Cpd1	one-tailed Student's t test	n = 4 per group	0.3423	Trp53
	EAE vs. EAE+Cpd1	one-tailed Student's t test	n = 4 per group	0.2027	Igtp
	EAE vs. EAE+Cpd1	one-tailed Student's t test	n = 4 per group	0.2345	Ifng
	EAE vs. EAE+Cpd1	one-tailed Student's t test	n = 4 per group	0.1307	Dnaja3
	EAE vs. EAE+Cpd1	one-tailed Student's t test	n = 4 per group	0.1094	Cdc37
	EAE vs. EAE+Cpd1	one-tailed Student's t test	n = 4 per group	0.1442	Hpx
	EAE vs. EAE+Cpd1	one-tailed Student's t test	n = 4 per group	0.0073	Irgm1
	EAE vs. EAE+Cpd1	one-tailed Student's t test	n = 4 per group	0.0134	Irf1

**Figure 4**	Groups	Statistical test	Sample size (n)	Exact *p* value
B	without Cpd1 vs. with Cpd1	paired two-tailed Student's t test	n = 6 per group	0.0453
C	without Cpd1 vs. with Cpd1	paired two-tailed Student's t test	n = 6 per group	0.0123
E	without Cpd1 vs. with Cpd1	paired two-tailed Student's t test	n = 3 per group	0.0429
F	without Cpd1 vs. with Cpd1	paired two-tailed Student's t test	n = 3 per group	0.0424
G	without Cpd1 vs. with Cpd1	paired two-tailed Student's t test	n = 4 per group	0.4623
H	without Cpd1 vs. with Cpd1	paired two-tailed Student's t test	n = 4 per group	0.1268
J	without Cpd1 vs. with Cpd1	paired two-tailed Student's t test	n = 4 per group	0.0134
K	without Cpd1 vs. with Cpd1	paired two-tailed Student's t test	n = 4 per group	0.0813

**Figure 5**	Groups	Statistical test	Sample size (n)	Exact *p* value
B	without Cpd1 vs. with Cpd1	paired two-tailed Student's t test	n = 5 per group	0.0014
C	Control, RRMS, SPMS and PPMS	Kruskal-Wallis test	Control n =45<br>RRMS n = 36<br>SPMS n = 20<br>PPMS n = 43	0.3436
D	without Cpd1 vs. with Cpd1	paired two-tailed Student's t test	n = 6 per group	0.0052
E	Cpd1 concentration (0 µM, 25 µM, 50 µM and 100 µM)	ANOVA (one-way) with Dunnett's	n = 5 per group<br>(0 vs. 25 µM)<br>(0 vs. 50 µM)<br>(0 v.s 100 µM)	0.0129<br>0.0004<br>0.0001
F	without Cpd1 vs. with Cpd1	paired two-tailed Student's t test	n = 6 per group	0.1832

G	without Cpd1 vs. with Cpd1	paired two-tailed Student's t test	n = 5 per group	0.1469
H	without Cpd1 vs. with Cpd1	paired two-tailed Student's t test	n = 5 per group	0.6518
I	without Cpd1 vs. with Cpd1	paired two-tailed Student's t test	n = 5 per group	0.0379
J	without Cpd1 vs. with Cpd1	paired two-tailed Student's t test	n = 5 per group	0.4498
K	without Cpd1 vs. with Cpd1	paired two-tailed Student's t test	n = 5 per group	0.1404
Figure 6	Groups	Statistical test	Sample size (n)	Exact *p* value
B	without Cpd1 vs. with Cpd1	paired one-tailed Student's t test	n = 6 per group	0.0295
C	without Cpd1 vs. with Cpd1	paired one-tailed Student's t test	n = 6 per group	0.0061
D	ISO vs. aIL-4	paired one-tailed Student's t test	n = 4 per group	0.0502
E	ISO vs. aIL-4	paired one-tailed Student's t test	n = 4 per group	0.4950
F	ISO vs. aIL-22	paired one-tailed Student's t test	n = 4 per group	0.1158
G	ISO vs. aIL-22	paired one-tailed Student's t test	n = 4 per group	0.0423
H	ISO vs. aIL-22	paired one-tailed Student's t test	n = 4 per group	0.0028
J	EAE, EAE+Cpd1+ISO and EAE+Cpd1 +aIL-22	ANOVA (two-way) with Bonferroni	n = 10 per group	< 0.0001
