# Supplementary material for: FFAR2 activation induces IL-22-dependent intestinal immune responses to suppress CNS autoimmunity
Source: EMBO Mol Med. 2026 Sep 3;18(10):4090–116. doi: 10.1038/s44321-026-00517-2 (PMC13658244; doi:10.1038/s44321-026-00517-2)
Supplement: Supplementary file 3 [file 44321_2026_517_MOESM9_ESM.pdf]

Expanded View Figures

Figure EV1. Effect of Cpd1 on EAE.

EAE mice were treated orally with 20 mg/kg body weight Cpd1 (EAE+Cpd1, $n = 7$) or vehicle (EAE, $n = 7$) (days 9–22) (**A**). The severity of EAE was assessed by daily clinical scores up to day 40 (**B**), day of onset (**C**), duration of disease (**D**), cumulative clinical score (**E**), mean clinical score (**F**), and maximal clinical score (**G**). EAE mice were treated orally with 10 mg/kg body weight Cpd1 (EAE+Cpd1, $n = 8$) or vehicle (EAE, $n = 8$) (days 9 to 15) (**H**). The severity of EAE was assessed by daily clinical scores up to day 40 (**I**), day of onset (**J**), duration of disease (**K**), cumulative clinical score (**L**), mean clinical score (**M**), and maximal clinical score (**N**). EAE was induced in Dark Agouti rats by immunization with spinal cord homogenate, and rats were treated orally with 15 mg/kg body weight Cpd1 (EAE+Cpd1, $n = 6$) or vehicle (EAE, $n = 6$) (days 7–15) (**O**). The severity of EAE was assessed by daily clinical scores up to day 40 (**P**), day of onset (**Q**), duration of disease (**R**), cumulative clinical score (**S**), mean clinical score (**T**), and maximal clinical score (**U**). The data presented are biological replicates from one of two independent experiments. Data are presented as mean ± SEM (**B**, **I**, **P**), or mean ± SD (**C**–**G**, **J**–**N**, **Q**–**U**), and analyzed by two-way ANOVA with Bonferroni's multiple comparison test (**B**, **I**, **P**), or Student's *t* test (**C**–**G**, **L**, **M**, **S**, **T**), or Mann–Whitney test (**J**, **K**, **N**, **Q**, **R**, **U**). $^{*}P < 0.05$, $^{****}P < 0.0001$, ns, not significant.

 © The Author(s)

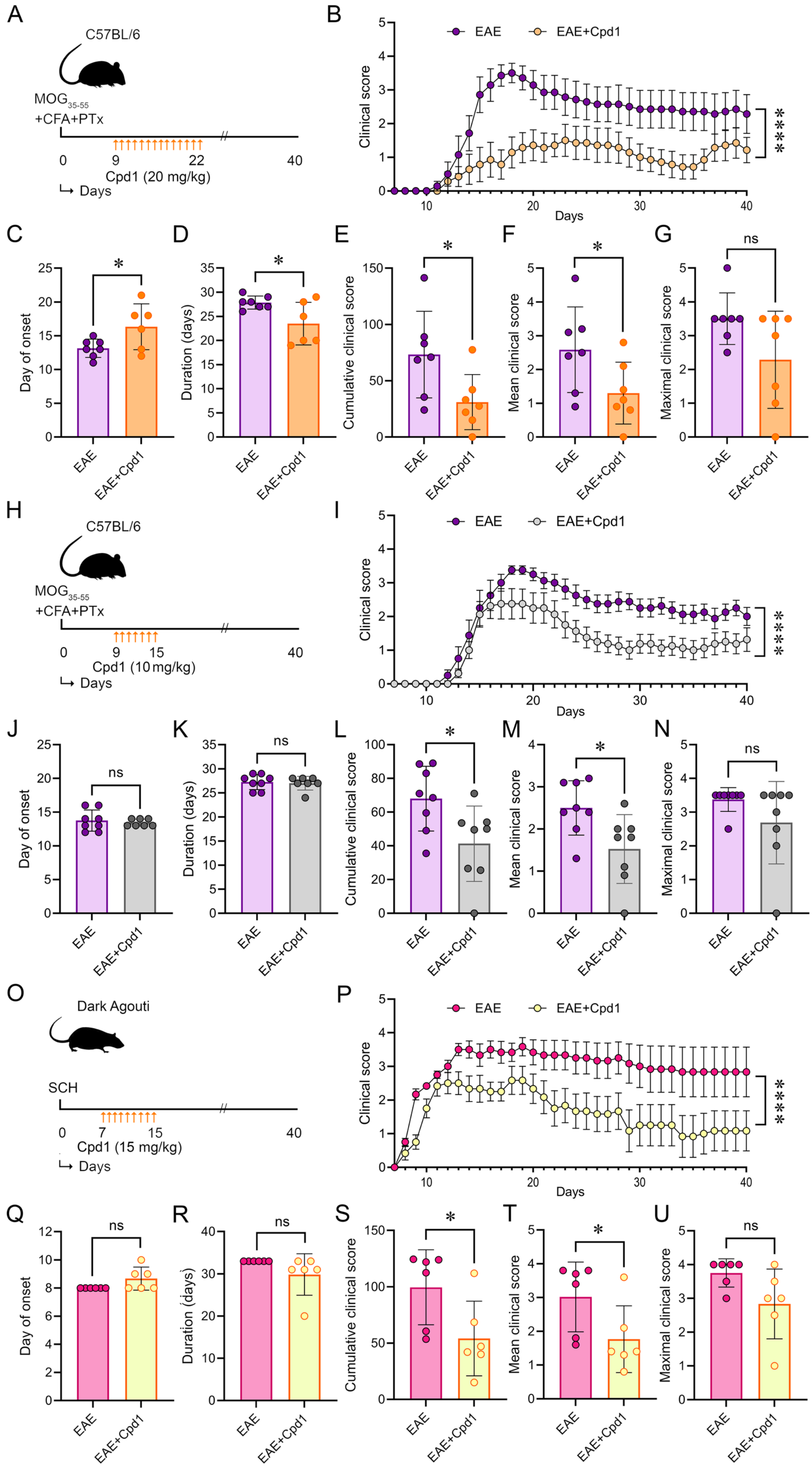
A
C57BL/6
MOG35-55
+CFA+PTx
0 9 22 40
Cpd1 (20 mg/kg)
Days
B
EAE
EAE+Cpd1
Clinical score
Days
****
C
Day of onset
D
Duration (days)
E
Cumulative clinical score
F
Mean clinical score
G
Maximal clinical score
ns
EAE
EAE+Cpd1
H
C57BL/6
MOG35-55
+CFA+PTx
0 9 15 40
Cpd1 (10 mg/kg)
Days
I
EAE
EAE+Cpd1
Clinical score
Days
****
J
K
L
M
N
O
Dark Agouti
SCH
0 7 15 40
Cpd1 (15 mg/kg)
Days
P
EAE
EAE+Cpd1
Clinical score
Days
****
Q
R
S
T
U

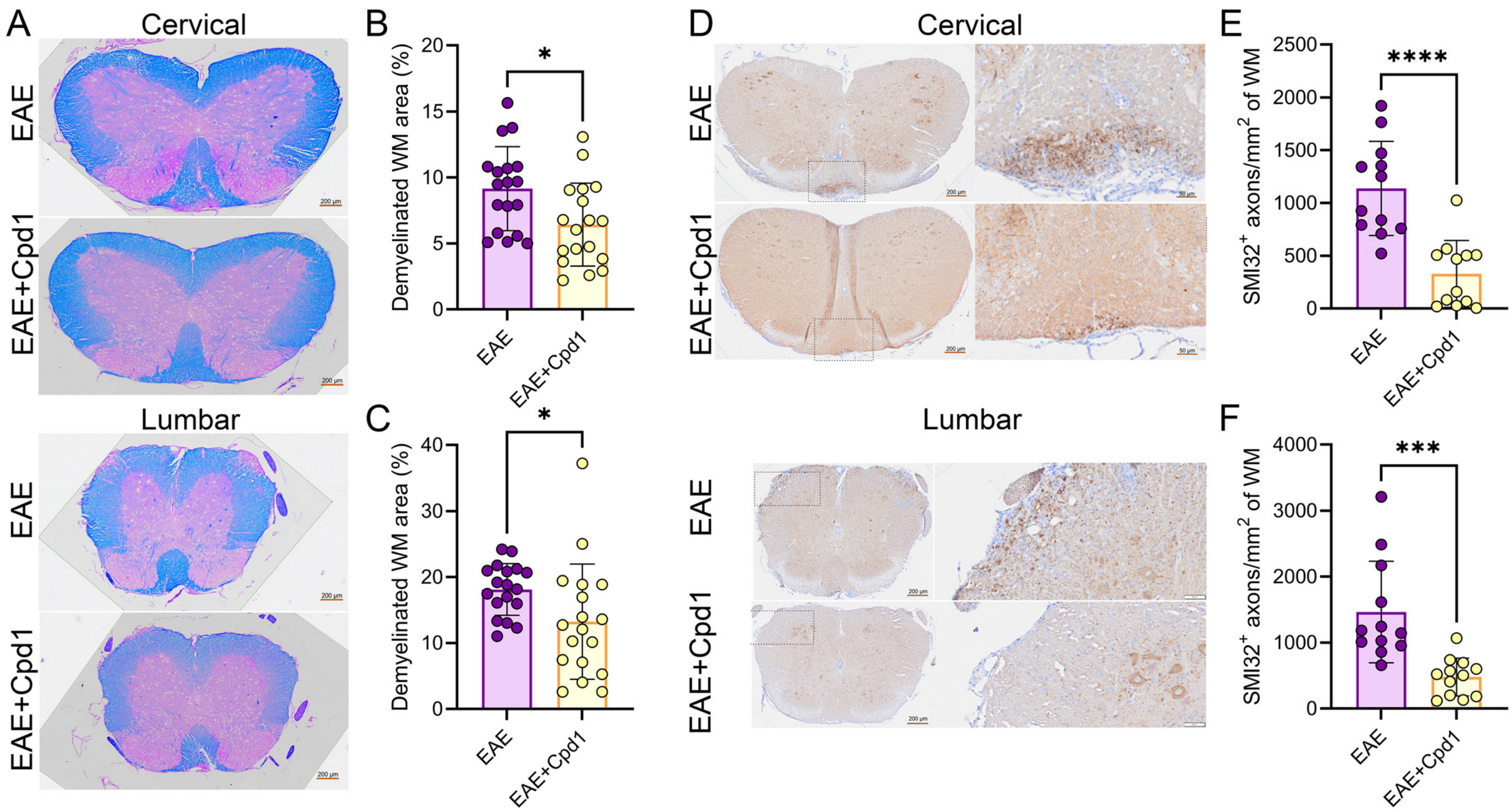

Figure EV2. Effect of Cpd1 on demyelination and axonal damage in EAE.

EAE mice were treated orally with 20 mg/kg body weight Cpd1 (EAE+Cpd1, $n = 11$) or vehicle (EAE, $n = 11$) (days 9–15). Mice were sacrificed on day 17. LFB representative images of spinal cord sections are presented (**A**). Quantification of LFB in cervical (**B**) and lumbar spinal cord sections (**C**) (3 mice, 6 sections/mouse, $n = 18$). SMI32 immunostaining representative images of spinal cord sections are presented (**D**). Quantification of SMI32 immunostaining in cervical (**E**) and lumbar spinal cord sections (**F**) (3 mice, 4 sections/mouse, $n = 12$). The data presented are biological replicates from one experiment. Data are presented as mean ± SD and analyzed by Student's *t* test. *$P < 0.05$, ***$P < 0.001$, ****$P < 0.0001$.

 © The Author(s)

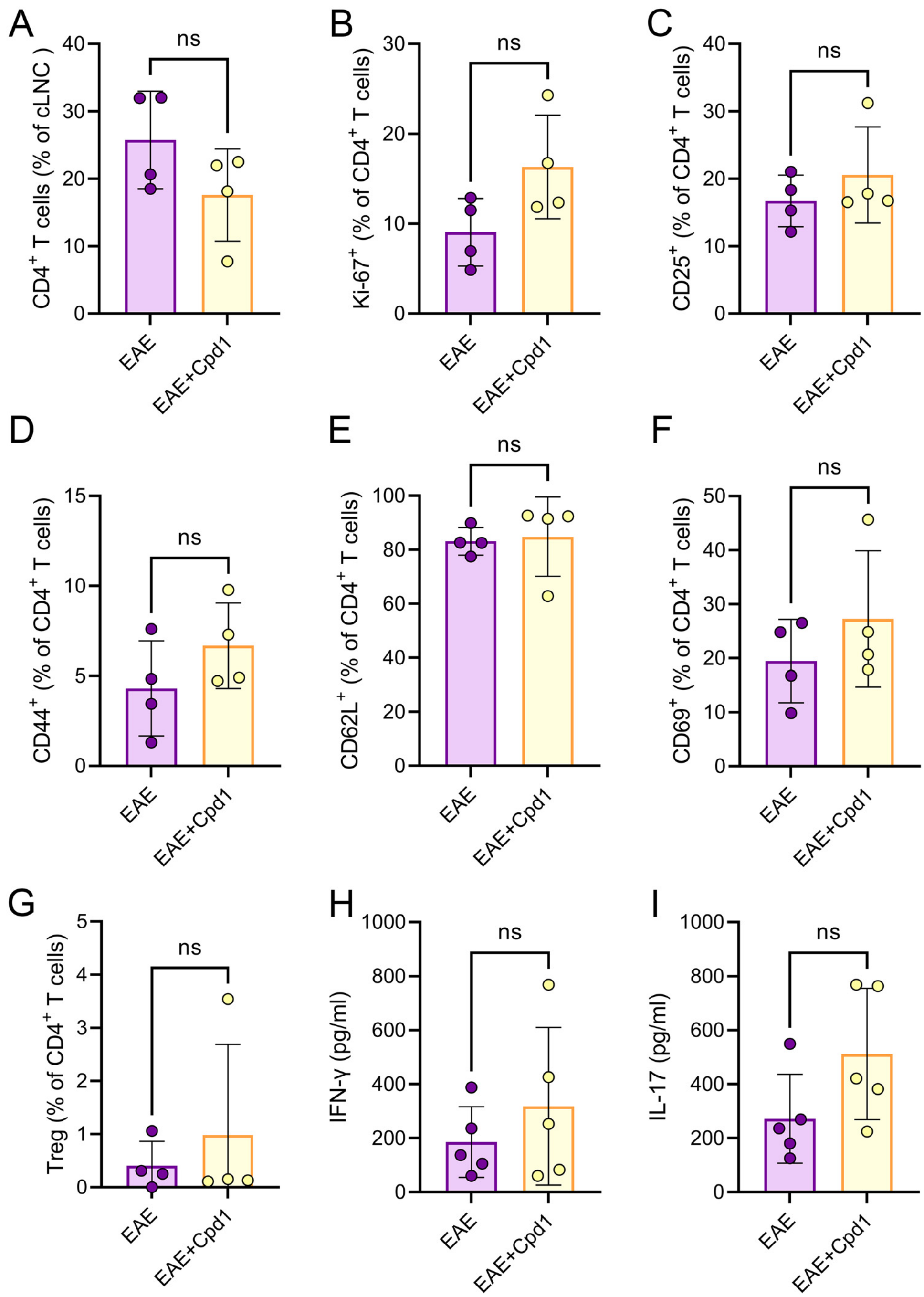

Figure EV3. Effect of Cpd1 on lymph node cells.

EAE mice were treated orally with 20 mg/kg body weight Cpd1 (EAE+Cpd1, $n = 11$) or vehicle (EAE, $n = 11$) (days 9–15). Cervical lymph node cells (cLNC) were isolated on day 17 after the immunization (**A–G**). Percentage of $CD4^+$ T cells (**A**), $Ki67^+$ cells (**B**), $CD25^+$ cells (**C**), $CD44^+$ cells (**D**), $CD62L^+$ cells (**E**), $CD69^+$ cells (**F**), and Treg (**G**). Inguinal lymph node cells were isolated on day 12 after the immunization (**H**, **I**) and stimulated with $MOG_{35\text{-}55}$ for 24 h. Subsequently, ELISA was performed for IFN-γ (**H**) and IL-17 (**I**). The data presented are biological replicates from one of two independent experiments. Data are presented as mean ± SD ($n = 4$ in (**A–G**), $n = 5$ in (**H**, **I**)) and analyzed by Student's *t* test. ns not significant.

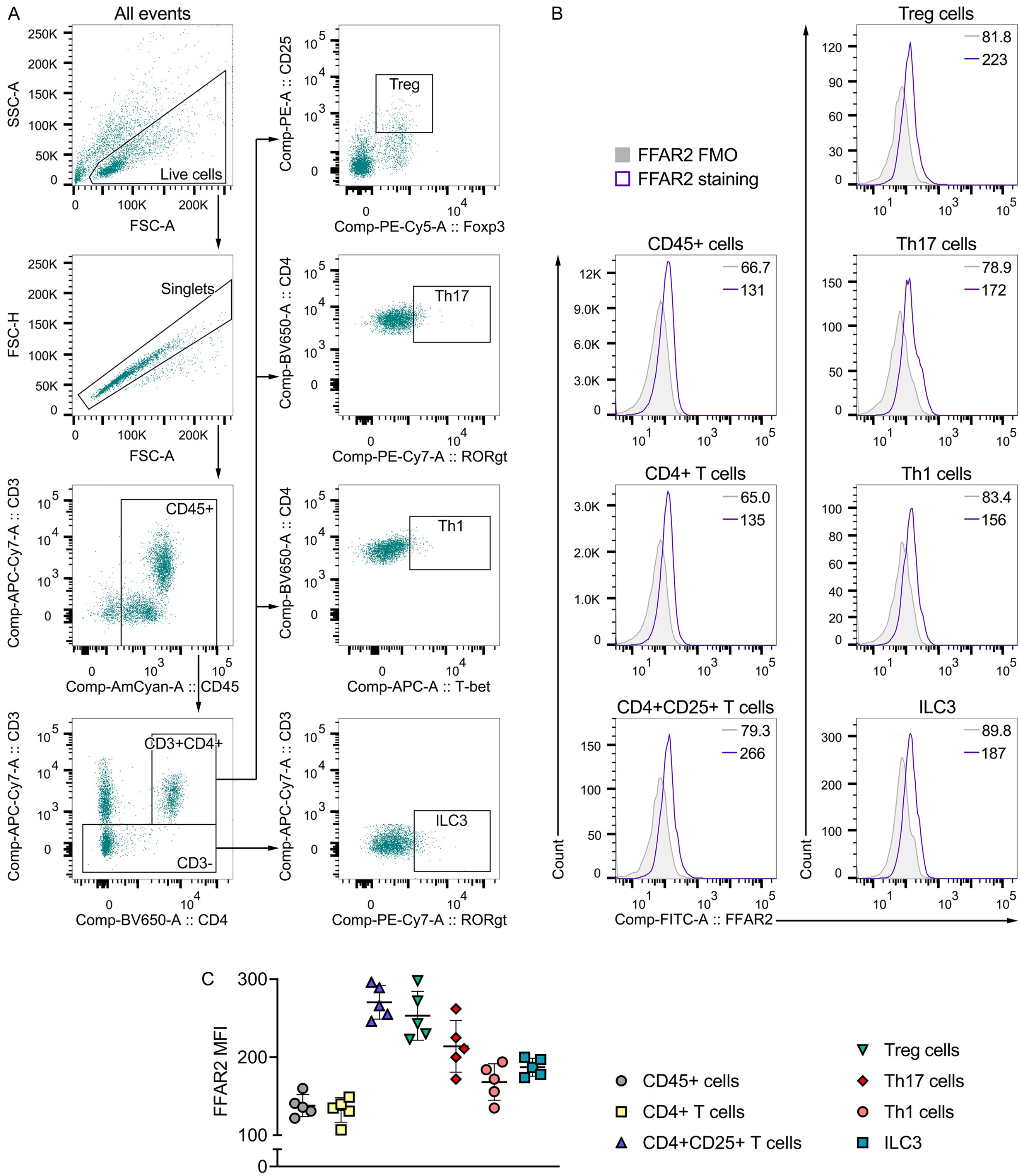
A
All events
Live cells
Singlets
CD45+
CD3+CD4+
CD3-
Treg
Th17
Th1
ILC3
B
FFAR2 FMO
FFAR2 staining
CD45+ cells
CD4+ T cells
CD4+CD25+ T cells
Treg cells
Th17 cells
Th1 cells
ILC3
Count
Comp-FITC-A :: FFAR2
C
FFAR2 MFI
CD45+ cells
CD4+ T cells
CD4+CD25+ T cells
Treg cells
Th17 cells
Th1 cells
ILC3

 © The Author(s)

Figure EV4. FFAR2 expression in SILP immune cells.

Immune cells were isolated from the SILP of mice on day 12 after EAE immunization and analyzed by flow cytometry. Gating strategy used to analyze SILP immune cells ($CD45^+$) for $CD4^+$ T-lymphocyte ($CD3^+CD4^+$) subsets, i.e. activated ($CD25^+$), Treg ($CD25^+Foxp3^+$), Th17 ($ROR\gamma t^+$) and Th1 ($T\text{-}bet^+$) cells, and ILC3 ($CD3^-ROR\gamma t^+$) (**A**). Gating boundary for activated $CD25^+$ cells within $CD4^+$ T lymphocytes was set as in the upper right dot plot. Representative flow cytometry histograms indicate FFAR2 staining (open purple histograms) and FFAR2 fluorescence minus one control (FMO, filled gray histogram), along with corresponding mean fluorescence intensity (MFI) values, for distinct SILP immune cell subpopulations (delineated as shown in (**A**)) (**B**). Scatter plot represents individual FFAR2 MFI values on designated SILP cell subsets, while horizontal bars indicate means ± SD ($n = 5$) (**C**). The data presented are biological replicates from one experiment.

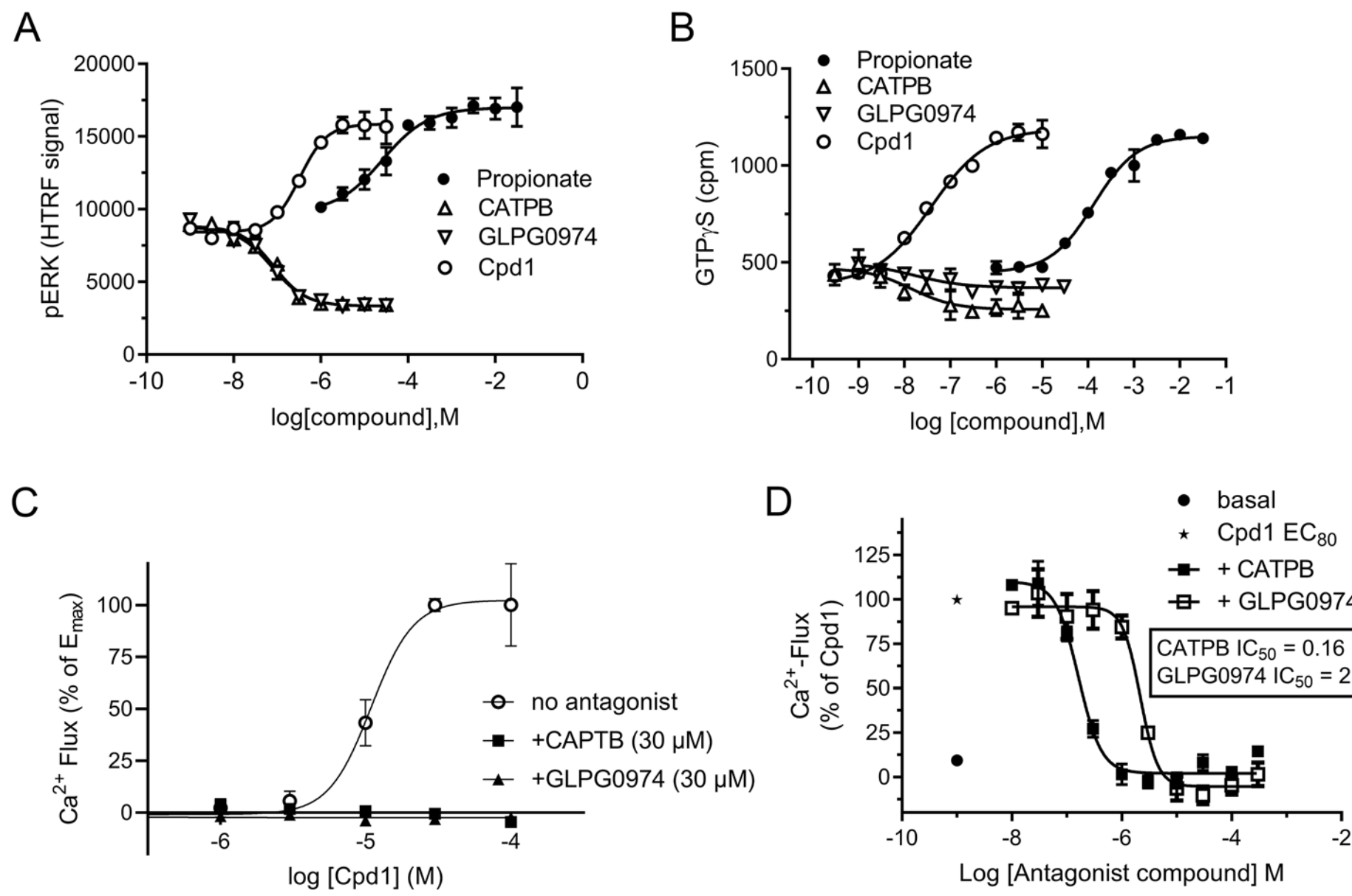

Figure EV5. Cpd1 receptor activation and selectivity at FFAR2.

Assays conducted on CHO cells stably expressing *h*FFAR2, pERK assay (**A**) and [^{35}S]GTPγS assay (**B**) concentration-response curves for the agonists Cpd1 and propionate and the reference antagonists CAPTB and GLPG0974. Cpd1-induced Ca^{2+} flux concentration-response on human polymorphonuclear cells (**C**) is fully blocked by increasing concentrations of the FFAR2 reference antagonists (**D**).

 © The Author(s)

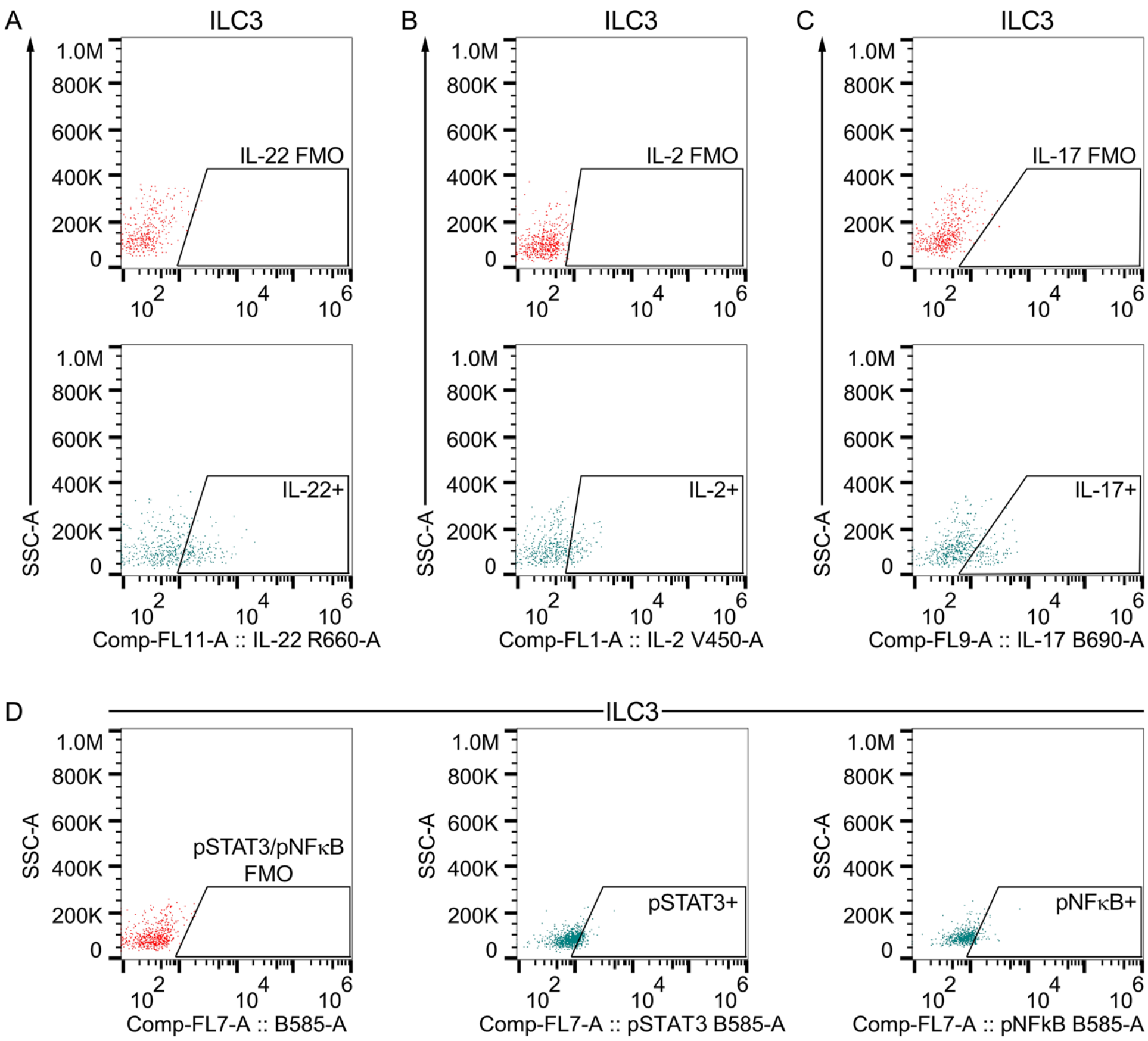

Figure EV6. Flow cytometry gating strategies used to analyze SILP ILC3 subsets.

Representative flow cytometry dot plots indicate immunostaining of IL-22 (**A**, lower), IL-2 (**B**, lower), IL-17 (**C**, lower), phosphorylated STAT3 (**D**, middle), and phosphorylated NFκB (**D**, right) in SILP ILC3. Gating boundaries for all indicated ILC3 were checked against corresponding fluorescence minus one (FMO) controls (**A**–**C**, upper; **D**, left). SILP ILC3 were gated as illustrated in Fig EV3.

© The Author(s)

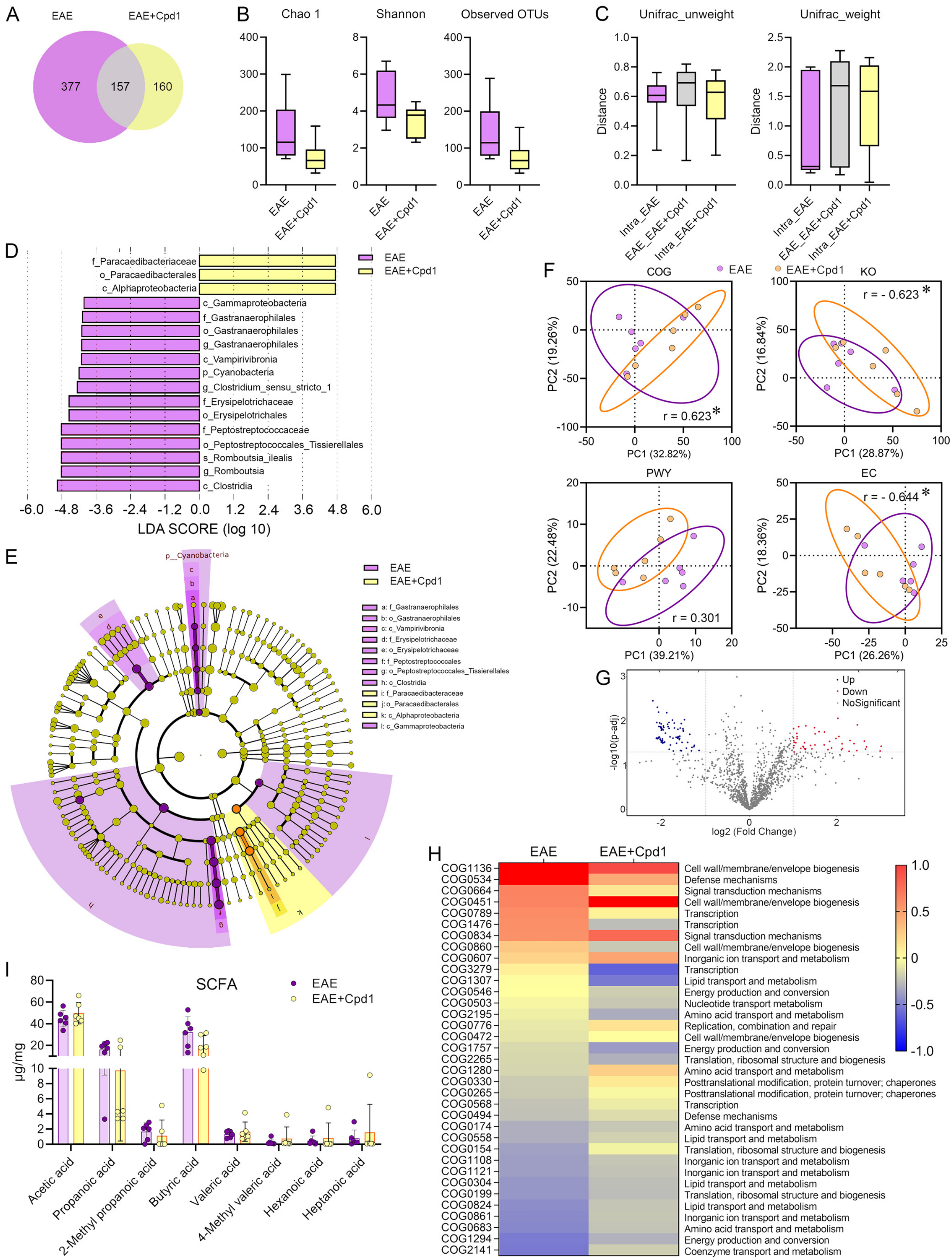
A
EAE
EAE+Cpd1
377
157
160
B
Chao 1
Shannon
Observed OTUs
EAE
EAE+Cpd1
C
Unifrac_unweight
Unifrac_weight
Distance
Intra_EAE
EAE_EAE+Cpd1
Intra_EAE+Cpd1
D
f_Paracaedibacteriaceae
o_Paracaedibacterales
c_Alphaproteobacteria
c_Gammaproteobacteria
f_Gastranaerophilales
o_Gastranaerophilales
g_Gastranaerophilales
c_Vampirivibrionia
p_Cyanobacteria
g_Clostridium_sensu_stricto_1
f_Erysipelotrichaceae
o_Erysipelotrichales
f_Peptostreptococcaceae
o_Peptostreptococcales_Tissierellales
s_Romboutsia_ilealis
g_Romboutsia
c_Clostridia
LDA SCORE (log 10)
E
p_Cyanobacteria
a: f_Gastranaerophilales
b: o_Gastranaerophilales
c: c_Vampirivibrionia
d: f_Erysipelotrichaceae
e: o_Erysipelotrichales
f: f_Peptostreptococcales
g: o_Peptostreptococcales_Tissierellales
h: c_Clostridia
i: f_Paracaedibacteraceae
j: o_Paracaedibacterales
k: c_Alphaproteobacteria
l: c_Gammaproteobacteria
F
COG
KO
PWY
EC
EAE
EAE+Cpd1
r = 0.623*
r = - 0.623*
r = 0.301
r = - 0.644*
PC1 (32.82%)
PC2 (19.26%)
PC1 (28.87%)
PC2 (16.84%)
PC1 (39.21%)
PC2 (22.48%)
PC1 (26.26%)
PC2 (18.36%)
G
Up
Down
NoSignificant
-log10(p-adj)
log2 (Fold Change)
H
EAE
EAE+Cpd1
COG1136 Cell wall/membrane/envelope biogenesis
COG0534 Defense mechanisms
COG0664 Signal transduction mechanisms
COG0451 Cell wall/membrane/envelope biogenesis
COG0789 Transcription
COG1476 Transcription
COG0834 Signal transduction mechanisms
COG0860 Cell wall/membrane/envelope biogenesis
COG0607 Inorganic ion transport and metabolism
COG3279 Transcription
COG1307 Lipid transport and metabolism
COG0546 Energy production and conversion
COG0503 Nucleotide transport metabolism
COG2195 Amino acid transport and metabolism
COG0776 Replication, combination and repair
COG0472 Cell wall/membrane/envelope biogenesis
COG1757 Energy production and conversion
COG2265 Translation, ribosomal structure and biogenesis
COG1280 Amino acid transport and metabolism
COG0330 Posttranslational modification, protein turnover; chaperones
COG0265 Posttranslational modification, protein turnover; chaperones
COG0568 Transcription
COG0494 Defense mechanisms
COG0174 Amino acid transport and metabolism
COG0558 Lipid transport and metabolism
COG0154 Translation, ribosomal structure and biogenesis
COG1108 Inorganic ion transport and metabolism
COG1121 Inorganic ion transport and metabolism
COG0304 Lipid transport and metabolism
COG0199 Translation, ribosomal structure and biogenesis
COG0824 Lipid transport and metabolism
COG0861 Inorganic ion transport and metabolism
COG0683 Amino acid transport and metabolism
COG1294 Energy production and conversion
COG2141 Coenzyme transport and metabolism
I
SCFA
EAE
EAE+Cpd1
μg/mg
Acetic acid
Propanoic acid
2-Methyl propanoic acid
Butyric acid
Valeric acid
4-Methyl valeric acid
Hexanoic acid
Heptanoic acid

Figure EV7. Effect of Cpd1 treatment on gut microbiota and SCFA.

EAE mice were treated orally with 20 mg/kg body weight Cpd1 (EAE+Cpd1) or vehicle (EAE) from day 9. On day 12, samples were taken from the terminal ileum ($n = 5$ per group) for 16S rRNA sequencing (**A–H**) or from the cecal contents ($n = 6$ per group) for SCFA measurement (**I**). Venn diagram showing the number of exclusive and shared amplicon sequence variants (ASVs) (**A**). Alpha diversity indicators including Chao1 and Shannon indexes, and the number of operational taxonomic units (OTUs) (**B**). Box plots of sample-distance based on unweighted and weighted UniFrac analysis at the ASVs level, the Kruskal–Wallis test showed no statistically significant differences among groups (**C**). Taxonomic groups with LDA scores >4.0 with $P < 0.05$; p, phylum; c, class; o, order; f, family; g, genus; s, species (**D**). The cladogram shows the phylogenetic relationship of the significant ASVs associated with each group (**E**). Violet and yellow indicate taxa enriched in EAE versus EAE+Cpd1 groups, respectively. PC analyses, two-tailed Pearson's correlation test, $^*P < 0.05$ (**F**). Volcano plot showing upregulated and downregulated functions, $|\text{log2fold change}| \geq 0$ (**G**). COG analyses (**H**). SCFA contents determined by GC/MS (**I**). The data presented are biological replicates from one experiment. Data in (**B**, **C**) are presented as box and whiskers (min to max). Data in (**I**) are presented as mean ± SD ($n = 6$), the Mann–Whitney test showed no statistically significant difference between EAE and EAE+Cpd1 groups.

© The Author(s)
